# Highly pathogenic avian influenza A(H5N1) virus infection in farmed minks, Spain, October 2022

**DOI:** 10.2807/1560-7917.ES.2023.28.3.2300001

**Published:** 2023-01-19

**Authors:** Montserrat Agüero, Isabella Monne, Azucena Sánchez, Bianca Zecchin, Alice Fusaro, María José Ruano, Manuel del Valle Arrojo, Ricardo Fernández-Antonio, Antonio Manuel Souto, Pedro Tordable, Julio Cañás, Francesco Bonfante, Edoardo Giussani, Calogero Terregino, Jesús Javier Orejas

**Affiliations:** 1 Laboratorio Central de Veterinaria (LCV), Ministry of Agriculture, Fisheries and Food, Algete, Madrid, Spain; 2 Istituto Zooprofilattico Sperimentale delle Venezie (IZSVe), Legnaro, Italy; 3 Counselling of Health, Xunta de Galicia, A Coruña, Spain; 4 Galician Mink Breeders Association, Santiago de Compostela, A Coruña, Spain; 5 Livestock Service, Counselling of Rural Affairs, Xunta de Galicia, A Coruña, Spain; 6 Animal Health Service, Counselling of Rural Affairs, Xunta de Galicia, A Coruña, Spain

**Keywords:** mink, influenza A, H5N1, HPAI, Spain, mammal, mutations, PB2

## Abstract

In October 2022, an outbreak in Europe of highly pathogenic avian influenza (HPAI) A(H5N1) in intensively farmed minks occurred in northwest Spain. A single mink farm hosting more than 50,000 minks was involved. The identified viruses belong to clade 2.3.4.4b, which is responsible of the ongoing epizootic in Europe. An uncommon mutation (T271A) in the PB2 gene with potential public health implications was found. Our investigations indicate onward mink transmission of the virus may have occurred in the affected farm.

This report describes an outbreak of highly pathogenic avian influenza (HPAI) A(H5N1) detected in intensively farmed minks in Europe, which occurred in the Galicia region in northwest Spain in October 2022. We present an in-depth description of the epidemiological, clinical and genetic investigations of this outbreak affecting a single farm and discuss public health implications.

## Outbreak description and sampling

On the first week of October, an acute increase in the mortality rate (0.77% vs an expected range of 0.2–0.3%) was identified at an American mink (*Neovison vison*) farm in the municipality of Carral, in the province A Coruña, Galicia, Spain.

Therefore, on 4 October 2022, the farm clinical veterinarian collected oropharyngeal swabs from two affected animals. The samples, analysed at the Central Veterinary Laboratory (LCV) of Algete (Ministry of Agriculture, Fisheries and Food (MAPA)), tested negative by real-time reverse transcription (RT)-PCR for severe acute respiratory syndrome coronavirus 2 (SARS-CoV-2) [1], and positive by real-time RT-PCR for HPAI A(H5N1) virus [2,3]. Post-mortem examination revealed haemorrhagic pneumonia or red hepatisation of the lungs as the most notable lesions.

On 13 October, the animal health services conducted a census to estimate the number of minks at the investigated farm, which amounted to 51,986 animals. The minks were housed in wire netting cages placed in rows and situated in a series of over 30 partially open barns, which provided overhead protection but not total shelter of their sides. The minks were fed with raw fish and poultry by-products, cereals and blood meal. Poultry farms and avian slaughterhouses supplying the poultry by-products were located in Galicia. Up to 10 January 2023, H5N1 poultry outbreaks have not been reported from this region.

The mortality rate increased on a weekly basis until reaching a peak in the week of 17–23 October (4.3%). On the first week of October, the mortality was observed in the barns close to the manure storage facility. The mortality pattern at that time was characterised by multiple ‘hot spots’ within the affected barns consisting of 2–4 pens where all the animals died within a period of 1–2 days. In the following weeks, the mortality increased also in the neighbouring barns and the whole premises was affected. Clinical signs of infection in minks included loss of appetite, hypersalivation, depression, bloody snout and neurological manifestations such as ataxia and tremors. On 18 and 26 October, additional sampling was implemented across distinct areas of the farm prioritising the barns presenting the highest daily mortality and the presence of the H5N1 virus was confirmed with high viral load (based on quantification cycle (Cq) values) in oropharyngeal (n = 9) or rectal swabs (n = 9) and/or lung samples (n = 3) from 12 of 13 individual minks sampled (see Supplementary Table S1 for details on results of RT-PCR for influenza A virus in organs and swabs collected in minks).

Of note, in the weeks preceding the identification of the mink outbreak, several cases of HPAI H5N1 were reported in wild birds found sick or dead (25 common gannets (*Morus bassanus*) and 2 seagulls (*Larus michaelis*)) along the coasts near A Coruña and in the neighbouring province of Lugo [4]. As a consequence of these events, the suspicion of H5N1 virus infection in minks was raised and the specific diagnostic flowchart to identify the disease was prioritised together with the molecular investigations to exclude SARS-CoV-2 infection as foreseen by the Commission Implementing Decision (EU) 2021/788 [5].

## Genome characterisation

**Figure 1:**
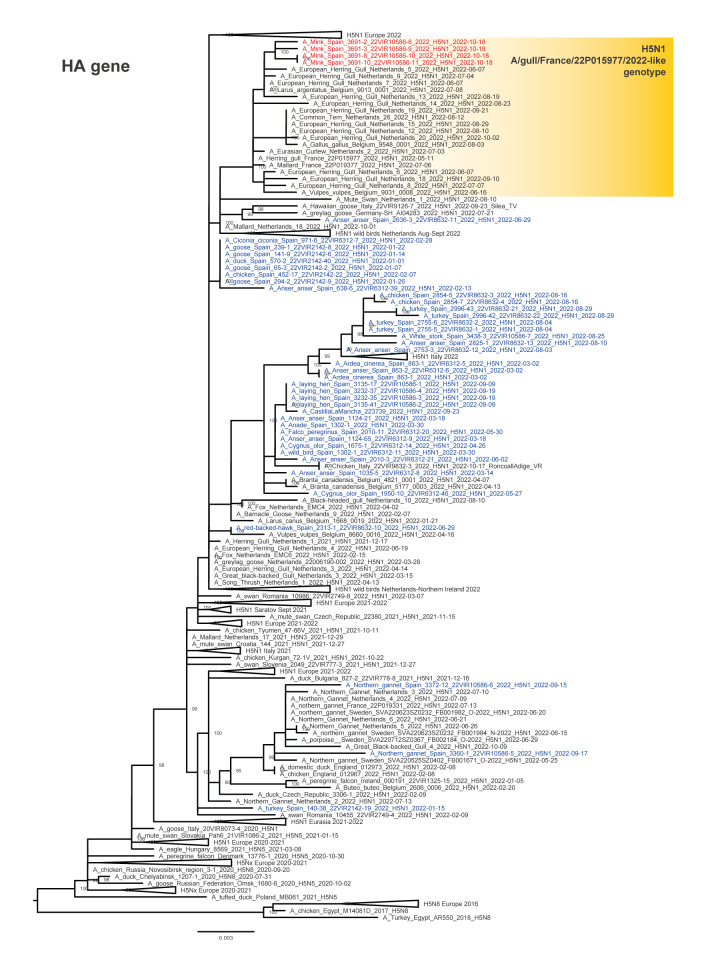
Maximum likelihood phylogenetic tree of the haemagglutinin gene segment of highly pathogenic avian influenza H5N1 viruses from minks (n = 4) and avian species (n = 38) in Spain as well as H5 sequences collected from 23 European countries (n = 292)^a^, October 2021–October 2022

HA: haemagglutinin; HPAI: highly pathogenic avian influenza.

^a^ Sequences were downloaded from GISAID EpiFlu database on 11 November 2022.

**Figure 2:**
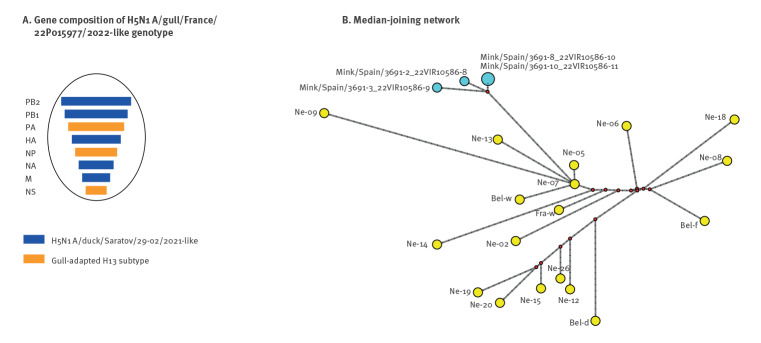
Genetic network of complete genome sequences of viruses belonging to the highly pathogenic avian influenza H5N1 virus A/gull/France/22P015977/2022-like genotype, from four European countries, May–October 2022 (n = 22)

Four of the nine H5N1 virus-positive oropharyngeal swabs collected on 18 October were submitted to the European Reference laboratory (EURL) for avian influenza (AI) in Italy (Istituto Zooprofilattico Sperimentale delle Venezie) for genetic characterisation. Whole genome sequences were generated (GISAID accession numbers: EPI2220590–EPI2220621) and phylogenetically analysed. The analysis of the haemagglutinin (HA) gene segment showed that the HPAI H5N1 viruses from the minks belong to clade 2.3.4.4b (Figure 1). Clustering of the four characterised viral genomes from minks indicates that they are highly related (similarity ranging from 99.8% to 100%) and belong to the A/gull/France/22P015977/2022-like genotype [6] (Figure 2).

The tree was obtained by using IQTree v.1.6.6, and 1,000 ultra-fast bootstrap replicates. The HPAI H5N1 viruses from mink are coloured in red, all the other HPAI H5N1 viruses identified in Spain in 2022 are coloured in blue. The yellow box shows the viruses belonging to the H5N1 A/gull/France/22P015977/2022-like genotype [6]. Some of the genetic groups were collapsed for clarity.

Viruses were collected from birds and mammals between May and October 2022 in the Netherlands (n = 14), Belgium (n = 3), France (n = 1), Spain (minks; n = 4).

A. Schematic representation of the gene composition of the viruses belonging to the H5N1 virus A/ gull/France/22P015977/2022-like genotype.

B. Genetic network generated using the median-joining method implemented in NETWORK 10.2.0.0 (https://www.fluxus-engineering.com) [17], for the eight concatenated genes of H5N1 viruses belonging to the H5N1 A/gull/France/22P015977/2022-like genotype. Each virus is represented with a circle, whose size is proportional to the number of identical viruses. The length of the branches is proportional to the number of nucleotide differences between two viruses. Viruses from minks are coloured in light blue, viruses from the Netherlands (Ne), Belgium (Bel) and France (Fra) are coloured in yellow. All sequences are available in the GISAID EpiFlu database (ID: EPI_ISL_14494796, EPI_ISL_14494351, EPI_ISL_14494933, EPI_ISL_13519451, EPI_ISL_13902858, EPI_ISL_15069401, EPI_ISL_13778468, EPI_ISL_13778469, EPI_ISL_13778474, EPI_ISL_14163712, EPI_ISL_14163714, EPI_ISL_15069398, EPI_ISL_15088296, EPI_ISL_15088297, EPI_ISL_15088304, EPI_ISL_15364789, EPI_ISL_15579535, EPI_ISL_15579539). Red dots represent median vectors, while the dashes correspond to the nucleotide differences between two viruses.

Since May 2022 this H5N1 virus genotype, which originated from reassortment events for the PA, NP, NS gene segments with viruses of the gull-adapted H13 subtype, has been identified in wild birds (mainly European herring gulls) in the Netherlands, Belgium and France, as well as in a chicken outbreak and in a fox (*Vulpes vulpes*) in Belgium. Based on the currently available genetic data, this is the first identification of this genotype in Spain. The mink viruses showed between 8 and 9 amino acid differences in the PB2, PB1, PA, NA, NS2, M2 and PB1-F2 with the most closely related H5N1 viruses (Table). In particular, all of the viruses from minks present an alanine (A) at position 271 of PB2 (T271A), which enhances the polymerase activity of influenza A viruses in mammalian host cells and mice [7]. At the time of writing, of the HPAI H5 viruses sequenced in Europe since autumn 2020, this mutation was identified only in a H5N1 virus collected from an infected European polecat in the Netherlands in March 2022 (EPI_ISL_13201074). Based on the publicly available sequences, the other mutations have never (PB1–388R, NA-74S, NS2–13G) or rarely (PB1–317V, PA-56T, NA-163L, PB1-F2–30L) been identified in HPAI H5Nx viruses detected in Europe since 2021 and their biological impact is unknown.

**Table:** Amino acid differences identified between the highly pathogenic avian influenza A(H5N1) viruses from minks (n = 4) and the most closely related H5N1 viruses in Europe (n = 18), May–October 2022

Viruses	Amino acid mutations															
	PB2			PB1				PA		HA	NA			M2	NS2	PB1-F2
	271	451	658	175	181	317	388	86	665	390	74	163	396	31	43	30
A/Mink/Spain/3691–8_22VIR10586–10/2022	**A**	V	Y	N	I	**V**	**R**	**T**	L	**M**	**S**	**L**	M	N	**G**	**L**
A/Mink/Spain/3691–10_22VIR10586–11/2022	**A**	V	Y	N	I	**V**	**R**	**T**	L	**M**	**S**	**L**	M	N	**G**	**L**
A/Mink/Spain/3691–2_22VIR10586–8/2022	**A**	V	**H**	N	I	**V**	**R**	**T**	L	I	**S**	**L**	M	N	**G**	**L**
A/Mink/Spain/3691–3_22VIR10586–9/2022	**A**	V	Y	N	I	**V**	**R**	**T**	**M**	I	**S**	**L**	M	N	**G**	**L**
A/Eurasian_Curlew/Netherlands/2/2022	T	nd	Y	D	I	M	K	M	L	I	F	V	I	S	D	P
A/European_Herring_Gull/Netherlands/5/2022	T	nd	Y	N	I	M	K	M	L	I	F	V	M	N	D	P
A/European_Herring_Gull/Netherlands/6/2022	T	nd	Y	D	I	M	K	M	L	I	F	V	I	S	D	P
A/European_Herring_Gull/Netherlands/7/2022	T	nd	Y	N	I	M	K	M	L	I	F	V	M	N	D	P
A/European_Herring_Gull/Netherlands/9/2022	T	nd	Y	N	I	M	K	M	L	I	F	V	M	N	D	P
A/European_Herring_Gull/Netherlands/12/2022	T	nd	Y	D	M	M	K	M	L	I	F	V	I	S	D	P
A/European_Herring_Gull/Netherlands/13/2022	T	nd	Y	N	I	M	K	M	L	I	F	V	M	N	D	P
A/European_Herring_Gull/Netherlands/14/2022	T	nd	Y	N	I	M	K	M	L	I	F	V	M	N	D	P
A/European_Herring_Gull/Netherlands/18/2022	T	nd	Y	D	M	M	K	M	L	I	F	V	I	S	D	P
A/Common_Tern/Netherlands/26/2022	T	nd	Y	D	M	M	K	M	L	I	F	V	I	S	D	P
A/Larus_argentatus/Belgium/9013_0001/2022	T	I	Y	N	I	M	K	M	L	I	F	V	M	N	D	P
A/Vulpes_vulpes/Belgium/9031_0008/2022	T	I	Y	D	M	M	K	M	L	I	F	V	I	S	D	P
A/gull/France/22P015977/2022	T	V	Y	N	I	M	K	M	L	I	F	V	I	S	D	P
A/European_Herring_Gull/Netherlands/8/2022	T	nd	Y	D	M	M	K	M	L	I	F	V	I	S	D	P
A/European_Herring_Gull/Netherlands/15/2022	T	nd	Y	D	M	M	K	M	L	I	F	V	I	S	D	P
A/European_Herring_Gull/Netherlands/19/2022	T	nd	Y	D	M	M	K	M	L	I	F	V	I	S	D	P
A/European_Herring_Gull/Netherlands/20/2022	T	nd	Y	D	M	M	K	M	L	I	F	V	I	S	D	P
A/Gallus_gallus/Belgium/9548_0001/2022	T	I	Y	D	M	M	K	M	L	I	F	V	I	S	D	P
References	[7]	nr	nr	nr	nr	nr	nr	nr	nr	nr	nr	nr	nr	[16]	nr	nr

nd: not determined; nr: no reference.

Viruses represented were collected between May and October 2022 from the Netherlands (n = 14), Belgium (n = 3), France (n = 1) and Spain (minks; n = 4). Mutations identified only in samples from minks are highlighted in bold.

## Public health measures

Culling activities started soon after the official order by animal health services on 18 October 2022. Animals were culled in batches of 150–200 animals. By 17 November 2022, all minks at the infected premises were culled and all the carcasses, fomites and waste were destroyed. 

The mink farm had a staff of 12 workers, 11 of whom had been in contact with the animals and were also involved in the culling activities. On 13 and 14 October, nasopharyngeal swabs were taken from the 11 asymptomatic workers and all tested negative for avian influenza virus (AIV). A semi-quarantine regimen, intended to avoid any contact with other people, was employed to the exposed workers for 10 days from their last contact with the animals or the farm. In addition, the workers and their cohabitants were instructed to immediately inform public health authorities in case of influenza-like illness, such as runny/stuffy nose, fever, sore throat, cough, muscle or body aches, headaches, in order to initiate testing and follow-up. On 2 November 2022, one of the workers had a runny nose. Real-time RT-PCR against AIV was performed on a nasopharyngeal sample, yielding negative results. Antiviral post-exposure prophylaxis was not prescribed, as more than 48 h had already passed since the potential HPAI H5N1 virus exposure.

Of note, from April 2020 onwards, following the first identification of SARS-CoV-2 infection in mink farms in the Netherlands [8], the use of a face mask was made compulsory for all farm workers on mink farms in Spain. Since the SARS-CoV-2/HPAI suspicion was raised on 4 October 2022, increased biosafety measures including the use of disposable overalls, face shields, face mask changing (twice per day) and frequent handwashing were applied in the farm. Workwear was washed at the farm and showering before leaving the farm was also encouraged. All unnecessary activities at the premises were discontinued.

## Discussion

We present, to the best of our knowledge, the first report of clade 2.3.4.4b HPAI H5N1 virus infection of minks farmed for their fur in Europe. The viruses identified presented the highest similarity with strains of the A/gull/France/22P015977/2022-like genotype, which has already been described in multiple wild bird species and sporadically in poultry across northern Europe [6]. However, the viruses detected at the mink farm are distinguished from all the clade 2.3.4.4b H5N1 viruses characterised thus far in the avian population in Europe as they bear an uncommon mutation (T271A) in the PB2 gene, which may have public health implications. Indeed, the same mutation is present in the avian-like PB2 gene of the 2009 pandemic swine-origin influenza A(H1N1) virus (H1N1pdm). Zhang et al. [9] demonstrated that mutations to the avian virus-conserved residue (threonine, T) reduced polymerase activity and abolished the H1N1pdm virus respiratory droplet transmission in guinea pigs. Furthermore, this study shows that amino acid 271A of PB2 plays a key role in virus acquisition of the mutation at position 226 of HA that confers human receptor recognition. As T271A is an uncommon amino acid change not previously identified among European HPAI H5 viruses in 2020–22, with the exception of a single H5N1 virus from a mammalian host (European polecat), this mutation could have arisen de novo in minks. However, the data available are not sufficient to exclude the possibility of an unobserved circulation of avian viruses bearing this substitution in the avian population.

Our findings also indicate that an onward transmission of the virus to other minks may have taken place in the affected farm. This is suggested by the increasing number of infected animals identified after the confirmation of the disease and the progression of the infection from the initially affected area to the entire holding. Additional experimental studies are ongoing to further explore virulence and transmissibility of these viruses.

The source of the outbreak remains unknown. No AI cases were reported in poultry farms supplying the poultry by-products. However, considering that the mink spillover event was coincident with a wave of H5N1 virus infections in seabirds in Galicia [4], it can be assumed that wild birds may have played a major role in the virus introduction into the farm. This hypothesis is further supported given that minks were farmed in a partially open building and may have been in contact with wild birds. Indeed, the A/gull/France/22P015977/2022-like genotype has been diagnosed in multiple seabird species across Europe, including common gannets and seagulls, which were the species involved in the H5N1 mortality events registered in Galicia in the weeks before the mink outbreak. Sequencing of the contemporary H5N1 virus-positive wild birds collected in the area will be essential to confirm this assumption.

Experimental and field evidence have demonstrated that minks are susceptible and permissive to both avian and human influenza A viruses, leading to the theory that this species could serve as a potential mixing vessel for the interspecies transmission among birds, mammals and human [10-14]. In light of this and considering the ongoing HPAI H5N1 panzootic, our findings further highlight the importance of preventing mink infection with such viruses. 

## Conclusions

Despite the criticism fur farming has recently received following SARS-CoV-2 cases in farmed minks and the spillover/spillback SARS-CoV-2 transmission events reported between minks and humans, this production sector is still common worldwide with an important economic impact [15]. For this reason, and given the concerns caused by the susceptibility of minks to emerging viruses such as HPAI H5N1 viruses and SARS-CoV-2, it is necessary to strengthen the culture of biosafety and biosecurity in this farming system and promote the implementation of ad hoc surveillance programs for influenza A viruses and other zoonotic pathogens at a global level. These interventions are instrumental to prevent contact between minks and wild animals, and to control disease transmission events from minks to farm workers and vice versa.

## Supplementary Data

1053000 Supplement
