# Supplementary material for: Highly pathogenic avian influenza A(H5N1) virus infection in farmed minks, Spain, October 2022
Source: Euro Surveill. 2023 Jan 19;28(3):2300001. doi: 10.2807/1560-7917.ES.2023.28.3.2300001 (PMC9853945; doi:10.2807/1560-7917.ES.2023.28.3.2300001)
Supplement: Supplement [file 23-00001_MONNE_SUPPLEMENT.pdf]

Supplementary material

This supplementary material is hosted by Eurosurveillance as supporting information alongside the article “Highly pathogenic avian influenza H5N1 infection in farmed minks, Spain, October 2022” on behalf of the authors who remain responsible for the accuracy and appropriateness of the content. The same standards for ethics, copyright, attributions and permissions as for the article apply. Eurosurveillance is not responsible for the maintenance of any links or email addresses provided therein.

Supplementary Table 1. RT-PCR of the influenza A virus matrix gene results in organs and swabs collected in minks. *Cycle threshold (Ct) value, cut-off value is 35.

		Ct values*		
**Animal number**	Sampling date	Oropharyngeal swab	Rectal swab	Lung
1	4th October 2022	23,6	N.A.	N.A.
2		33,2	N.A.	N.A.
3	18th October 2022	22,4	29,6	N.A.
4		20,7	31,5	N.A.
5		18,7	28,1	N.A.
6		24,8	31,0	N.A.
7		22,7	32,2	N.A.
8		18,6	26,0	N.A.
9		22,1	28,0	N.A.
10		18,9	28,5	N.A.
11		neg	neg	N.A.
12		20,9	28,8	N.A.
13		N.A.	N.A.	17,9
14	26th October 2022	N.A.	N.A.	15,6
15		N.A.	N.A.	17,1

N.A. =not available
Neg=Negative

Supplementary Table 2. Acknowledgment table of the authors, originating and submitting laboratories of the sequences from GISAID's EpiFlu™ Database on which this research is based in part. The list is detailed below (na: not available). All submitters of data may be contacted directly via www.gisaid.org

Isolate-ID	Country	Collection date	Isolate name	Originating Lab	Submitting Lab	Authors
EPI_ISL_5463793	Russian Federation	2021-Oct-06	A/chicken/Tyumen/27-31V/2021	FBUZ Center of Hygiene and Epidemiology in Tyumen oblast	State Research Center of Virology and Biotechnology (VECTOR)	Natalia,Goncharova; Ivan,Susloparov; Natalia,Kolosova; Alexey,Danilenko; Juliya,Bulanovich; Vasiliy,Marchenko; Alexander,Ryzhikov
EPI_ISL_5463794	Russian Federation	2021-Oct-06	A/chicken/Tyumen/27-39V/2021	FBUZ Center of Hygiene and Epidemiology in Tyumen oblast	State Research Center of Virology and Biotechnology (VECTOR)	Natalia,Goncharova; Ivan,Susloparov; Natalia,Kolosova; Alexey,Danilenko; Juliya,Bulanovich; Vasiliy,Marchenko; Alexander,Ryzhikov
EPI_ISL_5463795	Russian Federation	2021-Oct-06	A/chicken/Tyumen/27-40V/2021	FBUZ Center of Hygiene and Epidemiology in Tyumen oblast	State Research Center of Virology and Biotechnology (VECTOR)	Natalia,Goncharova; Ivan,Susloparov; Natalia,Kolosova; Alexey,Danilenko; Juliya,Bulanovich; Vasiliy,Marchenko; Alexander,Ryzhikov
EPI_ISL_5463796	Russian Federation	2021-Oct-06	A/chicken/Tyumen/27-42V/2021	FBUZ Center of Hygiene and Epidemiology in Tyumen oblast	State Research Center of Virology and Biotechnology (VECTOR)	Natalia,Goncharova; Ivan,Susloparov; Natalia,Kolosova; Alexey,Danilenko; Juliya,Bulanovich; Vasiliy,Marchenko; Alexander,Ryzhikov

EPI_ISL_5463804	Russian Federation	2021-Oct-07	A/chicken/Tyumen/33-45V/2021	FBUZ Center of Hygiene and Epidemiology in Tyumen oblast	State Research Center of Virology and Biotechnology (VECTOR)	Natalia,Goncharova; Ivan,Susloparov; Natalia,Kolosova; Alexey,Danilenko; Juliya,Bulanovich; Vasiliy,Marchenko; Alexander,Ryzhikov
EPI_ISL_5463805	Russian Federation	2021-Oct-07	A/goose/Tyumen/33-52V/2021	FBUZ Center of Hygiene and Epidemiology in Tyumen oblast	State Research Center of Virology and Biotechnology (VECTOR)	Natalia,Goncharova; Ivan,Susloparov; Natalia,Kolosova; Alexey,Danilenko; Juliya,Bulanovich; Vasiliy,Marchenko; Alexander,Ryzhikov
EPI_ISL_5463806	Russian Federation	2021-Oct-07	A/goose/Tyumen/33-53V/2021	FBUZ Center of Hygiene and Epidemiology in Tyumen oblast	State Research Center of Virology and Biotechnology (VECTOR)	Natalia,Goncharova; Ivan,Susloparov; Natalia,Kolosova; Alexey,Danilenko; Juliya,Bulanovich; Vasiliy,Marchenko; Alexander,Ryzhikov
EPI_ISL_5588100	Netherlands	2021-Oct-25	A/chicken/Netherlands/21037287-006010/2021	Wageningen Bioveterinary Research	Wageningen Bioveterinary Research	Beerens, Nancy; Harders, Frank; Pritz-Verschuren, Sylvia; Roose, Marit; Germeraad, Evelien; Engelsma, Marc; Heutink, Rene
EPI_ISL_5588106	Netherlands	2021-Oct-22	A/chicken/Netherlands/21037233-001/2021	Wageningen Bioveterinary Research	Wageningen Bioveterinary Research	Beerens, Nancy; Harders, Frank; Pritz-Verschuren, Sylvia; Roose, Marit; Germeraad, Evelien; Engelsma, Marc; Heutink, Rene
EPI_ISL_5804708	United Kingdom	2021-Oct-24	A/mute_swan/England/053054/2021	Animal and Plant Health Agency (APHA)	Animal and Plant Health Agency (APHA)	na
EPI_ISL_5804698	Netherlands	2021-Oct-30	A/chicken/Netherlands/21037708-006010/2021	Wageningen Bioveterinary Research	Wageningen Bioveterinary Research	Beerens, Nancy; Harders, Frank; Pritz-Verschuren, Sylvia; Roose, Marit; Germeraad, Evelien; Engelsma, Marc; Heutink, Rene
EPI_ISL_5804788	Netherlands	2021-Oct-24	A/Mute swan/Netherlands/21037283-002/2021	Wageningen Bioveterinary Research	Wageningen Bioveterinary Research	Beerens, Nancy; Harders, Frank; Pritz-Verschuren, Sylvia; Roose, Marit; Germeraad, Evelien; Engelsma, Marc; Heutink, Rene
EPI_ISL_5941469	Netherlands	2021-Nov-03	A/duck/Netherlands/21037958-001005/2021	Wageningen Bioveterinary Research	Wageningen Bioveterinary Research	Beerens, Nancy; Harders, Frank; Pritz-Verschuren, Sylvia; Roose, Marit; Germeraad, Evelien; Engelsma, Marc; Heutink, Rene

EPI_ISL_6025676	Netherlands	2021-Oct-25	A/Barnacle goose/Netherlands/21037293-001/2021	Wageningen Bioveterinary Research	Wageningen Bioveterinary Research	Beerens, Nancy; Harders, Frank; Pritz-Verschuren, Sylvia; Roose, Marit; Germeraad, Evelien; Engelsma, Marc; Heutink, Rene
EPI_ISL_5945412	Netherlands	2021-Oct-25	A/European Herring Gull/Netherlands/21037412-002/2021	Wageningen Bioveterinary Research	Wageningen Bioveterinary Research	Beerens, Nancy; Harders, Frank; Pritz-Verschuren, Sylvia; Roose, Marit; Germeraad, Evelien; Engelsma, Marc; Heutink, Rene
EPI_ISL_5942524	Netherlands	2021-Oct-31	A/chicken/Netherlands/21037750-001005/2021	Wageningen Bioveterinary Research	Wageningen Bioveterinary Research	Beerens, Nancy; Harders, Frank; Pritz-Verschuren, Sylvia; Roose, Marit; Germeraad, Evelien; Engelsma, Marc; Heutink, Rene
EPI_ISL_6101847	Netherlands	2021-Nov-05	A/duck/Netherlands/21038151-001005/2021	Wageningen Bioveterinary Research	Wageningen Bioveterinary Research	Beerens, Nancy; Harders, Frank; Pritz-Verschuren, Sylvia; Roose, Marit; Germeraad, Evelien; Engelsma, Marc; Heutink, Rene
EPI_ISL_6101848	Netherlands	2021-Nov-07	A/chicken/Netherlands/21038165-006010/2021	Wageningen Bioveterinary Research	Wageningen Bioveterinary Research	Beerens, Nancy; Harders, Frank; Pritz-Verschuren, Sylvia; Roose, Marit; Germeraad, Evelien; Engelsma, Marc; Heutink, Rene
EPI_ISL_6101868	Netherlands	2021-Oct-27	A/black-headed gull/Netherlands/21037589-002/2021	Wageningen Bioveterinary Research	Wageningen Bioveterinary Research	Beerens, Nancy; Harders, Frank; Pritz-Verschuren, Sylvia; Roose, Marit; Germeraad, Evelien; Engelsma, Marc; Heutink, Rene
EPI_ISL_6101869	Netherlands	2021-Oct-28	A/goose/Netherlands/21037720-001/2021	Wageningen Bioveterinary Research	Wageningen Bioveterinary Research	Beerens, Nancy; Harders, Frank; Pritz-Verschuren, Sylvia; Roose, Marit; Germeraad, Evelien; Engelsma, Marc; Heutink, Rene
EPI_ISL_6328001	Netherlands	2021-Oct-19	A/greylag goose/Netherlands/21037497-001/2021	Wageningen Bioveterinary Research	Wageningen Bioveterinary Research	Beerens, Nancy; Harders, Frank; Pritz-Verschuren, Sylvia; Roose, Marit; Germeraad, Evelien; Engelsma, Marc; Heutink, Rene
EPI_ISL_6328009	Netherlands	2021-Nov-01	A/swan/Netherlands/21037791-001/2021	Wageningen Bioveterinary Research	Wageningen Bioveterinary Research	Beerens, Nancy; Harders, Frank; Pritz-Verschuren, Sylvia; Roose, Marit; Germeraad, Evelien; Engelsma, Marc; Heutink, Rene
EPI_ISL_6328022	Netherlands	2021-Nov-02	A/chicken/Netherlands/21037907-006010/2021	Wageningen Bioveterinary Research	Wageningen Bioveterinary Research	Beerens, Nancy; Harders, Frank; Pritz-Verschuren, Sylvia; Roose, Marit; Germeraad, Evelien; Engelsma, Marc; Heutink, Rene
EPI_ISL_6328036	Netherlands	2021-Nov-14	A/chicken/Netherlands/21038675-001005/2021	Wageningen Bioveterinary Research	Wageningen Bioveterinary Research	Beerens, Nancy; Harders, Frank; Pritz-Verschuren, Sylvia; Roose, Marit; Germeraad, Evelien; Engelsma, Marc; Heutink, Rene
EPI_ISL_6328409	Czech Republic	2021-Nov-01	A/mute swan/Czech Republic/21312/2021	State Veterinary Institute Prague	State Veterinary Institute Prague	Nagy,Alexander;Cernikova,Lenka;Stara,Martina
EPI_ISL_6375134	Netherlands	2021-Nov-02	A/waterfowl/Netherlands/21037914-006/2021	Wageningen Bioveterinary Research	Wageningen Bioveterinary Research	Beerens, Nancy; Harders, Frank; Pritz-Verschuren, Sylvia; Roose, Marit; Germeraad, Evelien; Engelsma, Marc; Heutink, Rene
EPI_ISL_6507374	Croatia	2021-Nov-12	A/mute swan/Croatia/100/2021	Croatian Veterinary Institute, Poultry Centre	Croatian Veterinary Institute	Savić, Vladimir
EPI_ISL_6511912	Czech Republic	2021-Oct-22	A/goose/Czech Republic/20689-27T/2021	State Veterinary Institute Prague	State Veterinary Institute Prague	Nagy,Alexander;Cernikova,Lenka;Stara,Martina

EPI_ISL_6512576	Czech Republic	2021-Oct-22	A/goose/Czech Republic/20689-28T/2021	State Veterinary Institute Prague	State Veterinary Institute Prague	Nagy,Alexander;Cernikova,Lenka;Stara,Martina
EPI_ISL_6590766	France	2021-Nov-08	A/swan/France/21P012384/2021	Anses (Ploufragan-Plouzané)	ANSES Agence Nationale De Securite Sanitaire De L'alimentation	na
EPI_ISL_6596216	Sweden	2021-Nov-02	A/barnacle goose/Sweden/SVA211102SZ0402/FB004395/M-2021	National Veterinary Institute, SVA	National Veterinary Institute	na
EPI_ISL_6599075	Sweden	2021-Nov-01	A/greylag goose /Sweden/SVA211103SZ0398/FB004410/M-2021	National Veterinary Institute, SVA	National Veterinary Institute	na
EPI_ISL_6599895	Sweden	2021-Oct-29	A/common pheasant /Sweden/SVA211104SZ0320/FB004417/M-2021	National Veterinary Institute, SVA	National Veterinary Institute	na
EPI_ISL_6600745	Sweden	2021-Oct-29	A/common pheasant /Sweden/SVA211104SZ0320/FB004418/M-2021	National Veterinary Institute, SVA	National Veterinary Institute	na
EPI_ISL_6600769	Sweden	2021-Oct-29	A/common buzzard /Sweden/SVA211104SZ0320/FB004419/M-2021	National Veterinary Institute, SVA	National Veterinary Institute	na
EPI_ISL_6600943	Sweden	2021-Nov-09	A/great black-backed gull/Sweden/SVA211109SZ0434/FB004445/M-2021	National Veterinary Institute, SVA	National Veterinary Institute	na
EPI_ISL_6675733	Netherlands	2021-Nov-21	A/chicken/Netherlands/21039174-001005/2021	Wageningen Bioveterinary Research	Wageningen Bioveterinary Research	Beerens, Nancy; Harders, Frank; Pritz-Verschuren, Sylvia; Roose, Marit; Germeraad, Evelien; Engelsma, Marc; Heutink, Rene
EPI_ISL_6675739	Netherlands	2021-Nov-19	A/chicken/Netherlands/21039108-001005/2021	Wageningen Bioveterinary Research	Wageningen Bioveterinary Research	Beerens, Nancy; Harders, Frank; Pritz-Verschuren, Sylvia; Roose, Marit; Germeraad, Evelien; Engelsma, Marc; Heutink, Rene
EPI_ISL_6675744	Netherlands	2021-Nov-18	A/mute swan/Netherlands/21039008-003/2021	Wageningen Bioveterinary Research	Wageningen Bioveterinary Research	Beerens, Nancy; Harders, Frank; Pritz-Verschuren, Sylvia; Roose, Marit; Germeraad, Evelien; Engelsma, Marc; Heutink, Rene
EPI_ISL_6675750	Netherlands	2021-Nov-07	A/mute swan/Netherlands/21038412-002/2021	Wageningen Bioveterinary Research	Wageningen Bioveterinary Research	Beerens, Nancy; Harders, Frank; Pritz-Verschuren, Sylvia; Roose, Marit; Germeraad, Evelien; Engelsma, Marc; Heutink, Rene
EPI_ISL_6675755	Netherlands	2021-Nov-08	A/goose/Netherlands/21038413-002/2021	Wageningen Bioveterinary Research	Wageningen Bioveterinary Research	Beerens, Nancy; Harders, Frank; Pritz-Verschuren, Sylvia; Roose, Marit; Germeraad, Evelien; Engelsma, Marc; Heutink, Rene
EPI_ISL_6760999	Netherlands	2021-Oct-27	A/black-headed gull/Netherlands/21037590-002/2021	Wageningen Bioveterinary Research	Wageningen Bioveterinary Research	Beerens, Nancy; Harders, Frank; Pritz-Verschuren, Sylvia; Roose, Marit; Germeraad, Evelien; Engelsma, Marc; Heutink, Rene

EPI_ISL_6761000	Netherlands	2021-Oct-31	A/greylag goose/Netherlands/21037809-001/2021	Wageningen Bioveterinary Research	Wageningen Bioveterinary Research	Beerens, Nancy; Harders, Frank; Pritz-Verschuren, Sylvia; Roose, Marit; Germeraad, Evelien; Engelsma, Marc; Heutink, Rene
EPI_ISL_6761001	Netherlands	2021-Nov-05	A/mute swan/Netherlands/21038142-001/2021	Wageningen Bioveterinary Research	Wageningen Bioveterinary Research	Beerens, Nancy; Harders, Frank; Pritz-Verschuren, Sylvia; Roose, Marit; Germeraad, Evelien; Engelsma, Marc; Heutink, Rene
EPI_ISL_6761002	Netherlands	2021-Nov-05	A/mute swan/Netherlands/21038142-004/2021	Wageningen Bioveterinary Research	Wageningen Bioveterinary Research	Beerens, Nancy; Harders, Frank; Pritz-Verschuren, Sylvia; Roose, Marit; Germeraad, Evelien; Engelsma, Marc; Heutink, Rene
EPI_ISL_6761003	Netherlands	2021-Nov-05	A/mute swan/Netherlands/21038142-007/2021	Wageningen Bioveterinary Research	Wageningen Bioveterinary Research	Beerens, Nancy; Harders, Frank; Pritz-Verschuren, Sylvia; Roose, Marit; Germeraad, Evelien; Engelsma, Marc; Heutink, Rene
EPI_ISL_6761004	Netherlands	2021-Nov-01	A/greylag goose/Netherlands/21037883-001/2021	Wageningen Bioveterinary Research	Wageningen Bioveterinary Research	Beerens, Nancy; Harders, Frank; Pritz-Verschuren, Sylvia; Roose, Marit; Germeraad, Evelien; Engelsma, Marc; Heutink, Rene
EPI_ISL_6761005	Netherlands	2021-Nov-05	A/chicken/Netherlands/21038211-001/2021	Wageningen Bioveterinary Research	Wageningen Bioveterinary Research	Beerens, Nancy; Harders, Frank; Pritz-Verschuren, Sylvia; Roose, Marit; Germeraad, Evelien; Engelsma, Marc; Heutink, Rene
EPI_ISL_6761006	Netherlands	2021-Nov-09	A/mute swan/Netherlands/21038362-001/2021	Wageningen Bioveterinary Research	Wageningen Bioveterinary Research	Beerens, Nancy; Harders, Frank; Pritz-Verschuren, Sylvia; Roose, Marit; Germeraad, Evelien; Engelsma, Marc; Heutink, Rene
EPI_ISL_6761007	Netherlands	2021-Nov-09	A/mute swan/Netherlands/21038362-002/2021	Wageningen Bioveterinary Research	Wageningen Bioveterinary Research	Beerens, Nancy; Harders, Frank; Pritz-Verschuren, Sylvia; Roose, Marit; Germeraad, Evelien; Engelsma, Marc; Heutink, Rene
EPI_ISL_6761008	Netherlands	2021-Oct-30	A/greylag goose/Netherlands/21038036-002/2021	Wageningen Bioveterinary Research	Wageningen Bioveterinary Research	Beerens, Nancy; Harders, Frank; Pritz-Verschuren, Sylvia; Roose, Marit; Germeraad, Evelien; Engelsma, Marc; Heutink, Rene
EPI_ISL_6761009	Netherlands	2021-Nov-01	A/greylag goose/Netherlands/21038037-001/2021	Wageningen Bioveterinary Research	Wageningen Bioveterinary Research	Beerens, Nancy; Harders, Frank; Pritz-Verschuren, Sylvia; Roose, Marit; Germeraad, Evelien; Engelsma, Marc; Heutink, Rene
EPI_ISL_6761010	Netherlands	2021-Nov-02	A/mute swan/Netherlands/21038038-001/2021	Wageningen Bioveterinary Research	Wageningen Bioveterinary Research	Beerens, Nancy; Harders, Frank; Pritz-Verschuren, Sylvia; Roose, Marit; Germeraad, Evelien; Engelsma, Marc; Heutink, Rene
EPI_ISL_6761011	Netherlands	2021-Nov-04	A/peregrine falcon/Netherlands/21038169-002/2021	Wageningen Bioveterinary Research	Wageningen Bioveterinary Research	Beerens, Nancy; Harders, Frank; Pritz-Verschuren, Sylvia; Roose, Marit; Germeraad, Evelien; Engelsma, Marc; Heutink, Rene
EPI_ISL_6761012	Netherlands	2021-Nov-02	A/barnacle goose/Netherlands/21038248-001/2021	Wageningen Bioveterinary Research	Wageningen Bioveterinary Research	Beerens, Nancy; Harders, Frank; Pritz-Verschuren, Sylvia; Roose, Marit; Germeraad, Evelien; Engelsma, Marc; Heutink, Rene
EPI_ISL_6761013	Netherlands	2021-Nov-02	A/greylag goose/Netherlands/21038252-001/2021	Wageningen Bioveterinary Research	Wageningen Bioveterinary Research	Beerens, Nancy; Harders, Frank; Pritz-Verschuren, Sylvia; Roose, Marit; Germeraad, Evelien; Engelsma, Marc; Heutink, Rene

EPI_ISL_6761016	Netherlands	2021-Nov-02	A/greylag goose/Netherlands/21038253-001/2021	Wageningen Bioveterinary Research	Wageningen Bioveterinary Research	Beerens, Nancy; Harders, Frank; Pritz-Verschuren, Sylvia; Roose, Marit; Germeraad, Evelien; Engelsma, Marc; Heutink, Rene
EPI_ISL_6761017	Netherlands	2021-Nov-12	A/chicken/Netherlands/21038669-001005/2021	Wageningen Bioveterinary Research	Wageningen Bioveterinary Research	Beerens, Nancy; Harders, Frank; Pritz-Verschuren, Sylvia; Roose, Marit; Germeraad, Evelien; Engelsma, Marc; Heutink, Rene
EPI_ISL_6761018	Netherlands	2021-Nov-11	A/mute swan/Netherlands/21038537-004/2021	Wageningen Bioveterinary Research	Wageningen Bioveterinary Research	Beerens, Nancy; Harders, Frank; Pritz-Verschuren, Sylvia; Roose, Marit; Germeraad, Evelien; Engelsma, Marc; Heutink, Rene
EPI_ISL_6761019	Netherlands	2021-Nov-14	A/mute swan/Netherlands/21038706-002/2021	Wageningen Bioveterinary Research	Wageningen Bioveterinary Research	Beerens, Nancy; Harders, Frank; Pritz-Verschuren, Sylvia; Roose, Marit; Germeraad, Evelien; Engelsma, Marc; Heutink, Rene
EPI_ISL_6761020	Netherlands	2021-Nov-09	A/mute swan/Netherlands/21038479-002/2021	Wageningen Bioveterinary Research	Wageningen Bioveterinary Research	Beerens, Nancy; Harders, Frank; Pritz-Verschuren, Sylvia; Roose, Marit; Germeraad, Evelien; Engelsma, Marc; Heutink, Rene
EPI_ISL_6761021	Netherlands	2021-Nov-07	A/greylag goose/Netherlands/21038565-002/2021	Wageningen Bioveterinary Research	Wageningen Bioveterinary Research	Beerens, Nancy; Harders, Frank; Pritz-Verschuren, Sylvia; Roose, Marit; Germeraad, Evelien; Engelsma, Marc; Heutink, Rene
EPI_ISL_6761022	Netherlands	2021-Nov-09	A/greylag goose/Netherlands/21038567-002/2021	Wageningen Bioveterinary Research	Wageningen Bioveterinary Research	Beerens, Nancy; Harders, Frank; Pritz-Verschuren, Sylvia; Roose, Marit; Germeraad, Evelien; Engelsma, Marc; Heutink, Rene
EPI_ISL_6761023	Netherlands	2021-Nov-13	A/mallard/Netherlands/21038796-002/2021	Wageningen Bioveterinary Research	Wageningen Bioveterinary Research	Beerens, Nancy; Harders, Frank; Pritz-Verschuren, Sylvia; Roose, Marit; Germeraad, Evelien; Engelsma, Marc; Heutink, Rene
EPI_ISL_6761024	Netherlands	2021-Nov-13	A/goose/Netherlands/21038799-002/2021	Wageningen Bioveterinary Research	Wageningen Bioveterinary Research	Beerens, Nancy; Harders, Frank; Pritz-Verschuren, Sylvia; Roose, Marit; Germeraad, Evelien; Engelsma, Marc; Heutink, Rene
EPI_ISL_6761101	Belgium	2021-Nov-04	A/Branta_leucopsis/Belgium/14735_0001/2021	Sciensano - Animal Infectious Diseases	Sciensano, Department of Animal Infectious Diseases	Van Borm, Steven; Roupie, Virginie; Lambrecht, Benedicte; Mathijs, Elisabeth; Steensels, Mieke
EPI_ISL_6929958	Poland	2021-Nov-01	A/turkey/Poland/H1910-T3/2021	National Veterinary Research Institut Poland, PIWet-PIB	National Veterinary Research Institut Poland, PIWet-PIB	E. Swieton, K. Smietanka
EPI_ISL_6929970	Poland	2021-Nov-01	A/turkey/Poland/H1911-N/2021	National Veterinary Research Institut Poland, PIWet-PIB	National Veterinary Research Institut Poland, PIWet-PIB	E. Swieton, K. Smietanka
EPI_ISL_6930238	Poland	2021-Nov-02	A/turkey/Poland/H1913-T1/2021	National Veterinary Research Institut Poland, PIWet-PIB	National Veterinary Research Institut Poland, PIWet-PIB	E. Swieton, K. Smietanka
EPI_ISL_6930564	Poland	2021-Nov-03	A/turkey/Poland/H1924-T1/2021	National Veterinary Research Institut Poland, PIWet-PIB	National Veterinary Research Institut Poland, PIWet-PIB	E. Swieton, K. Smietanka

EPI_ISL_6931008	Poland	2021-Nov-03	A/domestic_goose/Poland/H1931-T1/2021	National Veterinary Research Institut Poland, PIWet-PIB	National Veterinary Research Institut Poland, PIWet-PIB	E. Swieton, K. Smietanka
EPI_ISL_6931288	Poland	2021-Nov-05	A/chicken/Poland/H1940-N/2021	National Veterinary Research Institut Poland, PIWet-PIB	National Veterinary Research Institut Poland, PIWet-PIB	E. Swieton, K. Smietanka
EPI_ISL_6934175	Poland	2021-Nov-07	A/domestic_duck/Poland/H1942-N/2021	National Veterinary Research Institut Poland, PIWet-PIB	National Veterinary Research Institut Poland, PIWet-PIB	E. Swieton, K. Smietanka
EPI_ISL_6935584	Poland	2021-Nov-08	A/turkey/Poland/H1944-N/2021	National Veterinary Research Institut Poland, PIWet-PIB	National Veterinary Research Institut Poland, PIWet-PIB	E. Swieton, K. Smietanka
EPI_ISL_6937114	Poland	2021-Nov-08	A/mute_swan/Poland/MB490-L1/2021	National Veterinary Research Institut Poland, PIWet-PIB	National Veterinary Research Institut Poland, PIWet-PIB	E. Swieton, K. Smietanka
EPI_ISL_7049600	Sweden	2021-Nov-05	A/bean goose/Sweden/SVA211111SZ0372/FB004482/2021	National Veterinary Institute, SVA	National Veterinary Institute	na
EPI_ISL_7050532	Sweden	2021-Nov-08	A/western jackdaw/Sweden/SVA211111SZ0376/FB004483/2021	National Veterinary Institute, SVA	National Veterinary Institute	na
EPI_ISL_7053000	Sweden	2021-Nov-09	A/common buzzard/Sweden/SVA211111SZ0376/FB004484/2021	National Veterinary Institute, SVA	National Veterinary Institute	na
EPI_ISL_7053817	Sweden	2021-Nov-01	A/barnacle goose/Sweden/SVA211111SZ0376/FB004496/2021	National Veterinary Institute, SVA	National Veterinary Institute	na
EPI_ISL_7054529	Sweden	2021-Nov-08	A/greylag goose /Sweden/SVA211111SZ0376/FB004497/M-2021	National Veterinary Institute, SVA	National Veterinary Institute	na
EPI_ISL_7054770	Sweden	2021-Nov-08	A/European herring gull/Sweden/SVA211116SZ0432/FB004518/M-2021	National Veterinary Institute, SVA	National Veterinary Institute	na
EPI_ISL_7055384	Sweden	2021-Nov-17	A/greylag goose /Sweden/SVA211118SZ0354/FB004497/I-2021	National Veterinary Institute, SVA	National Veterinary Institute	na
EPI_ISL_7224437	Czech Republic	2021-Nov-12	A/chicken/Czech Republic/22224-2T/2021	State Veterinary Institute Prague	State Veterinary Institute Prague	Alexander,Nagy;Lenka,Cernikova;Martina,Stara
EPI_ISL_7224454	Czech Republic	2021-Nov-12	A/chicken/Czech Republic/22224-3K/2021	State Veterinary Institute Prague	State Veterinary Institute Prague	Alexander,Nagy;Lenka,Cernikova;Martina,Stara
EPI_ISL_7224463	Czech Republic	2021-Nov-12	A/chicken/Czech Republic/22224-3T/2021	State Veterinary Institute Prague	State Veterinary Institute Prague	Alexander,Nagy;Lenka,Cernikova;Martina,Stara

EPI_ISL_7224472	Czech Republic	2021-Nov-12	A/chicken/Czech Republic/22224-4T/2021	State Veterinary Institute Prague	State Veterinary Institute Prague	Alexander,Nagy;Lenka,Cernikova;Martina,Stara
EPI_ISL_7267243	Netherlands	2021-Nov-09	A/greylag goose/Netherlands/21038570-002/2021	Wageningen Bioveterinary Research	Wageningen Bioveterinary Research	Beerens, Nancy; Harders, Frank; Pritz-Verschuren, Sylvia; Roose, Marit; Venema, Sandra; Germeraad, Evelien; Engelsma, Marc; Heutink, Rene
EPI_ISL_7267244	Netherlands	2021-Nov-12	A/common buzzard/Netherlands/21038793-001/2021	Wageningen Bioveterinary Research	Wageningen Bioveterinary Research	Beerens, Nancy; Harders, Frank; Pritz-Verschuren, Sylvia; Roose, Marit; Venema, Sandra; Germeraad, Evelien; Engelsma, Marc; Heutink, Rene
EPI_ISL_7267245	Netherlands	2021-Nov-13	A/mute swan/Netherlands/21038797-001/2021	Wageningen Bioveterinary Research	Wageningen Bioveterinary Research	Beerens, Nancy; Harders, Frank; Pritz-Verschuren, Sylvia; Roose, Marit; Venema, Sandra; Germeraad, Evelien; Engelsma, Marc; Heutink, Rene
EPI_ISL_7267246	Netherlands	2021-Nov-20	A/mute swan/Netherlands/21039192-001/2021	Wageningen Bioveterinary Research	Wageningen Bioveterinary Research	Beerens, Nancy; Harders, Frank; Pritz-Verschuren, Sylvia; Roose, Marit; Venema, Sandra; Germeraad, Evelien; Engelsma, Marc; Heutink, Rene
EPI_ISL_7267247	Netherlands	2021-Nov-20	A/mute swan/Netherlands/21039202-001/2021	Wageningen Bioveterinary Research	Wageningen Bioveterinary Research	Beerens, Nancy; Harders, Frank; Pritz-Verschuren, Sylvia; Roose, Marit; Venema, Sandra; Germeraad, Evelien; Engelsma, Marc; Heutink, Rene
EPI_ISL_7267248	Netherlands	2021-Nov-14	A/goose/Netherlands/21038940-002/2021	Wageningen Bioveterinary Research	Wageningen Bioveterinary Research	Beerens, Nancy; Harders, Frank; Pritz-Verschuren, Sylvia; Roose, Marit; Venema, Sandra; Germeraad, Evelien; Engelsma, Marc; Heutink, Rene
EPI_ISL_7267249	Netherlands	2021-Nov-15	A/grey heron/Netherlands/21038941-001/2021	Wageningen Bioveterinary Research	Wageningen Bioveterinary Research	Beerens, Nancy; Harders, Frank; Pritz-Verschuren, Sylvia; Roose, Marit; Venema, Sandra; Germeraad, Evelien; Engelsma, Marc; Heutink, Rene
EPI_ISL_7267250	Netherlands	2021-Nov-15	A/greylag goose/Netherlands/21038942-001/2021	Wageningen Bioveterinary Research	Wageningen Bioveterinary Research	Beerens, Nancy; Harders, Frank; Pritz-Verschuren, Sylvia; Roose, Marit; Venema, Sandra; Germeraad, Evelien; Engelsma, Marc; Heutink, Rene
EPI_ISL_7267251	Netherlands	2021-Nov-16	A/greater canada goose /Netherlands/21038971-001/2021	Wageningen Bioveterinary Research	Wageningen Bioveterinary Research	Beerens, Nancy; Harders, Frank; Pritz-Verschuren, Sylvia; Roose, Marit; Venema, Sandra; Germeraad, Evelien; Engelsma, Marc; Heutink, Rene
EPI_ISL_7267252	Netherlands	2021-Nov-17	A/goose/Netherlands/21039029-001/2021	Wageningen Bioveterinary Research	Wageningen Bioveterinary Research	Beerens, Nancy; Harders, Frank; Pritz-Verschuren, Sylvia; Roose, Marit; Venema, Sandra; Germeraad, Evelien; Engelsma, Marc; Heutink, Rene
EPI_ISL_7267253	Netherlands	2021-Nov-18	A/gull/Netherlands/21039180-002/2021	Wageningen Bioveterinary Research	Wageningen Bioveterinary Research	Beerens, Nancy; Harders, Frank; Pritz-Verschuren, Sylvia; Roose, Marit; Venema, Sandra; Germeraad, Evelien; Engelsma, Marc; Heutink, Rene
EPI_ISL_7267254	Netherlands	2021-Nov-23	A/greylag goose/Netherlands/21039376-001/2021	Wageningen Bioveterinary Research	Wageningen Bioveterinary Research	Beerens, Nancy; Harders, Frank; Pritz-Verschuren, Sylvia; Roose, Marit; Venema, Sandra; Germeraad, Evelien; Engelsma, Marc; Heutink, Rene
EPI_ISL_7357590	Croatia	2021-Nov-12	A/mute swan/Croatia/101/2021	Croatian Veterinary Institute, Poultry Centre	Croatian Veterinary Institute	Savić, Vladimir
EPI_ISL_7452805	Sweden	2021-Nov-30	A/Chicken/Sweden/SVA211130SZ0427/FB290424-IP-1/M-2021	National Veterinary Institute, SVA	National Veterinary Institute	na

EPI_ISL_7570462	Croatia	2021-Nov-19	A/mute swan/Croatia/104/2021	Croatian Veterinary Institute, Poultry Centre	Croatian Veterinary Institute	Savić, Vladimir
EPI_ISL_7570634	Croatia	2021-Nov-18	A/goose/Croatia/107/2021	Croatian Veterinary Institute, Poultry Centre	Croatian Veterinary Institute	Savić, Vladimir
EPI_ISL_7626484	Czech Republic	2021-Nov-25	A/chicken/Czech Republic/23404-2K/2021	State Veterinary Institute Prague	State Veterinary Institute Prague	Alexander,Nagy;Lenka,Cernikova;Martina,Stara
EPI_ISL_7626506	Czech Republic	2021-Nov-25	A/chicken/Czech Republic/23404-4K/2021	State Veterinary Institute Prague	State Veterinary Institute Prague	Alexander,Nagy;Lenka,Cernikova;Martina,Stara
EPI_ISL_7626512	Czech Republic	2021-Nov-25	A/chicken/Czech Republic/23404-2T/2021	State Veterinary Institute Prague	State Veterinary Institute Prague	Alexander,Nagy;Lenka,Cernikova;Martina,Stara
EPI_ISL_7626513	Czech Republic	2021-Nov-25	A/chicken/Czech Republic/23404-4T/2021	State Veterinary Institute Prague	State Veterinary Institute Prague	Alexander,Nagy;Lenka,Cernikova;Martina,Stara
EPI_ISL_7626514	Czech Republic	2021-Nov-29	A/duck/Czech Republic/23589-1T/2021	State Veterinary Institute Prague	State Veterinary Institute Prague	Alexander,Nagy;Lenka,Cernikova;Martina,Stara
EPI_ISL_7626526	Czech Republic	2021-Nov-29	A/chicken/Czech Republic/23589-1T/2021	State Veterinary Institute Prague	State Veterinary Institute Prague	Alexander,Nagy;Lenka,Cernikova;Martina,Stara
EPI_ISL_7626532	Czech Republic	2021-Nov-29	A/chicken/Czech Republic/23589-3/2021	State Veterinary Institute Prague	State Veterinary Institute Prague	Alexander,Nagy;Lenka,Cernikova;Martina,Stara
EPI_ISL_7626533	Czech Republic	2021-Nov-29	A/chicken/Czech Republic/23589-4/2021	State Veterinary Institute Prague	State Veterinary Institute Prague	Alexander,Nagy;Lenka,Cernikova;Martina,Stara
EPI_ISL_7635907	Sweden	2021-Nov-26	A/white-tailed eagle/Sweden/SVA211201SZ0380/FB004721/M-2021	National Veterinary Institute, SVA	National Veterinary Institute	na
EPI_ISL_7683077	Netherlands	2021-Nov-24	A/mute swan/Netherlands/21039407-001/2021	Wageningen Bioveterinary Research	Wageningen Bioveterinary Research	Beerens, Nancy; Harders, Frank; Pritz-Verschuren, Sylvia; Roose, Marit; Venema, Sandra; Germeraad, Evelien; Engelsma, Marc; Heutink, Rene
EPI_ISL_7683079	Netherlands	2021-Nov-26	A/mute swan/Netherlands/21039627-002/2021	Wageningen Bioveterinary Research	Wageningen Bioveterinary Research	Beerens, Nancy; Harders, Frank; Pritz-Verschuren, Sylvia; Roose, Marit; Venema, Sandra; Germeraad, Evelien; Engelsma, Marc; Heutink, Rene
EPI_ISL_7683101	Netherlands	2021-Nov-25	A/common buzzard/Netherlands/21039607-002/2021	Wageningen Bioveterinary Research	Wageningen Bioveterinary Research	Beerens, Nancy; Harders, Frank; Pritz-Verschuren, Sylvia; Roose, Marit; Venema, Sandra; Germeraad, Evelien; Engelsma, Marc; Heutink, Rene
EPI_ISL_5403566	Germany	2021-Oct-14	A/Eurasian wigeon/Germany-SH/AI05948/2021	Landeslabor Schleswig-Holstein	Friedrich-Loeffler-Institut	na
EPI_ISL_7733048	Germany	2021-Oct-12	A/black-headed gull/Germany-NI/AI05962/2021	Lebensmittel- und Veterinärinstitut Oldenburg - Standort Veterinärinstitut	Friedrich-Loeffler-Institut	na
EPI_ISL_7748001	Germany	2021-Oct-14	A/Eurasian curlew/Germany-SH/AI05960/2021	Landeslabor Schleswig-Holstein	Friedrich-Loeffler-Institut	na
EPI_ISL_7748188	Germany	2021-Oct-14	A/Eurasian wigeon/Germany-SH/AI05950/2021	Landeslabor Schleswig-Holstein	Friedrich-Loeffler-Institut	na

EPI_ISL_7748188	Germany	2021-Oct-14	A/Eurasian wigeon/Germany-SH/AI05950/2021	Landeslabor Schleswig-Holstein	Friedrich-Loeffler-Institut	na
EPI_ISL_7748262	Germany	2021-Oct-14	A/Eurasian wigeon/Germany-SH/AI05952/2021	Landeslabor Schleswig-Holstein	Friedrich-Loeffler-Institut	na
EPI_ISL_7753130	Germany	2021-Oct-14	A/Eurasian wigeon/Germany-SH/AI05955/2021	Landeslabor Schleswig-Holstein	Friedrich-Loeffler-Institut	na
EPI_ISL_7753152	Germany	2021-Oct-14	A/Eurasian wigeon/Germany-SH/AI05953/2021	Landeslabor Schleswig-Holstein	Friedrich-Loeffler-Institut	na
EPI_ISL_7753173	Germany	2021-Oct-14	A/Eurasian wigeon/Germany-SH/AI05956/2021	Landeslabor Schleswig-Holstein	Friedrich-Loeffler-Institut	na
EPI_ISL_7753193	Germany	2021-Oct-14	A/Eurasian wigeon/Germany-SH/AI05951/2021	Landeslabor Schleswig-Holstein	Friedrich-Loeffler-Institut	na
EPI_ISL_7753211	Germany	2021-Oct-14	A/Eurasian wigeon/Germany-SH/AI05954/2021	Landeslabor Schleswig-Holstein	Friedrich-Loeffler-Institut	na
EPI_ISL_7753230	Germany	2021-Oct-17	A/mallard/Germany-NI/AI06010/2021	Lebensmittel- und Veterinärinstitut Oldenburg - Standort Veterinärinstitut	Friedrich-Loeffler-Institut	na
EPI_ISL_7753251	Germany	2021-Oct-19	A/barnacle goose/Germany-SH/AI06005/2021	Landeslabor Schleswig-Holstein	Friedrich-Loeffler-Institut	na
EPI_ISL_7753272	Germany	2021-Oct-20	A/white-tailed eagle/Germany-MV/AI05975/2021	Landesamt für Landwirtschaft, Lebensmittelsicherheit und Fischerei (LALLF)	Friedrich-Loeffler-Institut	na
EPI_ISL_7753290	Germany	2021-Oct-20	A/turkey/Germany-MV/AI06035/2021	Landesamt für Landwirtschaft, Lebensmittelsicherheit und Fischerei (LALLF)	Friedrich-Loeffler-Institut	na
EPI_ISL_7753309	Germany	2021-Oct-20	A/lesser white-fronted goose/Germany-MV/AI05973/2021	Landesamt für Landwirtschaft, Lebensmittelsicherheit und Fischerei (LALLF)	Friedrich-Loeffler-Institut	na
EPI_ISL_7753329	Germany	2021-Oct-20	A/white stork/Germany-MV/AI05979/2021	Landesamt für Landwirtschaft, Lebensmittelsicherheit und Fischerei (LALLF)	Friedrich-Loeffler-Institut	na
EPI_ISL_7753347	Germany	2021-Oct-21	A/herring gull/Germany-SH/AI06141/2021	Landeslabor Schleswig-Holstein	Friedrich-Loeffler-Institut	na
EPI_ISL_7753367	Germany	2021-Oct-21	A/Eurasian teal/Germany-BY/AI05977/2021	Bayrisches Landesamt für Gesundheit und Lebensmittelsicherheit (LGL)	Friedrich-Loeffler-Institut	na
EPI_ISL_7753387	Germany	2021-Oct-22	A/Eurasian wigeon/Germany-SH/AI06143/2021	Landeslabor Schleswig-Holstein	Friedrich-Loeffler-Institut	na

EPI_ISL_7753396	Germany	2021-Oct-22	A/domestic goose/Germany-SH/AI06147/2021	Landeslabor Schleswig-Holstein	Friedrich-Loeffler-Institut	na
EPI_ISL_7753399	Germany	2021-Oct-22	A/domestic goose/Germany-SH/AI06150/2021	Landeslabor Schleswig-Holstein	Friedrich-Loeffler-Institut	na
EPI_ISL_7753400	Germany	2021-Oct-22	A/domestic goose/Germany-SH/AI06024/2021	Landeslabor Schleswig-Holstein	Friedrich-Loeffler-Institut	na
EPI_ISL_7753405	Germany	2021-Oct-22	A/Eurasian wigeon/Germany-SH/AI06142/2021	Landeslabor Schleswig-Holstein	Friedrich-Loeffler-Institut	na
EPI_ISL_7753406	Germany	2021-Oct-25	A/greylag goose/Germany-SH/AI06144/2021	Landeslabor Schleswig-Holstein	Friedrich-Loeffler-Institut	na
EPI_ISL_7753407	Germany	2021-Oct-25	A/barnacle goose/Germany-SH/AI06145/2021	Landeslabor Schleswig-Holstein	Friedrich-Loeffler-Institut	na
EPI_ISL_7753425	Germany	2021-Oct-25	A/greylag goose/Germany-SH/AI06205/2021	Landeslabor Schleswig-Holstein	Friedrich-Loeffler-Institut	na
EPI_ISL_7753443	Germany	2021-Oct-26	A/buzzard/Germany-SH/AI06210/2021	Landeslabor Schleswig-Holstein	Friedrich-Loeffler-Institut	na
EPI_ISL_7753387	Germany	2021-Oct-22	A/Eurasian wigeon/Germany-SH/AI06143/2021	Landeslabor Schleswig-Holstein	Friedrich-Loeffler-Institut	na
EPI_ISL_7778754	Estonia	2021-Oct-12	A/Withe-tiled_eagle/Estonia/TA2124126-1_21VIR10433-11/2021	Estonian Veterinary and Food Laboratory	Istituto Zooprofilattico Sperimentale Delle Venezie	Nurmoja, I.; Vilem, A.; Juurik, T.; Zecchin, B.; Fusaro, A.; Schivo, A.; Salviato, A.; Palumbo, E.; Milani, A.; Giussani, E.; Pastori, A.; Monne, I.; Terregino, C.
EPI_ISL_7880689	Belgium	2021-Dec-07	A/Gallus_gallus/Belgium/16070_003/2021	Sciensano - Animal Infectious Diseases	Sciensano, Department of Animal Infectious Diseases	Van Borm, Steven; Roupie, Virginie; Lambrecht, Benedicte; Mathijs, Elisabeth; Steensels, Mieke
EPI_ISL_7880696	Belgium	2021-Dec-06	A/Gallus_gallus/Belgium/15977/2021	Sciensano - Animal Infectious Diseases	Sciensano, Department of Animal Infectious Diseases	Van Borm, Steven; Roupie, Virginie; Lambrecht, Benedicte; Mathijs, Elisabeth; Steensels, Mieke
EPI_ISL_7892492	Croatia	2021-Dec-08	A/grey heron/Croatia/132/2021	Croatian Veterinary Institute, Poultry Centre	Croatian Veterinary Institute	Savić, Vladimir
EPI_ISL_7952115	Netherlands	2021-Nov-24	A/mute swan/Netherlands/21039526-002/2021	Wageningen Bioveterinary Research	Wageningen Bioveterinary Research	Beerens, Nancy; Harders, Frank; Pritz-Verschuren, Sylvia; Roose, Marit; Venema, Sandra; Germeraad, Evelien; Engelsma, Marc; Heutink, Rene
EPI_ISL_7952116	Netherlands	2021-Dec-01	A/chicken/Netherlands/21039901-001-005/2021	Wageningen Bioveterinary Research	Wageningen Bioveterinary Research	Beerens, Nancy; Harders, Frank; Pritz-Verschuren, Sylvia; Roose, Marit; Venema, Sandra; Germeraad, Evelien; Engelsma, Marc; Heutink, Rene
EPI_ISL_7952117	Netherlands	2021-Dec-16	A/chicken/Netherlands/21040808-001-005/2021	Wageningen Bioveterinary Research	Wageningen Bioveterinary Research	Beerens, Nancy; Harders, Frank; Pritz-Verschuren, Sylvia; Roose, Marit; Venema, Sandra; Germeraad, Evelien; Engelsma, Marc; Heutink, Rene
EPI_ISL_7952118	Netherlands	2021-Dec-16	A/chicken/Netherlands/21040811-001-005/2021	Wageningen Bioveterinary Research	Wageningen Bioveterinary Research	Beerens, Nancy; Harders, Frank; Pritz-Verschuren, Sylvia; Roose, Marit; Venema, Sandra; Germeraad, Evelien; Engelsma, Marc; Heutink, Rene
EPI_ISL_7952119	Netherlands	2021-Nov-28	A/great black-backed gull/Netherlands/21039609-001-002/2021	Wageningen Bioveterinary Research	Wageningen Bioveterinary Research	Beerens, Nancy; Harders, Frank; Pritz-Verschuren, Sylvia; Roose, Marit; Venema, Sandra; Germeraad, Evelien; Engelsma, Marc; Heutink, Rene

EPI_ISL_7952120	Netherlands	2021-Nov-24	A/greylag goose/Netherlands/21039525-001/2021	Wageningen Bioveterinary Research	Wageningen Bioveterinary Research	Beerens, Nancy; Harders, Frank; Pritz-Verschuren, Sylvia; Roose, Marit; Venema, Sandra; Germeraad, Evelien; Engelsma, Marc; Heutink, Rene
EPI_ISL_7952121	Netherlands	2021-Nov-22	A/sanderling/Netherlands/21039528-002/2021	Wageningen Bioveterinary Research	Wageningen Bioveterinary Research	Beerens, Nancy; Harders, Frank; Pritz-Verschuren, Sylvia; Roose, Marit; Venema, Sandra; Germeraad, Evelien; Engelsma, Marc; Heutink, Rene
EPI_ISL_7952122	Netherlands	2021-Nov-26	A/greylag goose/Netherlands/21039746-002/2021	Wageningen Bioveterinary Research	Wageningen Bioveterinary Research	Beerens, Nancy; Harders, Frank; Pritz-Verschuren, Sylvia; Roose, Marit; Venema, Sandra; Germeraad, Evelien; Engelsma, Marc; Heutink, Rene
EPI_ISL_7952123	Netherlands	2021-Nov-25	A/barnacle goose/Netherlands/21039750-002/2021	Wageningen Bioveterinary Research	Wageningen Bioveterinary Research	Beerens, Nancy; Harders, Frank; Pritz-Verschuren, Sylvia; Roose, Marit; Venema, Sandra; Germeraad, Evelien; Engelsma, Marc; Heutink, Rene
EPI_ISL_7952124	Netherlands	2021-Nov-30	A/mute swan/Netherlands/21039841-002/2021	Wageningen Bioveterinary Research	Wageningen Bioveterinary Research	Beerens, Nancy; Harders, Frank; Pritz-Verschuren, Sylvia; Roose, Marit; Venema, Sandra; Germeraad, Evelien; Engelsma, Marc; Heutink, Rene
EPI_ISL_7952125	Netherlands	2021-Nov-25	A/mute swan/Netherlands/21039824-002/2021	Wageningen Bioveterinary Research	Wageningen Bioveterinary Research	Beerens, Nancy; Harders, Frank; Pritz-Verschuren, Sylvia; Roose, Marit; Venema, Sandra; Germeraad, Evelien; Engelsma, Marc; Heutink, Rene
EPI_ISL_7952126	Netherlands	2021-Nov-07	A/greylag goose/Netherlands/21038417-001/2021	Wageningen Bioveterinary Research	Wageningen Bioveterinary Research	Beerens, Nancy; Harders, Frank; Pritz-Verschuren, Sylvia; Roose, Marit; Venema, Sandra; Germeraad, Evelien; Engelsma, Marc; Heutink, Rene
EPI_ISL_7952127	Netherlands	2021-Nov-23	A/goose/Netherlands/21039364-002/2021	Wageningen Bioveterinary Research	Wageningen Bioveterinary Research	Beerens, Nancy; Harders, Frank; Pritz-Verschuren, Sylvia; Roose, Marit; Venema, Sandra; Germeraad, Evelien; Engelsma, Marc; Heutink, Rene
EPI_ISL_7952128	Netherlands	2021-Nov-09	A/gadwall/Netherlands/21038566-002/2021	Wageningen Bioveterinary Research	Wageningen Bioveterinary Research	Beerens, Nancy; Harders, Frank; Pritz-Verschuren, Sylvia; Roose, Marit; Venema, Sandra; Germeraad, Evelien; Engelsma, Marc; Heutink, Rene
EPI_ISL_7952129	Netherlands	2021-Nov-20	A/western jackdaw/Netherlands/21039297-002/2021	Wageningen Bioveterinary Research	Wageningen Bioveterinary Research	Beerens, Nancy; Harders, Frank; Pritz-Verschuren, Sylvia; Roose, Marit; Venema, Sandra; Germeraad, Evelien; Engelsma, Marc; Heutink, Rene
EPI_ISL_7996368	Netherlands	2021-Nov-07	A/mute swan/Netherlands/21038568-005/2021	Wageningen Bioveterinary Research	Wageningen Bioveterinary Research	Beerens, Nancy; Harders, Frank; Pritz-Verschuren, Sylvia; Roose, Marit; Venema, Sandra; Germeraad, Evelien; Engelsma, Marc; Heutink, Rene
EPI_ISL_7996369	Netherlands	2021-Nov-19	A/mute swan/Netherlands/21039291-002/2021	Wageningen Bioveterinary Research	Wageningen Bioveterinary Research	Beerens, Nancy; Harders, Frank; Pritz-Verschuren, Sylvia; Roose, Marit; Venema, Sandra; Germeraad, Evelien; Engelsma, Marc; Heutink, Rene
EPI_ISL_7996370	Netherlands	2021-Nov-20	A/western jackdaw/Netherlands/21039294-002/2021	Wageningen Bioveterinary Research	Wageningen Bioveterinary Research	Beerens, Nancy; Harders, Frank; Pritz-Verschuren, Sylvia; Roose, Marit; Venema, Sandra; Germeraad, Evelien; Engelsma, Marc; Heutink, Rene
EPI_ISL_7996371	Netherlands	2021-Dec-20	A/turkey/Netherlands/21040980-001005/2021	Wageningen Bioveterinary Research	Wageningen Bioveterinary Research	Beerens, Nancy; Harders, Frank; Pritz-Verschuren, Sylvia; Roose, Marit; Venema, Sandra; Germeraad, Evelien; Engelsma, Marc; Heutink, Rene

EPI_ISL_8338002	Sweden	2021-Dec-12	A/Turkey/Sweden/SVA211212SZ0001/FB301013-IP-2/M-2021	National Veterinary Institute, SVA	National Veterinary Institute	'Siamak, Zohari'
EPI_ISL_8338456	Croatia	2021-Dec-27	A/mute swan/Croatia/144/2021	Croatian Veterinary Institute, Poultry Centre	Croatian Veterinary Institute	Savić, Vladimir
EPI_ISL_8338502	Croatia	2021-Dec-29	A/mute swan/Croatia/145/2021	Croatian Veterinary Institute, Poultry Centre	Croatian Veterinary Institute	Savić, Vladimir
EPI_ISL_8377056	France	2021-Dec-15	A/pelican/France/21P013720/2021	Anses (Ploufragan-Plouzané)	ANSES Agence Nationale De Securite Sanitaire De L'alimentation	na
EPI_ISL_8377254	France	2021-Dec-03	A/egret/France/21P013418/2021	Anses (Ploufragan-Plouzané)	ANSES Agence Nationale De Securite Sanitaire De L'alimentation	na
EPI_ISL_8377417	France	2021-Nov-25	A/chicken/France/21P013076/2021	Anses (Ploufragan-Plouzané)	ANSES Agence Nationale De Securite Sanitaire De L'alimentation	na
EPI_ISL_8377418	France	2021-Nov-25	A/goose/France/21P013228/2021	Anses (Ploufragan-Plouzané)	ANSES Agence Nationale De Securite Sanitaire De L'alimentation	na
EPI_ISL_8416806	Portugal	2021-Dec-05	A/turkey/Lisbon/1/2021	Instituto Nacional de Investigação Agrária e Veterinária, I. P.	Instituto Nacional de Saúde Dr. Ricardo Jorge	Ana Margarida, Henriques; Teresa, Fagulha; Fernanda, Ramos; Margarida, Duarte; Raquel, Guiomar; Aryse, Melo; Inês, Costa; Patricia, Conde; Nuno, Verdasca; Camila, Henriques
EPI_ISL_8439476	Croatia	2021-Dec-29	A/mute swan/Croatia/146/2021	Croatian Veterinary Institute, Poultry Centre	Croatian Veterinary Institute	Savić, Vladimir
EPI_ISL_8515478	Czech Republic	2021-Nov-18	A/goose/Czech_Republic/22608-1/2021	State Veterinary Institute Prague	State Veterinary Institute Prague	Alexander,Nagy;Lenka,Cernikova;Martina,Stara
EPI_ISL_8515479	Czech Republic	2021-Nov-18	A/goose/Czech_Republic/22608-2/2021	State Veterinary Institute Prague	State Veterinary Institute Prague	Alexander,Nagy;Lenka,Cernikova;Martina,Stara
EPI_ISL_8515480	Czech Republic	2021-Nov-25	A/chicken/Czech_Republic/23404/2021	State Veterinary Institute Prague	State Veterinary Institute Prague	Alexander,Nagy;Lenka,Cernikova;Martina,Stara
EPI_ISL_8515481	Czech Republic	2021-Nov-28	A/grey heron/Czech_Republic/23608/2021	State Veterinary Institute Prague	State Veterinary Institute Prague	Alexander,Nagy;Lenka,Cernikova;Martina,Stara
EPI_ISL_8515482	Czech Republic	2021-Nov-28	A/grey heron/Czech_Republic/23608-1K/2021	State Veterinary Institute Prague	State Veterinary Institute Prague	na
EPI_ISL_8515483	Czech Republic	2021-Nov-28	A/great egret/Czech_Republic/23609/2021	State Veterinary Institute Prague	State Veterinary Institute Prague	Alexander,Nagy;Lenka,Cernikova;Martina,Stara
EPI_ISL_8568483	Croatia	2021-Nov-22	A/gadwall/Croatia/108/2021	Croatian Veterinary Institute, Poultry Centre	Croatian Veterinary Institute	Savić, Vladimir

EPI_ISL_8650949	Netherlands	2021-Dec-20	A/red knot/Netherlands/21041030-008/2021	Wageningen Bioveterinary Research	Wageningen Bioveterinary Research	Beerens, Nancy; Harders, Frank; Pritz-Verschuren, Sylvia; Roose, Marit; Venema, Sandra; Germeraad, Evelien; Engelsma, Marc; Heutink, Rene
EPI_ISL_8694562	Croatia	2022-Jan-06	A/mute swan/Croatia/6/2022	Croatian Veterinary Institute, Poultry Centre	Croatian Veterinary Institute	Savić, Vladimir
EPI_ISL_8694715	Croatia	2022-Jan-10	A/chicken/Croatia/7/2022	Croatian Veterinary Institute, Poultry Centre	Croatian Veterinary Institute	Savić, Vladimir
EPI_ISL_8768967	Russian Federation	2021-Oct-02	A/chicken/Tyumen/27-31V/2021	State Research Center of Virology and Biotechnology (VECTOR)	State Research Center of Virology and Biotechnology (VECTOR)	na
EPI_ISL_8768968	Russian Federation	2021-Oct-02	A/chicken/Tyumen/27-39V/2021	State Research Center of Virology and Biotechnology (VECTOR)	State Research Center of Virology and Biotechnology (VECTOR)	na
EPI_ISL_8768974	Russian Federation	2021-Oct-02	A/chicken/Tyumen/27-42V/2021	State Research Center of Virology and Biotechnology (VECTOR)	State Research Center of Virology and Biotechnology (VECTOR)	na
EPI_ISL_8768986	Russian Federation	2021-Oct-10	A/goose/Rostov-on-Don/28-1V/2021	State Research Center of Virology and Biotechnology (VECTOR)	State Research Center of Virology and Biotechnology (VECTOR)	na
EPI_ISL_8769007	Russian Federation	2021-Oct-07	A/chicken/Tyumen/33-45V/2021	State Research Center of Virology and Biotechnology (VECTOR)	State Research Center of Virology and Biotechnology (VECTOR)	na
EPI_ISL_8769008	Russian Federation	2021-Oct-07	A/goose/Tyumen/33-52V/2021	State Research Center of Virology and Biotechnology (VECTOR)	State Research Center of Virology and Biotechnology (VECTOR)	na
EPI_ISL_8769009	Russian Federation	2021-Oct-07	A/goose/Tyumen/33-53V/2021	State Research Center of Virology and Biotechnology (VECTOR)	State Research Center of Virology and Biotechnology (VECTOR)	na
EPI_ISL_8769010	Russian Federation	2021-Oct-11	A/goose/Chelyabinsk/34-1V/2021	State Research Center of Virology and Biotechnology (VECTOR)	State Research Center of Virology and Biotechnology (VECTOR)	na
EPI_ISL_8769012	Russian Federation	2021-Oct-15	A/chicken/Orenburg/46-1V/2021	Center of Hygiene and Epidemiology in Orenburg Oblast	State Research Center of Virology and Biotechnology (VECTOR)	na
EPI_ISL_8769013	Russian Federation	2021-Oct-15	A/chicken/Orenburg/46-1V/2021	State Research Center of Virology and Biotechnology (VECTOR)	State Research Center of Virology and Biotechnology (VECTOR)	na
EPI_ISL_8769014	Russian Federation	2021-Oct-15	A/chicken/Orenburg/46-2V/2021	Center of Hygiene and Epidemiology in Orenburg Oblast	State Research Center of Virology and Biotechnology (VECTOR)	na
EPI_ISL_8769015	Russian Federation	2021-Oct-15	A/chicken/Orenburg/46-2V/2021	State Research Center of Virology and Biotechnology (VECTOR)	State Research Center of Virology and Biotechnology (VECTOR)	na

EPI_ISL_8769016	Russian Federation	2021-Oct-15	A/goose/Orenburg/46-3V/2021	State Research Center of Virology and Biotechnology (VECTOR)	State Research Center of Virology and Biotechnology (VECTOR)	na
EPI_ISL_8769017	Russian Federation	2021-Oct-11	A/chicken/Tyumen/47-66V/2021	FBUZ Center of Hygiene and Epidemiology in Tyumen oblast	State Research Center of Virology and Biotechnology (VECTOR)	na
EPI_ISL_8769018	Russian Federation	2021-Oct-12	A/chicken/Tyumen/47-79V/2021	State Research Center of Virology and Biotechnology (VECTOR)	State Research Center of Virology and Biotechnology (VECTOR)	na
EPI_ISL_8769019	Russian Federation	2021-Oct-12	A/chicken/Tyumen/47-85V/2021	FBUZ Center of Hygiene and Epidemiology in Tyumen oblast	State Research Center of Virology and Biotechnology (VECTOR)	na
EPI_ISL_8769020	Russian Federation	2021-Oct-12	A/chicken/Tyumen/47-85V/2021	State Research Center of Virology and Biotechnology (VECTOR)	State Research Center of Virology and Biotechnology (VECTOR)	na
EPI_ISL_8769021	Russian Federation	2021-Oct-12	A/chicken/Tyumen/47-88V/2021	State Research Center of Virology and Biotechnology (VECTOR)	State Research Center of Virology and Biotechnology (VECTOR)	na
EPI_ISL_8769022	Russian Federation	2021-Oct-12	A/chicken/Tyumen/47-95V/2021	FBUZ Center of Hygiene and Epidemiology in Tyumen oblast	State Research Center of Virology and Biotechnology (VECTOR)	na
EPI_ISL_8769023	Russian Federation	2021-Oct-27	A/chicken/Kirov/63-1V/2021	State Research Center of Virology and Biotechnology (VECTOR)	State Research Center of Virology and Biotechnology (VECTOR)	na
EPI_ISL_8769024	Russian Federation	2021-Oct-27	A/chicken/Kirov/63-2V/2021	State Research Center of Virology and Biotechnology (VECTOR)	State Research Center of Virology and Biotechnology (VECTOR)	na
EPI_ISL_8769025	Russian Federation	2021-Oct-27	A/chicken/Kirov/63-5V/2021	State Research Center of Virology and Biotechnology (VECTOR)	State Research Center of Virology and Biotechnology (VECTOR)	na
EPI_ISL_8769026	Russian Federation	2021-Oct-22	A/chicken/Kurgan/72-1V/2021	Center of Hygiene and Epidemiology in Kurgan Oblast	State Research Center of Virology and Biotechnology (VECTOR)	na
EPI_ISL_8769027	Russian Federation	2021-Oct-22	A/chicken/Kurgan/72-1V/2021	State Research Center of Virology and Biotechnology (VECTOR)	State Research Center of Virology and Biotechnology (VECTOR)	na
EPI_ISL_8769028	Russian Federation	2021-Oct-27	A/chicken/Orenburg/73-5V/2021	Center of Hygiene and Epidemiology in Orenburg Oblast	State Research Center of Virology and Biotechnology (VECTOR)	na
EPI_ISL_8769029	Russian Federation	2021-Oct-27	A/chicken/Orenburg/73-6V/2021	State Research Center of Virology and Biotechnology (VECTOR)	State Research Center of Virology and Biotechnology (VECTOR)	na

EPI_ISL_8769030	Russian Federation	2021-Oct-27	A/chicken/Orenburg/73-7V/2021	Center of Hygiene and Epidemiology in Orenburg Oblast	State Research Center of Virology and Biotechnology (VECTOR)	na
EPI_ISL_8769033	Russian Federation	2021-Oct-30	A/turkey/Tyumen/81-96V/2021	State Research Center of Virology and Biotechnology (VECTOR)	State Research Center of Virology and Biotechnology (VECTOR)	na
EPI_ISL_8769034	Russian Federation	2021-Oct-30	A/chicken/Tyumen/81-97V/2021	FBUZ Center of Hygiene and Epidemiology in Tyumen oblast	State Research Center of Virology and Biotechnology (VECTOR)	na
EPI_ISL_8769036	Russian Federation	2021-Oct-13	A/chicken/Saratov/102-12V/2021	Center of Hygiene and Epidemiology in Saratov Oblast	State Research Center of Virology and Biotechnology (VECTOR)	na
EPI_ISL_8769037	Russian Federation	2021-Nov-04	A/chicken/Saratov/102-12V/2021	State Research Center of Virology and Biotechnology (VECTOR)	State Research Center of Virology and Biotechnology (VECTOR)	na
EPI_ISL_8769038	Russian Federation	2021-Nov-04	A/chicken/Saratov/102-15V/2021	State Research Center of Virology and Biotechnology (VECTOR)	State Research Center of Virology and Biotechnology (VECTOR)	na
EPI_ISL_8799314	Netherlands	2021-Dec-17	A/red knot/Netherlands/21040956-003/2021	Wageningen Bioveterinary Research	Wageningen Bioveterinary Research	Beerens, Nancy; Harders, Frank; Pritz-Verschuren, Sylvia; Roose, Marit; Venema, Sandra; Germeraad, Evelien; Engelsma, Marc; Heutink, Rene
EPI_ISL_8799315	Netherlands	2021-Dec-22	A/mallard/Netherlands/21041322-001/2021	Wageningen Bioveterinary Research	Wageningen Bioveterinary Research	Beerens, Nancy; Harders, Frank; Pritz-Verschuren, Sylvia; Roose, Marit; Venema, Sandra; Germeraad, Evelien; Engelsma, Marc; Heutink, Rene
EPI_ISL_8799317	Netherlands	2021-Dec-24	A/seagull/Netherlands/21041370-001/2021	Wageningen Bioveterinary Research	Wageningen Bioveterinary Research	Beerens, Nancy; Harders, Frank; Pritz-Verschuren, Sylvia; Roose, Marit; Venema, Sandra; Germeraad, Evelien; Engelsma, Marc; Heutink, Rene
EPI_ISL_8799318	Netherlands	2021-Dec-26	A/common buzzard/Netherlands/21041449-002/2021	Wageningen Bioveterinary Research	Wageningen Bioveterinary Research	Beerens, Nancy; Harders, Frank; Pritz-Verschuren, Sylvia; Roose, Marit; Venema, Sandra; Germeraad, Evelien; Engelsma, Marc; Heutink, Rene
EPI_ISL_8799552	United Kingdom	2021-Dec-26	A/England/215201407/2021	UK Health Security Agency - Colindale	UK Health Security Agency (UKHSA)	UKHSA, Respiratory Virus Unit
EPI_ISL_8809153	United Kingdom	2021-Dec-21	A/Muscovy duck/England/074477/2021	Animal and Plant Health Agency (APHA)	Animal and Plant Health Agency (APHA)	na
EPI_ISL_8814146	United Kingdom	2021-Nov-06	A/turkey/England/055251/2021	Animal and Plant Health Agency (APHA)	Animal and Plant Health Agency (APHA)	na
EPI_ISL_8814195	United Kingdom	2021-Nov-10	A/turkey/England/056764/2021	Animal and Plant Health Agency (APHA)	Animal and Plant Health Agency (APHA)	na
EPI_ISL_9009291	Russian Federation	2021-Nov-27	A/chicken/Kursk/132-1V/2021	Center of Hygiene and Epidemiology in Kursk Oblast	State Research Center of Virology and Biotechnology (VECTOR)	na

EPI_ISL_9009292	Russian Federation	2021-Nov-27	A/chicken/Kursk/132-1V/2021	State Research Center of Virology and Biotechnology (VECTOR)	State Research Center of Virology and Biotechnology (VECTOR)	na
EPI_ISL_9009294	Russian Federation	2021-Dec-02	A/chicken/Stavropol/146-1V/2021	State Research Center of Virology and Biotechnology (VECTOR)	State Research Center of Virology and Biotechnology (VECTOR)	na
EPI_ISL_9009296	Russian Federation	2021-Dec-13	A/chicken/Rostov-on-Don/159-1V/2021	State Research Center of Virology and Biotechnology (VECTOR)	State Research Center of Virology and Biotechnology (VECTOR)	na
EPI_ISL_9009297	Russian Federation	2021-Dec-13	A/chicken/Rostov-on-Don/159-2V/2021	State Research Center of Virology and Biotechnology (VECTOR)	State Research Center of Virology and Biotechnology (VECTOR)	na
EPI_ISL_9009302	Russian Federation	2021-Dec-13	A/chicken/Rostov-on-Don/159-3V/2021	State Research Center of Virology and Biotechnology (VECTOR)	State Research Center of Virology and Biotechnology (VECTOR)	na
EPI_ISL_9009303	Russian Federation	2021-Dec-13	A/chicken/Rostov-on-Don/159-4V/2021	State Research Center of Virology and Biotechnology (VECTOR)	State Research Center of Virology and Biotechnology (VECTOR)	na
EPI_ISL_9009304	Russian Federation	2021-Dec-13	A/chicken/Rostov-on-Don/159-5V/2021	State Research Center of Virology and Biotechnology (VECTOR)	State Research Center of Virology and Biotechnology (VECTOR)	na
EPI_ISL_9009309	Russian Federation	2021-Dec-13	A/chicken/Rostov-on-Don/159-6V/2021	State Research Center of Virology and Biotechnology (VECTOR)	State Research Center of Virology and Biotechnology (VECTOR)	na
EPI_ISL_9009317	Russian Federation	2021-Dec-13	A/chicken/Rostov-on-Don/159-7V/2021	State Research Center of Virology and Biotechnology (VECTOR)	State Research Center of Virology and Biotechnology (VECTOR)	na
EPI_ISL_9012457	United Kingdom	2021-Oct-24	A/chicken/England/053052/2021	Animal and Plant Health Agency (APHA)	Animal and Plant Health Agency (APHA)	na
EPI_ISL_9012572	United Kingdom	2021-Oct-27	A/pheasant/Wales/385129/2021	Animal and Plant Health Agency (APHA)	Animal and Plant Health Agency (APHA)	na
EPI_ISL_9012574	Netherlands	2021-Dec-17	A/Herring Gull/Netherlands/1/2021	Erasmus Medical Center	Erasmus Medical Center	na
EPI_ISL_9012577	Netherlands	2021-Dec-27	A/Mallard/Netherlands/13/2021	Erasmus Medical Center	Erasmus Medical Center	na
EPI_ISL_9012578	Netherlands	2021-Dec-27	A/Mallard/Netherlands/14/2021	Erasmus Medical Center	Erasmus Medical Center	na
EPI_ISL_9012580	Netherlands	2021-Dec-27	A/Mallard/Netherlands/15/2021	Erasmus Medical Center	Erasmus Medical Center	na
EPI_ISL_9012618	United Kingdom	2021-Oct-30	A/chicken/Wales/053969/2021	Animal and Plant Health Agency (APHA)	Animal and Plant Health Agency (APHA)	na
EPI_ISL_9012694	United Kingdom	2021-Nov-01	A/guineafowl/Scotland/054471/2021	Animal and Plant Health Agency (APHA)	Animal and Plant Health Agency (APHA)	na

EPI_ISL_9012696	United Kingdom	2021-Nov-01	A/chicken/Scotland/054477/2021	Animal and Plant Health Agency (APHA)	Animal and Plant Health Agency (APHA)	na
EPI_ISL_9012700	United Kingdom	2021-Nov-01	A/domestic_duck/Scotland/054469/2021	Animal and Plant Health Agency (APHA)	Animal and Plant Health Agency (APHA)	na
EPI_ISL_9029956	United Kingdom	2021-Nov-14	A/chicken/England/057314/2021	Animal and Plant Health Agency (APHA)	Animal and Plant Health Agency (APHA)	na
EPI_ISL_9029957	United Kingdom	2021-Nov-16	A/turkey/England/057679/2021	Animal and Plant Health Agency (APHA)	Animal and Plant Health Agency (APHA)	na
EPI_ISL_9029959	United Kingdom	2021-Nov-18	A/domestic_duck/England/058612/2021	Animal and Plant Health Agency (APHA)	Animal and Plant Health Agency (APHA)	na
EPI_ISL_9029960	United Kingdom	2021-Nov-11	A/mute_swan/England/385466/2021	Animal and Plant Health Agency (APHA)	Animal and Plant Health Agency (APHA)	na
EPI_ISL_9029961	United Kingdom	2021-Nov-01	A/Canada_goose/England/385250/2021	Animal and Plant Health Agency (APHA)	Animal and Plant Health Agency (APHA)	na
EPI_ISL_9029962	United Kingdom	2021-Nov-09	A/Whooper_swan/Scotland/056219/2021	Animal and Plant Health Agency (APHA)	Animal and Plant Health Agency (APHA)	na
EPI_ISL_9029965	United Kingdom	2021-Oct-30	A/Greylag_goose/England/054503/2021	Animal and Plant Health Agency (APHA)	Animal and Plant Health Agency (APHA)	na
EPI_ISL_9111105	Netherlands	2022-Jan-22	A/chicken/Netherlands/22001401-001005/2022	Wageningen Bioveterinary Research	Wageningen Bioveterinary Research	Beerens, Nancy; Harders, Frank; Pritz-Verschuren, Sylvia; Roose, Marit; Venema, Sandra; Germeraad, Evelien; Engelsma, Marc; Heutink, Rene
EPI_ISL_9117228	Netherlands	2022-Jan-04	A/northern goshawk/Netherlands/22000305-002/2022	Wageningen Bioveterinary Research	Wageningen Bioveterinary Research	Beerens, Nancy; Harders, Frank; Pritz-Verschuren, Sylvia; Roose, Marit; Venema, Sandra; Germeraad, Evelien; Engelsma, Marc; Heutink, Rene
EPI_ISL_9160206	Belgium	2021-Nov-21	A/Anser_albifrons/Belgium/15465_0010/2021	Sciensano - Animal Infectious Diseases	Sciensano, Department of Animal Infectious Diseases	Van Borm, Steven; Roupie, Virginie; Lambrecht, Benedicte; Mathijs, Elisabeth; Steensels, Mieke
EPI_ISL_9161618	Belgium	2021-Dec-27	A/Gallus_gallus/Belgium/17100_0001/2021	Sciensano - Animal Infectious Diseases	Sciensano, Department of Animal Infectious Diseases	Van Borm, Steven; Roupie, Virginie; Lambrecht, Benedicte; Mathijs, Elisabeth; Steensels, Mieke
EPI_ISL_9162331	Belgium	2022-Jan-04	A/Phasianus_colchicus/Belgium/294/2022	Sciensano - Animal Infectious Diseases	Sciensano, Department of Animal Infectious Diseases	Van Borm, Steven; Roupie, Virginie; Lambrecht, Benedicte; Mathijs, Elisabeth; Steensels, Mieke
EPI_ISL_9221411	Netherlands	2021-Dec-27	A/barnacle goose/Netherlands/21041450-001/2021	Wageningen Bioveterinary Research	Wageningen Bioveterinary Research	Beerens, Nancy; Harders, Frank; Pritz-Verschuren, Sylvia; Roose, Marit; Venema, Sandra; Germeraad, Evelien; Engelsma, Marc; Heutink, Rene
EPI_ISL_9221430	Netherlands	2021-Dec-27	A/common buzzard/Netherlands/21041451-002/2021	Wageningen Bioveterinary Research	Wageningen Bioveterinary Research	Beerens, Nancy; Harders, Frank; Pritz-Verschuren, Sylvia; Roose, Marit; Venema, Sandra; Germeraad, Evelien; Engelsma, Marc; Heutink, Rene
EPI_ISL_9221469	Netherlands	2021-Dec-25	A/lesser black-backed gull/Netherlands/21041570-001/2021	Wageningen Bioveterinary Research	Wageningen Bioveterinary Research	Beerens, Nancy; Harders, Frank; Pritz-Verschuren, Sylvia; Roose, Marit; Venema, Sandra; Germeraad, Evelien; Engelsma, Marc; Heutink, Rene
EPI_ISL_9221519	Netherlands	2022-Jan-06	A/barnacle goose/Netherlands/22000419-002/2022	Wageningen Bioveterinary Research	Wageningen Bioveterinary Research	Beerens, Nancy; Harders, Frank; Pritz-Verschuren, Sylvia; Roose, Marit; Venema, Sandra; Germeraad, Evelien; Engelsma, Marc; Heutink, Rene

EPI_ISL_9261741	Netherlands	2022-Jan-04	A/Barnacle goose/Netherlands/1/2022	Erasmus Medical Center	Erasmus Medical Center	na
EPI_ISL_9261742	Netherlands	2022-Jan-06	A/Great black-backed gull/1/2022	Erasmus Medical Center	Erasmus Medical Center	na
EPI_ISL_9261743	Netherlands	2022-Jan-11	A/Oystercatcher/Netherlands/1/2022	Erasmus Medical Center	Erasmus Medical Center	na
EPI_ISL_9261744	Netherlands	2022-Jan-11	A/Barnacle goose/Netherlands/2/2022	Erasmus Medical Center	Erasmus Medical Center	na
EPI_ISL_9261745	Netherlands	2022-Jan-11	A/Barnacle goose/Netherlands/3/2022	Erasmus Medical Center	Erasmus Medical Center	na
EPI_ISL_9261746	Netherlands	2022-Jan-11	A/Barnacle goose/Netherlands/4/2022	Erasmus Medical Center	Erasmus Medical Center	na
EPI_ISL_9261747	Netherlands	2022-Jan-14	A/Barnacle goose/Netherlands/5/2022	Erasmus Medical Center	Erasmus Medical Center	na
EPI_ISL_9261748	Netherlands	2022-Jan-14	A/Grey heron/Netherlands/1/2022	Erasmus Medical Center	Erasmus Medical Center	na
EPI_ISL_9261749	Netherlands	2022-Jan-14	A/Caspian gull/Netherlands/1/2022	Erasmus Medical Center	Erasmus Medical Center	na
EPI_ISL_9261750	Netherlands	2022-Jan-14	A/Black-headed gull/Netherlands/1/2022	Erasmus Medical Center	Erasmus Medical Center	na
EPI_ISL_9261751	Netherlands	2022-Jan-14	A/Barnacle goose/Netherlands/6/2022	Erasmus Medical Center	Erasmus Medical Center	na
EPI_ISL_9304992	Netherlands	2021-Dec-31	A/barnacle goose/Netherlands/22000551-002/2022	Wageningen Bioveterinary Research	Wageningen Bioveterinary Research	Beerens, Nancy; Harders, Frank; Pritz-Verschuren, Sylvia; Roose, Marit; Venema, Sandra; Germeraad, Evelien; Engelsma, Marc; Heutink, Rene
EPI_ISL_9377021	France	2021-Dec-23	A/goose/France/21P014207/2021	Anses (Ploufragan-Plouzané)	ANSES Agence Nationale De Securite Sanitaire De L'alimentation	na
EPI_ISL_9401912	Croatia	2022-Jan-20	A/white-fronted Goose/Croatia/16/2022	Croatian Veterinary Institute, Poultry Centre	Croatian Veterinary Institute	Savić, Vladimir
EPI_ISL_9439410	Netherlands	2021-Dec-30	A/grey heron/Netherlands/22000049-002/2021	Wageningen Bioveterinary Research	Wageningen Bioveterinary Research	Beerens, Nancy; Harders, Frank; Pritz-Verschuren, Sylvia; Roose, Marit; Venema, Sandra; Germeraad, Evelien; Engelsma, Marc; Heutink, Rene
EPI_ISL_9603767	Czech Republic	2021-Nov-26	A/goose/Czech Republic/23458-2T/2021	State Veterinary Institute Prague	State Veterinary Institute Prague	Alexander,Nagy;Lenka,Cernikova;Martina,Stara
EPI_ISL_9603769	Czech Republic	2021-Nov-26	A/goose/Czech Republic/23458-5T/2021	State Veterinary Institute Prague	State Veterinary Institute Prague	Alexander,Nagy;Lenka,Cernikova;Martina,Stara
EPI_ISL_9603775	Czech Republic	2021-Nov-26	A/goose/Czech Republic/23458-4T/2021	State Veterinary Institute Prague	State Veterinary Institute Prague	Alexander,Nagy;Lenka,Cernikova;Martina,Stara
EPI_ISL_9603780	Czech Republic	2021-Nov-26	A/goose/Czech Republic/23458-1K/2021	State Veterinary Institute Prague	State Veterinary Institute Prague	Alexander,Nagy;Lenka,Cernikova;Martina,Stara

EPI_ISL_9603795	Czech Republic	2021-Nov-18	A/goose/Czech Republic/22608-1T/2021	State Veterinary Institute Prague	State Veterinary Institute Prague	Alexander,Nagy;Lenka,Cernikova;Martina,Stara
EPI_ISL_9603819	Czech Republic	2021-Nov-18	A/goose/Czech Republic/22608-2T/2021	State Veterinary Institute Prague	State Veterinary Institute Prague	Alexander,Nagy;Lenka,Cernikova;Martina,Stara
EPI_ISL_9603901	Czech Republic	2021-Nov-18	A/goose/Czech Republic/22608-3T/2021	State Veterinary Institute Prague	State Veterinary Institute Prague	Alexander,Nagy;Lenka,Cernikova;Martina,Stara
EPI_ISL_9603916	Czech Republic	2021-Nov-12	A/mute swan/Czech Republic/22477-1/2021	State Veterinary Institute Prague	State Veterinary Institute Prague	Alexander,Nagy;Lenka,Cernikova;Martina,Stara
EPI_ISL_9603918	Czech Republic	2021-Nov-12	A/mute swan/Czech Republic/22477-2/2021	State Veterinary Institute Prague	State Veterinary Institute Prague	Alexander,Nagy;Lenka,Cernikova;Martina,Stara
EPI_ISL_9603920	Czech Republic	2021-Nov-15	A/mute swan/Czech Republic/22380/2021	State Veterinary Institute Prague	State Veterinary Institute Prague	Alexander,Nagy;Lenka,Cernikova;Martina,Stara
EPI_ISL_9603922	Czech Republic	2021-Nov-15	A/mute swan/Czech Republic/22684/2021	State Veterinary Institute Prague	State Veterinary Institute Prague	Alexander,Nagy;Lenka,Cernikova;Martina,Stara
EPI_ISL_9603924	Czech Republic	2021-Nov-19	A/goose/Czech Republic/22750/2021	State Veterinary Institute Prague	State Veterinary Institute Prague	Alexander,Nagy;Lenka,Cernikova;Martina,Stara
EPI_ISL_9603927	Czech Republic	2021-Dec-20	A/mute swan/Czech Republic/25702-2/2021	State Veterinary Institute Prague	State Veterinary Institute Prague	Alexander,Nagy;Lenka,Cernikova;Martina,Stara
EPI_ISL_9603929	Czech Republic	2021-Dec-22	A/chicken/Czech Republic/25690/2021	State Veterinary Institute Prague	State Veterinary Institute Prague	Alexander,Nagy;Lenka,Cernikova;Martina,Stara
EPI_ISL_9603931	Czech Republic	2021-Dec-25	A/pheasant/Czech Republic/25827-1/2021	State Veterinary Institute Prague	State Veterinary Institute Prague	Alexander,Nagy;Lenka,Cernikova;Martina,Stara
EPI_ISL_9603937	Czech Republic	2021-Dec-25	A/pheasant/Czech Republic/25827-2/2021	State Veterinary Institute Prague	State Veterinary Institute Prague	Alexander,Nagy;Lenka,Cernikova;Martina,Stara
EPI_ISL_9603939	Czech Republic	2021-Dec-25	A/pheasant/Czech Republic/25827-3/2021	State Veterinary Institute Prague	State Veterinary Institute Prague	Alexander,Nagy;Lenka,Cernikova;Martina,Stara
EPI_ISL_9603943	Czech Republic	2022-Jan-10	A/mute swan/Czech Republic/785/2022	State Veterinary Institute Prague	State Veterinary Institute Prague	Alexander,Nagy;Lenka,Cernikova;Martina,Stara
EPI_ISL_9616212	Netherlands	2022-Jan-06	A/red knot/Netherlands/22000409-002/2022	Wageningen Bioveterinary Research	Wageningen Bioveterinary Research	Beerens, Nancy; Harders, Frank; Pritz-Verschuren, Sylvia; Roose, Marit; Venema, Sandra; Germeraad, Evelien; Engelsma, Marc; Heutink, Rene
EPI_ISL_9690841	Netherlands	2022-Jan-03	A/great black-backed gull/Netherlands/22000090-002/2022	Wageningen Bioveterinary Research	Wageningen Bioveterinary Research	Beerens, Nancy; Harders, Frank; Pritz-Verschuren, Sylvia; Roose, Marit; Venema, Sandra; Germeraad, Evelien; Engelsma, Marc; Heutink, Rene
EPI_ISL_9856775	Netherlands	2021-Oct-25	A/chicken/Netherlands/21037287-006010/2021	Wageningen Bioveterinary Research	Wageningen Bioveterinary Research	Beerens, Nancy; Harders, Frank; Pritz-Verschuren, Sylvia; Roose, Marit; Venema, Sandra; Germeraad, Evelien; Engelsma, Marc; Heutink, Rene; Luca, Bordes
EPI_ISL_10261376	Germany	2022-Jan-24	A/pigeon/Germany-NW/AI00951/2022	Chemisches- und Veterinäruntersuchungsamt Westfalen	Friedrich-Loeffler-Institut	na

EPI_ISL_10347218	Netherlands	2021-Dec-29	A/Barnacle Goose/Netherlands/6/2021	Erasmus Medical Center	Erasmus Medical Center	na
EPI_ISL_10347219	Netherlands	2022-Jan-28	A/Barnacle Goose/Netherlands/8/2022	Erasmus Medical Center	Erasmus Medical Center	na
EPI_ISL_10347220	Netherlands	2021-Dec-29	A/Barnacle Goose/Netherlands/7/2021	Erasmus Medical Center	Erasmus Medical Center	na
EPI_ISL_10347326	Germany	2021-Dec-28	A/long-eared owl/Germany-NI/AI09037/2021	Lebensmittel- und Veterinärinstitut Oldenburg - Standort Veterinärinstitut	Friedrich-Loeffler-Institut	na
EPI_ISL_10497305	Germany	2022-Jan-25	A/red knot/Germany-SH/AI01010/2022	Landeslabor Schleswig-Holstein	Friedrich-Loeffler-Institut	na
EPI_ISL_10497306	Germany	2022-Feb-11	A/buzzard/Germany-BB/AI01212/2022	Landeslabor Berlin-Brandenburg	Friedrich-Loeffler-Institut	na
EPI_ISL_10576444	Belgium	2022-Jan-23	A/Anser_anser/Belgium/1809_0002/2022	Sciensano - Animal Infectious Diseases	Sciensano, Department of Animal Infectious Diseases	Van Borm, Steven; Roupie, Virginie; Lambrecht, Benedicte; Mathijs, Elisabeth; Steensels, Mieke
EPI_ISL_10724257	Netherlands	2021-Dec-23	A/sanderling/Netherlands/22002272-001/2021	Wageningen Bioveterinary Research	Wageningen Bioveterinary Research	Beerens, Nancy; Harders, Frank; Pritz-Verschuren, Sylvia; Roose, Marit; Venema, Sandra; Germeraad, Evelien; Engelsma, Marc; Heutink, Rene
EPI_ISL_10724259	Netherlands	2022-Feb-01	A/seagull/Netherlands/22002274-002/2022	Wageningen Bioveterinary Research	Wageningen Bioveterinary Research	Beerens, Nancy; Harders, Frank; Pritz-Verschuren, Sylvia; Roose, Marit; Venema, Sandra; Germeraad, Evelien; Engelsma, Marc; Heutink, Rene
EPI_ISL_10943657	Sweden	2022-Jan-25	A/northern goshawk/Sweden/SVA220210SZ0305/FP000317/O-2022	National Veterinary Institute, SVA	National Veterinary Institute	na
EPI_ISL_10992813	Netherlands	2022-Feb-12	A/Mallard/Netherlands/1/2022	Erasmus Medical Center	Erasmus Medical Center	na
EPI_ISL_10993074	Netherlands	2022-Feb-14	A/Mallard/Netherlands/2/2022	Erasmus Medical Center	Erasmus Medical Center	na
EPI_ISL_10993075	Netherlands	2021-Nov-29	A/Mallard/Netherlands/4/2021	Erasmus Medical Center	Erasmus Medical Center	na
EPI_ISL_10993076	Netherlands	2021-Nov-29	A/Mallard/Netherlands/5/2021	Erasmus Medical Center	Erasmus Medical Center	na
EPI_ISL_10993105	Netherlands	2021-Nov-29	A/Mallard/Netherlands/6/2021	Erasmus Medical Center	Erasmus Medical Center	na
EPI_ISL_10993134	Netherlands	2021-Nov-29	A/Mallard/Netherlands/7/2021	Erasmus Medical Center	Erasmus Medical Center	na
EPI_ISL_10993166	Netherlands	2021-Nov-29	A/Mallard/Netherlands/8/2021	Erasmus Medical Center	Erasmus Medical Center	na
EPI_ISL_10993196	Netherlands	2022-Jan-29	A/Sanderling/Netherlands/1/2022	Erasmus Medical Center	Erasmus Medical Center	na

EPI_ISL_11007527	Moldova, Republic of	2022-Jan-03	A/laying_hen/Moldova/68-1_22VIR638-1/2022	Republican Center of Veterinary Diagnostics	Istituto Zooprofilattico Sperimentale delle Venezie	Arseniev, S.; Groza, O.; Barbierato, G.; Zecchin, B.; Fusaro, A.; Schivo, A.; Salviato, A.; Palumbo, E.; Giussani, E.; Monne, I.; Terregino, C.
EPI_ISL_11007528	Slovenia	2021-Dec-26	A/rooster/Slovenia/2039_22VIR777-1/2021	University of Ljubljana	Istituto Zooprofilattico Sperimentale delle Venezie	Slavec, B.; Ra?nik, J.; Krape?, U.; ?labravec, Z.; A?ko, J.; Cociancich, V.; Paller, T.; Vidrih, ?.; Rojs, O.Z.; Arseniev, S.; Groza, O.; Barbierato, G.; Zecchin, B.; Fusaro, A.; Schivo, A.; Salviato, A.; Palumbo, E.; Giussani, E.; Monne, I.; Terregino, C.
EPI_ISL_11007529	Slovenia	2021-Dec-26	A/swan/Slovenia/2041_22VIR777-2/2021	University of Ljubljana	Istituto Zooprofilattico Sperimentale delle Venezie	Slavec, B.; Ra?nik, J.; Krape?, U.; ?labravec, Z.; A?ko, J.; Cociancich, V.; Paller, T.; Vidrih, ?.; Rojs, O.Z.; Arseniev, S.; Groza, O.; Barbierato, G.; Zecchin, B.; Fusaro, A.; Schivo, A.; Salviato, A.; Palumbo, E.; Giussani, E.; Monne, I.; Terregino, C.
EPI_ISL_11007530	Slovenia	2021-Dec-27	A/swan/Slovenia/2049_22VIR777-3/2021	University of Ljubljana	Istituto Zooprofilattico Sperimentale delle Venezie	Slavec, B.; Ra?nik, J.; Krape?, U.; ?labravec, Z.; A?ko, J.; Cociancich, V.; Paller, T.; Vidrih, ?.; Rojs, O.Z.; Arseniev, S.; Groza, O.; Barbierato, G.; Zecchin, B.; Fusaro, A.; Schivo, A.; Salviato, A.; Palumbo, E.; Giussani, E.; Monne, I.; Terregino, C.
EPI_ISL_11007531	Slovenia	2021-Dec-29	A/swan/Slovenia/2060_22VIR777-4/2021	University of Ljubljana	Istituto Zooprofilattico Sperimentale delle Venezie	Slavec, B.; Ra?nik, J.; Krape?, U.; ?labravec, Z.; A?ko, J.; Cociancich, V.; Paller, T.; Vidrih, ?.; Rojs, O.Z.; Arseniev, S.; Groza, O.; Barbierato, G.; Zecchin, B.; Fusaro, A.; Schivo, A.; Salviato, A.; Palumbo, E.; Giussani, E.; Monne, I.; Terregino, C.
EPI_ISL_11007532	Slovenia	2021-Dec-30	A/swan/Slovenia/2072_22VIR777-5/2021	University of Ljubljana	Istituto Zooprofilattico Sperimentale delle Venezie	Slavec, B.; Ra?nik, J.; Krape?, U.; ?labravec, Z.; A?ko, J.; Cociancich, V.; Paller, T.; Vidrih, ?.; Rojs, O.Z.; Arseniev, S.; Groza, O.; Barbierato, G.; Zecchin, B.; Fusaro, A.; Schivo, A.; Salviato, A.; Palumbo, E.; Giussani, E.; Monne, I.; Terregino, C.
EPI_ISL_11007533	Slovenia	2021-Dec-30	A/swan/Slovenia/2073_22VIR777-6/2021	University of Ljubljana	Istituto Zooprofilattico Sperimentale delle Venezie	Slavec, B.; Ra?nik, J.; Krape?, U.; ?labravec, Z.; A?ko, J.; Cociancich, V.; Paller, T.; Vidrih, ?.; Rojs, O.Z.; Arseniev, S.; Groza, O.; Barbierato, G.; Zecchin, B.; Fusaro, A.; Schivo, A.; Salviato, A.; Palumbo, E.; Giussani, E.; Monne, I.; Terregino, C.
EPI_ISL_11007534	Slovenia	2021-Dec-30	A/seagull/Slovenia/2075_22VIR777-7/2021	University of Ljubljana	Istituto Zooprofilattico Sperimentale delle Venezie	Slavec, B.; Ra?nik, J.; Krape?, U.; ?labravec, Z.; A?ko, J.; Cociancich, V.; Paller, T.; Vidrih, ?.; Rojs, O.Z.; Arseniev, S.; Groza, O.; Barbierato, G.; Zecchin, B.; Fusaro, A.; Schivo, A.; Salviato, A.; Palumbo, E.; Giussani, E.; Monne, I.; Terregino, C.
EPI_ISL_11007535	Slovenia	2022-Jan-03	A/swan/Slovenia/13_22VIR777-8/2022	University of Ljubljana	Istituto Zooprofilattico Sperimentale delle Venezie	Slavec, B.; Ra?nik, J.; Krape?, U.; ?labravec, Z.; A?ko, J.; Cociancich, V.; Paller, T.; Vidrih, ?.; Rojs, O.Z.; Arseniev, S.; Groza, O.; Barbierato, G.; Zecchin, B.; Fusaro, A.; Schivo, A.; Salviato, A.; Palumbo, E.; Giussani, E.; Monne, I.; Terregino, C.

EPI_ISL_11007537	Bulgaria	2021-Dec-29	A/hen/Bulgaria/854-1_22VIR778-10/2021	NDRVMI (National Diagnostic and Research Veterinary Medical Institute)	Istituto Zooprofilattico Sperimentale delle Venezie	Goujgoulova, G.; Slavcheva, I.; Oreshkova, L.; Barbierato, G.; Zecchin, B.; Fusaro, A.; Schivo, A.; Salviato, A.; Palumbo, E.; Giussani, E.; Monne, I.; Terregino, C.
EPI_ISL_11007538	Bulgaria	2021-Nov-15	A/hen/Bulgaria/722-1_22VIR778-1/2021	NDRVMI (National Diagnostic and Research Veterinary Medical Institute)	Istituto Zooprofilattico Sperimentale delle Venezie	Goujgoulova, G.; Slavcheva, I.; Oreshkova, L.; Barbierato, G.; Zecchin, B.; Fusaro, A.; Schivo, A.; Salviato, A.; Palumbo, E.; Giussani, E.; Monne, I.; Terregino, C.
EPI_ISL_11007539	Bulgaria	2021-Nov-30	A/turkey/Bulgaria/755-1_22VIR778-4/2021	NDRVMI (National Diagnostic and Research Veterinary Medical Institute)	Istituto Zooprofilattico Sperimentale delle Venezie	Goujgoulova, G.; Slavcheva, I.; Oreshkova, L.; Barbierato, G.; Zecchin, B.; Fusaro, A.; Schivo, A.; Salviato, A.; Palumbo, E.; Giussani, E.; Monne, I.; Terregino, C.
EPI_ISL_11007540	Bulgaria	2021-Dec-01	A/duck/Bulgaria/756-4_22VIR778-6/2021	NDRVMI (National Diagnostic and Research Veterinary Medical Institute)	Istituto Zooprofilattico Sperimentale delle Venezie	Goujgoulova, G.; Slavcheva, I.; Oreshkova, L.; Barbierato, G.; Zecchin, B.; Fusaro, A.; Schivo, A.; Salviato, A.; Palumbo, E.; Giussani, E.; Monne, I.; Terregino, C.
EPI_ISL_11007541	Bulgaria	2021-Dec-02	A/hen/Bulgaria/757-6_22VIR778-7/2021	NDRVMI (National Diagnostic and Research Veterinary Medical Institute)	Istituto Zooprofilattico Sperimentale delle Venezie	Goujgoulova, G.; Slavcheva, I.; Oreshkova, L.; Barbierato, G.; Zecchin, B.; Fusaro, A.; Schivo, A.; Salviato, A.; Palumbo, E.; Giussani, E.; Monne, I.; Terregino, C.
EPI_ISL_11007721	Moldova, Republic of	2022-Jan-03	A/laying_hen/Moldova/68-2_22VIR638-2/2022	Republican Center of Veterinary Diagnostics	Istituto Zooprofilattico Sperimentale delle Venezie	Arseniev, S.; Groza, O.; Barbierato, G.; Zecchin, B.; Fusaro, A.; Schivo, A.; Salviato, A.; Palumbo, E.; Giussani, E.; Monne, I.; Terregino, C.
EPI_ISL_11007722	Bulgaria	2021-Nov-23	A/partridge/Bulgaria/745_22VIR778-3/2021	NDRVMI (National Diagnostic and Research Veterinary Medical Institute)	Istituto Zooprofilattico Sperimentale delle Venezie	Goujgoulova, G.; Slavcheva, I.; Oreshkova, L.; Barbierato, G.; Zecchin, B.; Fusaro, A.; Schivo, A.; Salviato, A.; Palumbo, E.; Giussani, E.; Monne, I.; Terregino, C.
EPI_ISL_11009382	Bulgaria	2021-Dec-16	A/duck/Bulgaria/827-2_22VIR778-8/2021	NDRVMI (National Diagnostic and Research Veterinary Medical Institute)	Istituto Zooprofilattico Sperimentale Delle Venezie	Goujgoulova, G.; Slavcheva, I.; Oreshkova, L.; Barbierato, G.; Zecchin, B.; Fusaro, A.; Schivo, A.; Salviato, A.; Palumbo, E.; Giussani, E.; Monne, I.; Terregino, C.
EPI_ISL_11112340	Netherlands	2022-Feb-07	A/Barnacle Goose/Netherlands/10/2022	Erasmus Medical Center	Erasmus Medical Center	na
EPI_ISL_11112342	Netherlands	2022-Feb-09	A/Barnacle Goose/Netherlands/11/2022	Erasmus Medical Center	Erasmus Medical Center	na
EPI_ISL_11112344	Netherlands	2022-Feb-09	A/Barnacle Goose/Netherlands/12/2022	Erasmus Medical Center	Erasmus Medical Center	na
EPI_ISL_11112346	Netherlands	2022-Feb-23	A/Barnacle Goose/Netherlands/13/2022	Erasmus Medical Center	Erasmus Medical Center	na
EPI_ISL_11112348	Netherlands	2022-Feb-23	A/Barnacle Goose/Netherlands/14/2022	Erasmus Medical Center	Erasmus Medical Center	na
EPI_ISL_11112350	Netherlands	2022-Feb-26	A/Barnacle Goose/Netherlands/15/2022	Erasmus Medical Center	Erasmus Medical Center	na

EPI_ISL_11112352	Netherlands	2022-Feb-07	A/Barnacle Goose/Netherlands/9/2022	Erasmus Medical Center	Erasmus Medical Center	na
EPI_ISL_11112354	Netherlands	2022-Feb-01	A/Common Gull/Netherlands/1/2022	Erasmus Medical Center	Erasmus Medical Center	na
EPI_ISL_11112356	Netherlands	2022-Feb-09	A/Eurasian Curlew/Netherlands/1/2022	Erasmus Medical Center	Erasmus Medical Center	na
EPI_ISL_11112358	Netherlands	2022-Feb-26	A/European Herring Gull/Netherlands/2/2022	Erasmus Medical Center	Erasmus Medical Center	na
EPI_ISL_11112360	Netherlands	2022-Feb-23	A/Great black-backed Gull/Netherlands/2/2022	Erasmus Medical Center	Erasmus Medical Center	na
EPI_ISL_11112362	Netherlands	2022-Feb-02	A/Greylag Goose/Netherlands/1/2022	Erasmus Medical Center	Erasmus Medical Center	na
EPI_ISL_11112484	Netherlands	2022-Feb-23	A/Caspian Gull/Netherlands/2/2022	Erasmus Medical Center	Erasmus Medical Center	na
EPI_ISL_11259261	Ireland	2021-Nov-17	A/herring_gull/Ireland/033533_22VIR1325-10/2021	Central Veterinary Research Laboratory	Istituto Zooprofilattico Sperimentale delle Venezie	Byrne, C.; Garcia, K.; Cuartero, L.G.; Barbierato, G.; Zecchin, B.; Fusaro, A.; Schivo, A.; Salviato, A.; Palumbo, E.; Giussani, E.; Monne, I.; Terregino, C.
EPI_ISL_11259262	Ireland	2021-Dec-08	A/crow/Ireland/035624_22VIR1325-11/2021	Central Veterinary Research Laboratory	Istituto Zooprofilattico Sperimentale delle Venezie	Byrne, C.; Garcia, K.; Cuartero, L.G.; Barbierato, G.; Zecchin, B.; Fusaro, A.; Schivo, A.; Salviato, A.; Palumbo, E.; Giussani, E.; Monne, I.; Terregino, C.
EPI_ISL_11259263	Ireland	2022-Jan-07	A/buzzard/Ireland/000656_22VIR1325-12/2022	Central Veterinary Research Laboratory	Istituto Zooprofilattico Sperimentale delle Venezie	Byrne, C.; Garcia, K.; Cuartero, L.G.; Barbierato, G.; Zecchin, B.; Fusaro, A.; Schivo, A.; Salviato, A.; Palumbo, E.; Giussani, E.; Monne, I.; Terregino, C.
EPI_ISL_11259264	Ireland	2021-Nov-15	A/mute_swan/Ireland/033169_22VIR1325-14/2021	Central Veterinary Research Laboratory	Istituto Zooprofilattico Sperimentale delle Venezie	Byrne, C.; Garcia, K.; Cuartero, L.G.; Barbierato, G.; Zecchin, B.; Fusaro, A.; Schivo, A.; Salviato, A.; Palumbo, E.; Giussani, E.; Monne, I.; Terregino, C.
EPI_ISL_11259265	Ireland	2022-Jan-05	A/peregrine_falcon/Ireland/000191_22VIR1325-15/2022	Central Veterinary Research Laboratory	Istituto Zooprofilattico Sperimentale delle Venezie	Byrne, C.; Garcia, K.; Cuartero, L.G.; Barbierato, G.; Zecchin, B.; Fusaro, A.; Schivo, A.; Salviato, A.; Palumbo, E.; Giussani, E.; Monne, I.; Terregino, C.
EPI_ISL_11259266	Ireland	2021-Nov-19	A/mute_swan/Ireland/033945_22VIR1325-16/2021	Central Veterinary Research Laboratory	Istituto Zooprofilattico Sperimentale delle Venezie	Byrne, C.; Garcia, K.; Cuartero, L.G.; Barbierato, G.; Zecchin, B.; Fusaro, A.; Schivo, A.; Salviato, A.; Palumbo, E.; Giussani, E.; Monne, I.; Terregino, C.
EPI_ISL_11259267	Ireland	2021-Dec-06	A/turkey/Ireland/035425_22VIR1325-17/2021	Central Veterinary Research Laboratory	Istituto Zooprofilattico Sperimentale delle Venezie	Byrne, C.; Garcia, K.; Cuartero, L.G.; Barbierato, G.; Zecchin, B.; Fusaro, A.; Schivo, A.; Salviato, A.; Palumbo, E.; Giussani, E.; Monne, I.; Terregino, C.
EPI_ISL_11259268	Ireland	2021-Dec-17	A/duck/Ireland/036646_22VIR1325-18/2021	Central Veterinary Research Laboratory	Istituto Zooprofilattico Sperimentale delle Venezie	Byrne, C.; Garcia, K.; Cuartero, L.G.; Barbierato, G.; Zecchin, B.; Fusaro, A.; Schivo, A.; Salviato, A.; Palumbo, E.; Giussani, E.; Monne, I.; Terregino, C.
EPI_ISL_11259269	Ireland	2021-Nov-19	A/turkey/Ireland/033674_22VIR1325-19/2021	Central Veterinary Research Laboratory	Istituto Zooprofilattico Sperimentale delle Venezie	Byrne, C.; Garcia, K.; Cuartero, L.G.; Barbierato, G.; Zecchin, B.; Fusaro, A.; Schivo, A.; Salviato, A.; Palumbo, E.; Giussani, E.; Monne, I.; Terregino, C.

EPI_ISL_11259270	Ireland	2021-Nov-08	A/mute_swan/Ireland/032363_22VIR1325-1/2021	Central Veterinary Research Laboratory	Istituto Zooprofilattico Sperimentale delle Venezie	Byrne, C.; Garcia, K.; Cuartero, L.G.; Barbierato, G.; Zecchin, B.; Fusaro, A.; Schivo, A.; Salviato, A.; Palumbo, E.; Giussani, E.; Monne, I.; Terregino, C.
EPI_ISL_11259271	Ireland	2021-Nov-22	A/broiler/Ireland/033734_22VIR1325-20/2021	Central Veterinary Research Laboratory	Istituto Zooprofilattico Sperimentale delle Venezie	Byrne, C.; Garcia, K.; Cuartero, L.G.; Barbierato, G.; Zecchin, B.; Fusaro, A.; Schivo, A.; Salviato, A.; Palumbo, E.; Giussani, E.; Monne, I.; Terregino, C.
EPI_ISL_11259272	Ireland	2021-Nov-29	A/layer/Ireland/034424_22VIR1325-21/2021	Central Veterinary Research Laboratory	Istituto Zooprofilattico Sperimentale delle Venezie	Byrne, C.; Garcia, K.; Cuartero, L.G.; Barbierato, G.; Zecchin, B.; Fusaro, A.; Schivo, A.; Salviato, A.; Palumbo, E.; Giussani, E.; Monne, I.; Terregino, C.
EPI_ISL_11259273	Ireland	2021-Dec-11	A/duck/Ireland/036105_22VIR1325-22/2021	Central Veterinary Research Laboratory	Istituto Zooprofilattico Sperimentale delle Venezie	Byrne, C.; Garcia, K.; Cuartero, L.G.; Barbierato, G.; Zecchin, B.; Fusaro, A.; Schivo, A.; Salviato, A.; Palumbo, E.; Giussani, E.; Monne, I.; Terregino, C.
EPI_ISL_11259274	Ireland	2021-Nov-03	A/white-tailed_eagle/Ireland/032034_22VIR1325-23/2021	Central Veterinary Research Laboratory	Istituto Zooprofilattico Sperimentale delle Venezie	Byrne, C.; Garcia, K.; Cuartero, L.G.; Barbierato, G.; Zecchin, B.; Fusaro, A.; Schivo, A.; Salviato, A.; Palumbo, E.; Giussani, E.; Monne, I.; Terregino, C.
EPI_ISL_11259275	Ireland	2021-Nov-09	A/whooper_swan/Ireland/032444_22VIR1325-2/2021	Central Veterinary Research Laboratory	Istituto Zooprofilattico Sperimentale delle Venezie	Byrne, C.; Garcia, K.; Cuartero, L.G.; Barbierato, G.; Zecchin, B.; Fusaro, A.; Schivo, A.; Salviato, A.; Palumbo, E.; Giussani, E.; Monne, I.; Terregino, C.
EPI_ISL_11259276	Ireland	2021-Nov-10	A/peregrine_falcon/Ireland/032476_22VIR1325-3/2021	Central Veterinary Research Laboratory	Istituto Zooprofilattico Sperimentale delle Venezie	Byrne, C.; Garcia, K.; Cuartero, L.G.; Barbierato, G.; Zecchin, B.; Fusaro, A.; Schivo, A.; Salviato, A.; Palumbo, E.; Giussani, E.; Monne, I.; Terregino, C.
EPI_ISL_11259277	Ireland	2021-Nov-12	A/whooper_swan/Ireland/032960_22VIR1325-4/2021	Central Veterinary Research Laboratory	Istituto Zooprofilattico Sperimentale delle Venezie	Byrne, C.; Garcia, K.; Cuartero, L.G.; Barbierato, G.; Zecchin, B.; Fusaro, A.; Schivo, A.; Salviato, A.; Palumbo, E.; Giussani, E.; Monne, I.; Terregino, C.
EPI_ISL_11259278	Ireland	2021-Nov-12	A/magpie/Ireland/032958_22VIR1325-5/2021	Central Veterinary Research Laboratory	Istituto Zooprofilattico Sperimentale delle Venezie	Byrne, C.; Garcia, K.; Cuartero, L.G.; Barbierato, G.; Zecchin, B.; Fusaro, A.; Schivo, A.; Salviato, A.; Palumbo, E.; Giussani, E.; Monne, I.; Terregino, C.
EPI_ISL_11259279	Ireland	2021-Nov-15	A/greylag_goose/Ireland/032969_22VIR1325-6/2021	Central Veterinary Research Laboratory	Istituto Zooprofilattico Sperimentale delle Venezie	Byrne, C.; Garcia, K.; Cuartero, L.G.; Barbierato, G.; Zecchin, B.; Fusaro, A.; Schivo, A.; Salviato, A.; Palumbo, E.; Giussani, E.; Monne, I.; Terregino, C.
EPI_ISL_11259280	Ireland	2021-Nov-15	A/greylag_goose/Ireland/033062_22VIR1325-7/2021	Central Veterinary Research Laboratory	Istituto Zooprofilattico Sperimentale delle Venezie	Byrne, C.; Garcia, K.; Cuartero, L.G.; Barbierato, G.; Zecchin, B.; Fusaro, A.; Schivo, A.; Salviato, A.; Palumbo, E.; Giussani, E.; Monne, I.; Terregino, C.
EPI_ISL_11259281	Ireland	2021-Nov-15	A/white-fronted_goose/Ireland/033181_22VIR1325-8/2021	Central Veterinary Research Laboratory	Istituto Zooprofilattico Sperimentale delle Venezie	Byrne, C.; Garcia, K.; Cuartero, L.G.; Barbierato, G.; Zecchin, B.; Fusaro, A.; Schivo, A.; Salviato, A.; Palumbo, E.; Giussani, E.; Monne, I.; Terregino, C.
EPI_ISL_11259282	Ireland	2021-Nov-16	A/brent_goose/Ireland/033257_22VIR1325-9/2021	Central Veterinary Research Laboratory	Istituto Zooprofilattico Sperimentale delle Venezie	Byrne, C.; Garcia, K.; Cuartero, L.G.; Barbierato, G.; Zecchin, B.; Fusaro, A.; Schivo, A.; Salviato, A.; Palumbo, E.; Giussani, E.; Monne, I.; Terregino, C.
EPI_ISL_11259283	Ireland	2022-Feb-14	A/fox/Ireland/3866_22VIR2064-1/2022	Central Veterinary Research Laboratory	Istituto Zooprofilattico Sperimentale delle Venezie	Byrne, C.; Garcia, K.; Cuartero, L.G.; Barbierato, G.; Zecchin, B.; Fusaro, A.; Schivo, A.; Salviato, A.; Palumbo, E.; Giussani, E.; Monne, I.; Terregino, C.

EPI_ISL_11268632	Croatia	2022-Feb-09	A/mute swan/Croatia/26/2022	Croatian Veterinary Institute, Poultry Centre	Croatian Veterinary Institute	Savić, Vladimir
EPI_ISL_11327088	Czech Republic	2021-Dec-31	A/chicken/Czech Republic/61-1/2022	State Veterinary Institute Prague	State Veterinary Institute Prague	Alexander,Nagy;Lenka,Cernikova;Martina,Stara
EPI_ISL_11327089	Czech Republic	2021-Dec-31	A/chicken/Czech Republic/61-2/2022	State Veterinary Institute Prague	State Veterinary Institute Prague	Alexander,Nagy;Lenka,Cernikova;Martina,Stara
EPI_ISL_11327090	Czech Republic	2022-Jan-03	A/chicken/Czech Republic/63/2022	State Veterinary Institute Prague	State Veterinary Institute Prague	Alexander,Nagy;Lenka,Cernikova;Martina,Stara
EPI_ISL_11406398	United Kingdom	2022-Jan-06	A/chicken/England/002070/2022	Animal and Plant Health Agency (APHA)	Animal and Plant Health Agency (APHA)	na
EPI_ISL_11406399	United Kingdom	2022-Jan-12	A/turkey/England/004737/2022	Animal and Plant Health Agency (APHA)	Animal and Plant Health Agency (APHA)	na
EPI_ISL_11406401	United Kingdom	2022-Jan-03	A/chicken/England/000187/2022	Animal and Plant Health Agency (APHA)	Animal and Plant Health Agency (APHA)	na
EPI_ISL_11406402	United Kingdom	2022-Jan-23	A/domestic_duck/England/007588/2022	Animal and Plant Health Agency (APHA)	Animal and Plant Health Agency (APHA)	na
EPI_ISL_11406403	United Kingdom	2022-Jan-31	A/black-headed_gull/England/306270/2022	Animal and Plant Health Agency (APHA)	Animal and Plant Health Agency (APHA)	na
EPI_ISL_11449674	Belgium	2022-Mar-10	A/Gallus_gallus/Belgium/3194_0001/2022	Sciensano - Animal Infectious Diseases	Sciensano, Department of Animal Infectious Diseases	Van Borm, Steven; Roupie, Virginie; Lambrecht, Benedicte; Mathijs, Elisabeth; Steensels, Mieke
EPI_ISL_11560233	Netherlands	2022-Mar-03	A/Brent goose/Netherlands/1/2022	Erasmus Medical Center	Erasmus Medical Center	na
EPI_ISL_11560235	Netherlands	2022-Mar-03	A/Barnacle Goose/Netherlands/16/2022	Erasmus Medical Center	Erasmus Medical Center	na
EPI_ISL_11560236	Netherlands	2022-Mar-03	A/Barnacle Goose/Netherlands/17/2022	Erasmus Medical Center	Erasmus Medical Center	na
EPI_ISL_11560323	Netherlands	2022-Mar-04	A/Barnacle Goose/Netherlands/18/2022	Erasmus Medical Center	Erasmus Medical Center	na
EPI_ISL_11560324	Netherlands	2022-Mar-06	A/Graylag goose/Netherlands/2/20222	Erasmus Medical Center	Erasmus Medical Center	na
EPI_ISL_11560325	Netherlands	2022-Mar-15	A/Great black-backed Gull/Netherlands/3/2022	Erasmus Medical Center	Erasmus Medical Center	na
EPI_ISL_11561589	United Kingdom	2022-Feb-02	A/chicken/England/011981/2022	Animal and Plant Health Agency (APHA)	Animal and Plant Health Agency (APHA)	na
EPI_ISL_11561590	United Kingdom	2022-Feb-08	A/chicken/England/012967/2022	Animal and Plant Health Agency (APHA)	Animal and Plant Health Agency (APHA)	na
EPI_ISL_11561591	United Kingdom	2022-Feb-08	A/domestic_duck/England/012973/2022	Animal and Plant Health Agency (APHA)	Animal and Plant Health Agency (APHA)	na
EPI_ISL_11561592	United Kingdom	2022-Feb-20	A/turkey/England/016515/2022	Animal and Plant Health Agency (APHA)	Animal and Plant Health Agency (APHA)	na

EPI_ISL_11561593	United Kingdom	2022-Feb-03	A/black-headed_gull/England/388256/2022	Animal and Plant Health Agency (APHA)	Animal and Plant Health Agency (APHA)	na
EPI_ISL_11580255	Sweden	2022-Jan-21	A/Greylag goose/Sweden/SVA220308SZ0382/FB000559/M-2022	National Veterinary Institute, SVA	National Veterinary Institute	na
EPI_ISL_11725869	Germany	2021-Nov-02	A/domestic duck/Germany-BB/AI06239/2021	Landeslabor Berlin-Brandenburg	Friedrich-Loeffler-Institut	na
EPI_ISL_11725962	Germany	2021-Oct-29	A/chicken/Germany-BB/AI06219/2021	Landeslabor Berlin-Brandenburg	Friedrich-Loeffler-Institut	na
EPI_ISL_11725988	Germany	2021-Nov-02	A/chicken/Germany-BB/AI06242/2021	Landeslabor Berlin-Brandenburg	Friedrich-Loeffler-Institut	na
EPI_ISL_11725991	Germany	2021-Oct-26	A/barnacle goose/Germany-MV/AI06175/2021	Landesamt für Landwirtschaft, Lebensmittelsicherheit und Fischerei (LALLF)	Friedrich-Loeffler-Institut	na
EPI_ISL_11725994	Germany	2021-Nov-09	A/buzzard/Germany-SH/AI07099/2021	Landeslabor Schleswig-Holstein	Friedrich-Loeffler-Institut	na
EPI_ISL_11798568	Denmark	2021-Oct-26	A/barnacle_goose/Denmark/24273-1.02/2021-10-26	Statens Serumm Institute	Statens Serum Institute	Charlotte Hjulsager, Yuan Liang
EPI_ISL_11798569	Denmark	2021-Oct-30	A/barnacle_goose/Denmark/24342-1.02/2021-10-30	Statens Serumm Institute	Statens Serum Institute	Charlotte Hjulsager, Yuan Liang
EPI_ISL_11798570	Denmark	2021-Nov-02	A/chicken/Denmark/24357-11/2021-11-02	Statens Serumm Institute	Statens Serum Institute	Charlotte Hjulsager, Yuan Liang
EPI_ISL_11798571	Denmark	2021-Oct-25	A/common_buzzard/Denmark/24271-1.02/2021-10-25	Statens Serumm Institute	Statens Serum Institute	Charlotte Hjulsager, Yuan Liang
EPI_ISL_11798580	Denmark	2021-Oct-30	A/turkey/Denmark/24325-25/2021-10-30	Statens Serumm Institute	Statens Serum Institute	Charlotte Hjulsager, Yuan Liang
EPI_ISL_11798572	Denmark	2021-Oct-13	A/Eurasian_wigeon/Denmark/24066-9/2021-10-13	Statens Serumm Institute	Statens Serum Institute	Charlotte Hjulsager, Yuan Liang
EPI_ISL_11880371	Belgium	2022-Mar-28	A/Gallus_gallus/Belgium/4190_0002/2022	Sciensano - Animal Infectious Diseases	Sciensano, Department of Animal Infectious Diseases	Van Borm, Steven; Roupie, Virginie; Lambrecht, Benedicte; Mathijs, Elisabeth; Steensels, Mieke
EPI_ISL_11922808	Poland	2022-Mar-02	A/duck/Poland/H188_22VIR2515-2/2022	National Veterinary Research Institute	Istituto Zooprofilattico Sperimentale delle Venezie	Swieton, E.; Smietanka, K.; Barbierato, G.; Zecchin, B.; Fusaro, A.; Schivo, A.; Salviato, A.; Palumbo, E.; Giussani, E.; Monne, I.; Terregino, C.
EPI_ISL_11922809	Poland	2022-Feb-18	A/chicken/Poland/H157_22VIR2515-3/2022	National Veterinary Research Institute	Istituto Zooprofilattico Sperimentale delle Venezie	Swieton, E.; Smietanka, K.; Barbierato, G.; Zecchin, B.; Fusaro, A.; Schivo, A.; Salviato, A.; Palumbo, E.; Giussani, E.; Monne, I.; Terregino, C.
EPI_ISL_11922810	Poland	2022-Feb-08	A/duck/Poland/H126_22VIR2515-4/2022	National Veterinary Research Institute	Istituto Zooprofilattico Sperimentale delle Venezie	Swieton, E.; Smietanka, K.; Barbierato, G.; Zecchin, B.; Fusaro, A.; Schivo, A.; Salviato, A.; Palumbo, E.; Giussani, E.; Monne, I.; Terregino, C.

EPI_ISL_1922811	Poland	2022-Feb-05	A/goose/Poland/H124_22VIR2515-5/2022	National Veterinary Research Institute	Istituto Zooprofilattico Sperimentale delle Venezie	Swieton, E.; Smietanka, K.; Barbierato, G.; Zecchin, B.; Fusaro, A.; Schivo, A.; Salviato, A.; Palumbo, E.; Giussani, E.; Monne, I.; Terregino, C.
EPI_ISL_1922812	Poland	2022-Jan-23	A/chicken/Poland/H071_22VIR2515-6/2022	National Veterinary Research Institute	Istituto Zooprofilattico Sperimentale delle Venezie	Swieton, E.; Smietanka, K.; Barbierato, G.; Zecchin, B.; Fusaro, A.; Schivo, A.; Salviato, A.; Palumbo, E.; Giussani, E.; Monne, I.; Terregino, C.
EPI_ISL_1922813	Poland	2022-Feb-10	A/swan/Poland/MB078_22VIR2515-7/2022	National Veterinary Research Institute	Istituto Zooprofilattico Sperimentale delle Venezie	Swieton, E.; Smietanka, K.; Barbierato, G.; Zecchin, B.; Fusaro, A.; Schivo, A.; Salviato, A.; Palumbo, E.; Giussani, E.; Monne, I.; Terregino, C.
EPI_ISL_1922814	Poland	2022-Feb-17	A/swan/Poland/MB083_22VIR2515-8/2022	National Veterinary Research Institute	Istituto Zooprofilattico Sperimentale delle Venezie	Swieton, E.; Smietanka, K.; Barbierato, G.; Zecchin, B.; Fusaro, A.; Schivo, A.; Salviato, A.; Palumbo, E.; Giussani, E.; Monne, I.; Terregino, C.
EPI_ISL_1922815	Romania	2021-Dec-08	A/swan/Romania/16905_22VIR2749-1/2021	Institute for Diagnosis & Animal Health (IDAH)	Istituto Zooprofilattico Sperimentale delle Venezie	Barbuceanu, F.; Onita, I.; Neicut, A.; Motiu, R.; Burlacu, R.; Barbierato, G.; Zecchin, B.; Fusaro, A.; Schivo, A.; Salviato, A.; Palumbo, E.; Giussani, E.; Monne, I.; Terregino, C.
EPI_ISL_1922816	Romania	2022-Feb-01	A/swan/Romania/10324_22VIR2749-2/2022	Institute for Diagnosis & Animal Health (IDAH)	Istituto Zooprofilattico Sperimentale delle Venezie	Barbuceanu, F.; Onita, I.; Neicut, A.; Motiu, R.; Burlacu, R.; Barbierato, G.; Zecchin, B.; Fusaro, A.; Schivo, A.; Salviato, A.; Palumbo, E.; Giussani, E.; Monne, I.; Terregino, C.
EPI_ISL_1922817	Romania	2022-Feb-03	A/swan/Romania/10394_22VIR2749-3/2022	Institute for Diagnosis & Animal Health (IDAH)	Istituto Zooprofilattico Sperimentale delle Venezie	Barbuceanu, F.; Onita, I.; Neicut, A.; Motiu, R.; Burlacu, R.; Barbierato, G.; Zecchin, B.; Fusaro, A.; Schivo, A.; Salviato, A.; Palumbo, E.; Giussani, E.; Monne, I.; Terregino, C.
EPI_ISL_1922818	Romania	2022-Feb-09	A/swan/Romania/10455_22VIR2749-4/2022	Institute for Diagnosis & Animal Health (IDAH)	Istituto Zooprofilattico Sperimentale delle Venezie	Barbuceanu, F.; Onita, I.; Neicut, A.; Motiu, R.; Burlacu, R.; Barbierato, G.; Zecchin, B.; Fusaro, A.; Schivo, A.; Salviato, A.; Palumbo, E.; Giussani, E.; Monne, I.; Terregino, C.
EPI_ISL_1922819	Romania	2022-Feb-10	A/laying_hen/Romania/10470_22VIR2749-5/2022	Institute for Diagnosis & Animal Health (IDAH)	Istituto Zooprofilattico Sperimentale delle Venezie	Barbuceanu, F.; Onita, I.; Neicut, A.; Motiu, R.; Burlacu, R.; Barbierato, G.; Zecchin, B.; Fusaro, A.; Schivo, A.; Salviato, A.; Palumbo, E.; Giussani, E.; Monne, I.; Terregino, C.
EPI_ISL_1922820	Romania	2022-Feb-22	A/swan/Romania/10656_22VIR2749-6/2022	Institute for Diagnosis & Animal Health (IDAH)	Istituto Zooprofilattico Sperimentale delle Venezie	Barbuceanu, F.; Onita, I.; Neicut, A.; Motiu, R.; Burlacu, R.; Barbierato, G.; Zecchin, B.; Fusaro, A.; Schivo, A.; Salviato, A.; Palumbo, E.; Giussani, E.; Monne, I.; Terregino, C.
EPI_ISL_1922821	Romania	2022-Feb-23	A/swan/Romania/10678_22VIR2749-7/2022	Institute for Diagnosis & Animal Health (IDAH)	Istituto Zooprofilattico Sperimentale delle Venezie	Barbuceanu, F.; Onita, I.; Neicut, A.; Motiu, R.; Burlacu, R.; Barbierato, G.; Zecchin, B.; Fusaro, A.; Schivo, A.; Salviato, A.; Palumbo, E.; Giussani, E.; Monne, I.; Terregino, C.

EPI_ISL_11922822	Romania	2022-Mar-07	A/swan/Romania/10986_22VIR2749-8/2022	Institute for Diagnosis & Animal Health (IDAH)	Istituto Zooprofilattico Sperimentale delle Venezie	Barbuceanu, F.; Onita, I.; Neicut, A.; Motiu, R.; Burlacu, R.; Barbierato, G.; Zecchin, B.; Fusaro, A.; Schivo, A.; Salviato, A.; Palumbo, E.; Giussani, E.; Monne, I.; Terregino, C.
EPI_ISL_11798573	Denmark	2021-Oct-16	A/Eurasian_teal/Denmark/24115-2/2021-10-16	Statens Serumm Institute	Statens Serum Institute	Charlotte Hjulsager, Yuan Liang
EPI_ISL_11798574	Denmark	2021-Oct-24	A/Eurasian_wigeon/Denmark/24279-1/2021-10-24	Statens Serumm Institute	Statens Serum Institute	Charlotte Hjulsager, Yuan Liang
EPI_ISL_11798577	Denmark	2021-Oct-28	A/gray_heron/Denmark/24326-1.02/2021-10-28	Statens Serumm Institute	Statens Serum Institute	Charlotte Hjulsager, Yuan Liang
EPI_ISL_11798578	Denmark	2021-Oct-27	A/greylag_goose/Denmark/24309-1.01/2021-10-27	Statens Serumm Institute	Statens Serum Institute	Charlotte Hjulsager, Yuan Liang
EPI_ISL_11798579	Denmark	2021-Nov-01	A/greylag_goose/Denmark/24343-1.02/2021-11-01	Statens Serumm Institute	Statens Serum Institute	Charlotte Hjulsager, Yuan Liang
EPI_ISL_12066188	Netherlands	2022-Jan-26	A/Fox/Netherlands/EMC1/2022	Erasmus Medical Center	Erasmus Medical Center	R.A.M., Fouchier; S., Therwessen; O., Vuong; I., Chestakova; R., Sikkema; P., Lexmond; M., Pronk
EPI_ISL_12069288	Netherlands	2022-Jan-10	A/Fox/Netherlands/EMC2/2022	Erasmus Medical Center	Erasmus Medical Center	R.A.M Fouchier, S. Therwessen, O. Vuong, I. Chestakova, R. Sikkema, P. Lexmond, M. Pronk
EPI_ISL_12069289	Netherlands	2022-Jan-22	A/Fox/Netherlands/EMC3/2022	Erasmus Medical Center	Erasmus Medical Center	R.A.M., Fouchier; S., Thewessen; O., Vuong; I., Chestakova; R., Sikkema; P., Lexmond; M., Pronk
EPI_ISL_12069978	Netherlands	2022-Mar-28	A/greylag goose/Netherlands/22006190-002/2022	Wageningen Bioveterinary Research	Wageningen Bioveterinary Research	Beerens, Nancy; Harders, Frank; Pritz-Verschuren, Sylvia; Roose, Marit; Venema, Sandra; Germeraad, Evelien; Engelsma, Marc; Heutink, Rene
EPI_ISL_12069979	Netherlands	2022-Mar-27	A/black-backed gull/Netherlands/22006192-001/2022	Wageningen Bioveterinary Research	Wageningen Bioveterinary Research	Beerens, Nancy; Harders, Frank; Pritz-Verschuren, Sylvia; Roose, Marit; Venema, Sandra; Germeraad, Evelien; Engelsma, Marc; Heutink, Rene
EPI_ISL_12139999	Czech Republic	2022-Jan-10	A/mute swan/Czech Republic/785/2022	State Veterinary Institute Prague	State Veterinary Institute Prague	Alexander,Nagy;Martina,Stara;Lenka,Cernikova
EPI_ISL_12140437	Czech Republic	2022-Jan-12	A/duck/Czech Republic/913/2022	State Veterinary Institute Prague	State Veterinary Institute Prague	Alexander,Nagy;Martina,Stara;Lenka,Cernikova
EPI_ISL_12150657	Czech Republic	2022-Jan-12	A/goose/Czech Republic/913/2022	State Veterinary Institute Prague	State Veterinary Institute Prague	Alexander,Nagy;Martina,Stara;Lenka,Cernikova
EPI_ISL_12150658	Czech Republic	2022-Jan-12	A/chicken/Czech Republic/913/2022	State Veterinary Institute Prague	State Veterinary Institute Prague	Alexander,Nagy;Martina,Stara;Lenka,Cernikova
EPI_ISL_12150661	Czech Republic	2022-Jan-29	A/mute swan/Czech Republic/2755/2022	State Veterinary Institute Prague	State Veterinary Institute Prague	na
EPI_ISL_12150664	Czech Republic	2021-Dec-18	A/goose/Czech Republic/25322-179/2021	State Veterinary Institute Prague	State Veterinary Institute Prague	Alexander,Nagy;Martina,Stara;Lenka,Cernikova
EPI_ISL_12150666	Czech Republic	2021-Dec-18	A/goose/Czech Republic/25322-205/2021	State Veterinary Institute Prague	State Veterinary Institute Prague	Alexander,Nagy;Martina,Stara;Lenka,Cernikova

EPI_ISL_12176837	Albania	2022-Mar-01	A/pelecanus_crispus/Albania/D383-22_22VIR3125-2/2022	Institute of Public Health	Istituto Zooprofilattico Sperimentale delle Venezie	Lika, A.; Boci, J.; Shkodra, E.; Barbierato, G.; Zecchin, B.; Fusaro, A.; Schivo, A.; Salviato, A.; Palumbo, E.; Giussani, E.; Monne, I.; Terregino, C.
EPI_ISL_12177629	Netherlands	2022-Apr-05	A/black-backed gull/Netherlands/22006711-001/2022	Wageningen Bioveterinary Research	Wageningen Bioveterinary Research	Beerens, Nancy; Harders, Frank; Pritz-Verschuren, Sylvia; Roose, Marit; Venema, Sandra; Germeraad, Evelien; Engelsma, Marc; Heutink, Rene
EPI_ISL_12210335	Belgium	2022-Mar-19	A/Falco_peregrinus/Belgium/4055_0002/2022	Sciensano - Animal Infectious Diseases	Sciensano, Department of Animal Infectious Diseases	Van Borm, Steven; Roupie, Virginie; Lambrecht, Benedicte; Mathijs, Elisabeth; Steensels, Mieke
EPI_ISL_12215416	Netherlands	2022-Mar-23	A/greylag goose/Netherlands/22005844-002/2022	Wageningen Bioveterinary Research	Wageningen Bioveterinary Research	Beerens, Nancy; Harders, Frank; Pritz-Verschuren, Sylvia; Roose, Marit; Venema, Sandra; Germeraad, Evelien; Engelsma, Marc; Heutink, Rene
EPI_ISL_12223688	Czech Republic	2021-Dec-18	A/grey heron/Czech Republic/25338-1/2021	State Veterinary Institute Prague	State Veterinary Institute Prague	Alexander,Nagy;Martina,Stara;Lenka,Cernikova
EPI_ISL_12223734	Czech Republic	2021-Dec-18	A/grey heron/Czech Republic/25338-2/2021	State Veterinary Institute Prague	State Veterinary Institute Prague	Alexander,Nagy;Martina,Stara;Lenka,Cernikova
EPI_ISL_12324302	Czech Republic	2022-Feb-09	A/duck/Czech Republic/3306-1/2022	State Veterinary Institute Prague	State Veterinary Institute Prague	Nagy,Alexander;Cernikova,Lenka;Stara,Martina
EPI_ISL_12325210	Czech Republic	2022-Feb-09	A/chicken/Czech Republic/3306-2/2022	State Veterinary Institute Prague	State Veterinary Institute Prague	Nagy,Alexander;Cernikova,Lenka;Stra,Martina
EPI_ISL_12325995	Czech Republic	2021-Dec-18	A/goose/Czech Republic/25322-229/2021	State Veterinary Institute Prague	State Veterinary Institute Prague	Alexander,Nagy;Martina,Stara;Lenka,Cernikova
EPI_ISL_12471655	Greece	2022-Feb-24	A/pelican/Greece/41-TR-313_22VIR3126-1/2022	Thessalonica Veterinary Centre (TVC)	Istituto Zooprofilattico Sperimentale delle Venezie	Georgiades, G.; Ragias, B.; Gkolia, A.; Anthopoulou, E.; Barbierato, G.; Zecchin, B.; Fusaro, A.; Schivo, A.; Salviato, A.; Palumbo, E.; Giussani, E.; Monne, I.; Terregino, C.
EPI_ISL_12471656	Greece	2022-Mar-12	A/pelican/Greece/69_CL_22VIR3126-10/2022	Thessalonica Veterinary Centre (TVC)	Istituto Zooprofilattico Sperimentale delle Venezie	Georgiades, G.; Ragias, B.; Gkolia, A.; Anthopoulou, E.; Barbierato, G.; Zecchin, B.; Fusaro, A.; Schivo, A.; Salviato, A.; Palumbo, E.; Giussani, E.; Monne, I.; Terregino, C.
EPI_ISL_12471657	Greece	2022-Mar-15	A/pelican/Greece/72_CL_22VIR3126-11/2022	Thessalonica Veterinary Centre (TVC)	Istituto Zooprofilattico Sperimentale delle Venezie	Georgiades, G.; Ragias, B.; Gkolia, A.; Anthopoulou, E.; Barbierato, G.; Zecchin, B.; Fusaro, A.; Schivo, A.; Salviato, A.; Palumbo, E.; Giussani, E.; Monne, I.; Terregino, C.
EPI_ISL_12471658	Greece	2022-Feb-24	A/pelican/Greece/41_AL1_22VIR3126-2/2022	Thessalonica Veterinary Centre (TVC)	Istituto Zooprofilattico Sperimentale delle Venezie	Georgiades, G.; Ragias, B.; Gkolia, A.; Anthopoulou, E.; Barbierato, G.; Zecchin, B.; Fusaro, A.; Schivo, A.; Salviato, A.; Palumbo, E.; Giussani, E.; Monne, I.; Terregino, C.
EPI_ISL_12471659	Greece	2022-Feb-24	A/pelican/Greece/41_AL2_22VIR3126-3/2022	Thessalonica Veterinary Centre (TVC)	Istituto Zooprofilattico Sperimentale delle Venezie	Georgiades, G.; Ragias, B.; Gkolia, A.; Anthopoulou, E.; Barbierato, G.; Zecchin, B.; Fusaro, A.; Schivo, A.; Salviato, A.; Palumbo, E.; Giussani, E.; Monne, I.; Terregino, C.

EPI_ISL_12471660	Greece	2022-Mar-10	A/pelican/Greece/64_KI_22VIR3126-6/2022	Thessalonica Veterinary Centre (TVC)	Istituto Zooprofilattico Sperimentale delle Venezie	Georgiades, G.; Ragias, B.; Gkolia, A.; Anthopoulou, E.; Barbierato, G.; Zecchin, B.; Fusaro, A.; Schivo, A.; Salviato, A.; Palumbo, E.; Giussani, E.; Monne, I.; Terregino, C.
EPI_ISL_12471661	Greece	2022-Mar-10	A/pelican/Greece/64_SP_22VIR3126-7/2022	Thessalonica Veterinary Centre (TVC)	Istituto Zooprofilattico Sperimentale delle Venezie	Georgiades, G.; Ragias, B.; Gkolia, A.; Anthopoulou, E.; Barbierato, G.; Zecchin, B.; Fusaro, A.; Schivo, A.; Salviato, A.; Palumbo, E.; Giussani, E.; Monne, I.; Terregino, C.
EPI_ISL_12471662	Greece	2022-Mar-10	A/pelican/Greece/64_LI_22VIR3126-8/2022	Thessalonica Veterinary Centre (TVC)	Istituto Zooprofilattico Sperimentale delle Venezie	Georgiades, G.; Ragias, B.; Gkolia, A.; Anthopoulou, E.; Barbierato, G.; Zecchin, B.; Fusaro, A.; Schivo, A.; Salviato, A.; Palumbo, E.; Giussani, E.; Monne, I.; Terregino, C.
EPI_ISL_12471663	Greece	2022-Mar-10	A/pelican/Greece/64_TR_22VIR3126-9/2022	Thessalonica Veterinary Centre (TVC)	Istituto Zooprofilattico Sperimentale delle Venezie	Georgiades, G.; Ragias, B.; Gkolia, A.; Anthopoulou, E.; Barbierato, G.; Zecchin, B.; Fusaro, A.; Schivo, A.; Salviato, A.; Palumbo, E.; Giussani, E.; Monne, I.; Terregino, C.
EPI_ISL_12474793	Netherlands	2022-Apr-17	A/barnacle goose/Netherlands/22007405-004/2022	Wageningen Bioveterinary Research	Wageningen Bioveterinary Research	Beerens, Nancy; Harders, Frank; Pritz-Verschuren, Sylvia; Roose, Marit; Venema, Sandra; Germeraad, Evelien; Engelsma, Marc; Heutink, Rene
EPI_ISL_12474794	Netherlands	2022-Apr-11	A/greylag goose /Netherlands/22006859-001/2022	Wageningen Bioveterinary Research	Wageningen Bioveterinary Research	Beerens, Nancy; Harders, Frank; Pritz-Verschuren, Sylvia; Roose, Marit; Venema, Sandra; Germeraad, Evelien; Engelsma, Marc; Heutink, Rene
EPI_ISL_12512879	Netherlands	2022-Apr-06	A/Black-headed gull/Netherlands/2/2022	Erasmus Medical Center	Erasmus Medical Center	na
EPI_ISL_12514425	Netherlands	2022-Apr-14	A/Black-headed gull/Netherlands/3/2022	Erasmus Medical Center	Erasmus Medical Center	na
EPI_ISL_12514442	Netherlands	2022-Apr-14	A/Black-headed gull/Netherlands/4/2022	Erasmus Medical Center	Erasmus Medical Center	na
EPI_ISL_12514483	Netherlands	2022-Apr-14	A/Caspian Gull/Netherlands/3/2022	Erasmus Medical Center	Erasmus Medical Center	na
EPI_ISL_12514532	Netherlands	2022-Apr-11	A/Common raven/Netherlands/1/2022	Erasmus Medical Center	Erasmus Medical Center	na
EPI_ISL_12514573	Netherlands	2022-Apr-13	A/Song Thrush/Netherlands/1/2022	Erasmus Medical Center	Erasmus Medical Center	na
EPI_ISL_12514622	Netherlands	2022-Apr-14	A/Common raven/Netherlands/2/2022	Erasmus Medical Center	Erasmus Medical Center	na
EPI_ISL_12514681	Netherlands	2022-Apr-14	A/European Herring Gull/Netherlands/3/2022	Erasmus Medical Center	Erasmus Medical Center	na
EPI_ISL_12754531	Romania	2022-Mar-18	A/laying_hen/Romania/11343_22VIR4106-1/2022	Institute for Diagnosis & Animal Health (IDAH)	Istituto Zooprofilattico Sperimentale Delle Venezie	Barbuceanu, F; Onita, I.; Neicut, A.; Motiu, R.; Burlacu, R.; Zecchin, B.; Barbierato, G.; Fusaro, A.; Schivo, A.; Salviato, A.; Palumbo, E.; Giussani, E.; Monne, I.; Terregino, C.

EPI_ISL_12754532	Romania	2022-Mar-17	A/pelican/Romania/11335_22VIR4106-2/2022	Institute for Diagnosis & Animal Health (IDAH)	Istituto Zooprofilattico Sperimentale Delle Venezie	Barbuceanu, F; Onita, I.; Neicut, A.; Motiu, R.; Burlacu, R.; Zecchin, B.; Barbierato, G.; Fusaro, A.; Schivo, A.; Salviato, A.; Palumbo, E.; Giussani, E.; Monne, I.; Terregino, C.
EPI_ISL_12754533	Romania	2022-Mar-17	A/pelican/Romania/11334_22VIR4106-3/2022	Institute for Diagnosis & Animal Health (IDAH)	Istituto Zooprofilattico Sperimentale Delle Venezie	Barbuceanu, F; Onita, I.; Neicut, A.; Motiu, R.; Burlacu, R.; Zecchin, B.; Barbierato, G.; Fusaro, A.; Schivo, A.; Salviato, A.; Palumbo, E.; Giussani, E.; Monne, I.; Terregino, C.
EPI_ISL_12754534	Romania	2022-Mar-26	A/laying_hen/Romania/11562_22VIR4106-4/2022	Institute for Diagnosis & Animal Health (IDAH)	Istituto Zooprofilattico Sperimentale Delle Venezie	Barbuceanu, F; Onita, I.; Neicut, A.; Motiu, R.; Burlacu, R.; Zecchin, B.; Barbierato, G.; Fusaro, A.; Schivo, A.; Salviato, A.; Palumbo, E.; Giussani, E.; Monne, I.; Terregino, C.
EPI_ISL_13026099	Luxembourg	2021-Nov-24	A/Greylag goose/Luxembourg/21217773/2021	Laboratoire de Médecine vétérinaire de l'Etat	Luxembourg Institute of Health	Aurélie Sausy, Chantal Snoeck
EPI_ISL_13026104	Luxembourg	2021-Nov-24	A/Canada goose/Luxembourg/21217776/2021	Laboratoire de Médecine vétérinaire de l'Etat	Luxembourg Institute of Health	Aurélie Sausy, Chantal Snoeck
EPI_ISL_13026105	Luxembourg	2021-Dec-09	A/Canada goose/Luxembourg/21237199/2021	Laboratoire de Médecine vétérinaire de l'Etat	Luxembourg Institute of Health	Aurélie Sausy, Chantal Snoeck
EPI_ISL_13026106	Luxembourg	2021-Dec-10	A/Canada goose/Luxembourg/21239614/2021	Laboratoire de Médecine vétérinaire de l'Etat	Luxembourg Institute of Health	Aurélie Sausy, Chantal Snoeck
EPI_ISL_13026236	Luxembourg	2021-Dec-14	A/Grey heron/Luxembourg/21243177/2021	Laboratoire de Médecine vétérinaire de l'Etat	Luxembourg Institute of Health	Aurélie Sausy, Chantal Snoeck
EPI_ISL_13026237	Luxembourg	2021-Dec-14	A/Great cormorant/Luxembourg/21243185/2021	Laboratoire de Médecine vétérinaire de l'Etat	Luxembourg Institute of Health	Aurélie Sausy, Chantal Snoeck
EPI_ISL_13026245	Luxembourg	2022-Jan-17	A/Canada goose/Luxembourg/22012198/2022	Laboratoire de Médecine vétérinaire de l'Etat	Luxembourg Institute of Health	Aurélie Sausy, Chantal Snoeck
EPI_ISL_13026246	Luxembourg	2022-Feb-04	A/Barnacle goose/Luxembourg/22033922/2022	Laboratoire de Médecine vétérinaire de l'Etat	Luxembourg Institute of Health	Aurélie Sausy, Chantal Snoeck
EPI_ISL_13201050	Netherlands	2022-Apr-29	A/Lesser Black-backed Gull/Netherlands/1/2022	Erasmus Medical Center	Erasmus Medical Center	na
EPI_ISL_13201074	Netherlands	2022-Mar-05	A/European polecat/Netherlands/1/2022	Erasmus Medical Center	Erasmus Medical Center	na
EPI_ISL_13245602	Iceland	2021-Oct-25	A/white-tailed eagle/Iceland/2022AI02104/2021	Icelandic Institute of Natural History	Friedrich-Loeffler-Institut	na

EPI_ISL_13246267	Iceland	2022-Apr-14	A/chicken/Iceland/2022AI02564/2022	University of Iceland, Keldur	Friedrich-Loeffler-Institut	na
EPI_ISL_13246657	Iceland	2022-Apr-14	A/chicken/Iceland/2022AI02565/2022	University of Iceland, Keldur	Friedrich-Loeffler-Institut	na
EPI_ISL_13300319	Netherlands	2022-May-26	A/brant goose/Netherlands/22009881-002/2022	Wageningen Bioveterinary Research	Wageningen Bioveterinary Research	Beerens, Nancy; Harders, Frank; Pritz-Verschuren, Sylvia; Roose, Marit; Venema, Sandra; Germeraad, Evelien; Engelsma, Marc; Heutink, Rene
EPI_ISL_13300324	Netherlands	2022-May-27	A/white stork/Netherlands/22009973-002/2022	Wageningen Bioveterinary Research	Wageningen Bioveterinary Research	Beerens, Nancy; Harders, Frank; Pritz-Verschuren, Sylvia; Roose, Marit; Venema, Sandra; Germeraad, Evelien; Engelsma, Marc; Heutink, Rene
EPI_ISL_13338081	Sweden	2022-May-25	A/Northern gannet/Sweden/SVA220525SZ0402/FB001671/O-2022	National Veterinary Institute, SVA	National Veterinary Institute	na
EPI_ISL_13360259	Netherlands	2021-Nov-07	A/chicken/Netherlands/21038165-006010/2021	Wageningen Bioveterinary Research	Wageningen Bioveterinary Research	Beerens, Nancy; Harders, Frank; Pritz-Verschuren, Sylvia; Roose, Marit; Venema, Sandra; Germeraad, Evelien; Engelsma, Marc; Heutink, Rene
EPI_ISL_13370915	United Kingdom	2022-Mar-26	A/domestic_duck/England/032919/2022	Animal and Plant Health Agency (APHA)	Animal and Plant Health Agency (APHA)	na
EPI_ISL_13370918	United Kingdom	2022-Apr-04	A/domestic_duck/England/041295/2022	Animal and Plant Health Agency (APHA)	Animal and Plant Health Agency (APHA)	na
EPI_ISL_13370924	United Kingdom	2022-Apr-21	A/chicken/England/053826/2022	Animal and Plant Health Agency (APHA)	Animal and Plant Health Agency (APHA)	na
EPI_ISL_13370925	United Kingdom	2022-May-05	A/chicken/England/063896/2022	Animal and Plant Health Agency (APHA)	Animal and Plant Health Agency (APHA)	na
EPI_ISL_13429290	Netherlands	2022-Jun-03	A/Caspian Gull/Netherlands/4/2022	Erasmus Medical Center	Erasmus Medical Center	na
EPI_ISL_13429291	Netherlands	2022-Jun-06	A/Sandwich Tern/Netherlands/8/2022	Erasmus Medical Center	Erasmus Medical Center	na
EPI_ISL_13429292	Netherlands	2022-Jun-06	A/Sandwich Tern/Netherlands/5/2022	Erasmus Medical Center	Erasmus Medical Center	na
EPI_ISL_13429293	Netherlands	2022-Jun-03	A/Black-headed gull/Netherlands/5/2022	Erasmus Medical Center	Erasmus Medical Center	na
EPI_ISL_13429294	Netherlands	2022-Jun-03	A/Black-headed gull/Netherlands/6/2022	Erasmus Medical Center	Erasmus Medical Center	na
EPI_ISL_13429295	Netherlands	2022-Jun-04	A/Sandwich Tern/Netherlands/1/2022	Erasmus Medical Center	Erasmus Medical Center	na
EPI_ISL_13429296	Netherlands	2022-Jun-03	A/Sandwich Tern/Netherlands/2/2022	Erasmus Medical Center	Erasmus Medical Center	na
EPI_ISL_13429297	Netherlands	2022-Jun-03	A/Sandwich Tern/Netherlands/3/2022	Erasmus Medical Center	Erasmus Medical Center	na
EPI_ISL_13429298	Netherlands	2022-Jun-03	A/Common Tern/Netherlands/1/2022	Erasmus Medical Center	Erasmus Medical Center	na

EPI_ISL_13429299	Netherlands	2022-Jun-06	A/Sandwich Tern/Netherlands/6/2022	Erasmus Medical Center	Erasmus Medical Center	na
EPI_ISL_13429300	Netherlands	2022-Jun-06	A/Sandwich Tern/Netherlands/7/2022	Erasmus Medical Center	Erasmus Medical Center	na
EPI_ISL_13453559	United Kingdom	2022-May-18	A/domestic_duck/England/073865/2022	Animal and Plant Health Agency (APHA)	Animal and Plant Health Agency (APHA)	na
EPI_ISL_13453560	United Kingdom	2022-May-29	A/chicken/Scotland/078873/2022	Animal and Plant Health Agency (APHA)	Animal and Plant Health Agency (APHA)	na
EPI_ISL_13453565	United Kingdom	2022-Mar-17	A/buzzard/Scotland/043118/2022	Animal and Plant Health Agency (APHA)	Animal and Plant Health Agency (APHA)	na
EPI_ISL_13453567	United Kingdom	2022-May-31	A/turkey/England/081395/2022	Animal and Plant Health Agency (APHA)	Animal and Plant Health Agency (APHA)	na
EPI_ISL_13453568	United Kingdom	2022-Jun-05	A/turkey/England/081490/2022	Animal and Plant Health Agency (APHA)	Animal and Plant Health Agency (APHA)	na
EPI_ISL_13486807	United Kingdom	2022-Mar-01	A/Common_buzzard/Scotland/020817/2022	Animal and Plant Health Agency (APHA)	Animal and Plant Health Agency (APHA)	na
EPI_ISL_13486869	United Kingdom	2022-Mar-07	A/Greylag_goose/Scotland/024915/2022	Animal and Plant Health Agency (APHA)	Animal and Plant Health Agency (APHA)	na
EPI_ISL_13514370	Belgium	2022-Jun-04	A/Thalasseus sandvicensis/Belgium/7473_0009/2022	Sciensano - Animal Infectious Diseases	Sciensano, Department of Animal Infectious Diseases	Van Borm, Steven; Roupie, Virginie; Lambrecht, Benedicte; Mathijs, Elisabeth; Steensels, Mieke
EPI_ISL_13514375	Belgium	2022-Jun-20	A/Gallus gallus/Belgium/8092_0002/2022	Sciensano - Animal Infectious Diseases	Sciensano, Department of Animal Infectious Diseases	Van Borm, Steven; Roupie, Virginie; Lambrecht, Benedicte; Mathijs, Elisabeth; Steensels, Mieke
EPI_ISL_13519451	France	2022-May-11	A/gull/France/22P015977/2022	Anses (Ploufragan-Plouzané)	ANSES Agence Nationale De Securite Sanitaire De L'alimentation	na
EPI_ISL_13613219	Netherlands	2022-Jun-06	A/Greylag Goose/Netherlands/4/2022	Erasmus Medical Center	Erasmus Medical Center	na
EPI_ISL_13613220	Netherlands	2022-Jun-06	A/Greylag Goose/Netherlands/5/2022	Erasmus Medical Center	Erasmus Medical Center	na
EPI_ISL_13613221	Netherlands	2022-Jun-06	A/Greylag Goose/Netherlands/6/2022	Erasmus Medical Center	Erasmus Medical Center	na
EPI_ISL_13613222	Netherlands	2022-Jun-06	A/Greylag Goose/Netherlands/7/2022	Erasmus Medical Center	Erasmus Medical Center	na
EPI_ISL_13613223	Netherlands	2022-Jun-06	A/Greylag Goose/Netherlands/8/2022	Erasmus Medical Center	Erasmus Medical Center	na
EPI_ISL_13613224	Netherlands	2022-Jun-10	A/Common Tern/Netherlands/2/2022	Erasmus Medical Center	Erasmus Medical Center	na
EPI_ISL_13613225	Netherlands	2022-Jun-10	A/Black-headed gull/Netherlands/7/2022	Erasmus Medical Center	Erasmus Medical Center	na

EPI_ISL_13613226	Netherlands	2022-Jun-10	A/Black-headed gull/Netherlands/8/2022	Erasmus Medical Center	Erasmus Medical Center	na
EPI_ISL_13613227	Netherlands	2022-Jun-10	A/Black-headed gull/Netherlands/9/2022	Erasmus Medical Center	Erasmus Medical Center	na
EPI_ISL_13613228	Netherlands	2022-Jun-12	A/Sandwich Tern/Netherlands/9/2022	Erasmus Medical Center	Erasmus Medical Center	na
EPI_ISL_13613229	Netherlands	2022-Jun-12	A/Sandwich Tern/Netherlands/10/2022	Erasmus Medical Center	Erasmus Medical Center	na
EPI_ISL_13613230	Netherlands	2022-Jun-13	A/Common Tern/Netherlands/3/2022	Erasmus Medical Center	Erasmus Medical Center	na
EPI_ISL_13692620	Russian Federation	2022-Feb-01	A/turkey/Stavropol/211-1V/2022	Center of Hygiene and Epidemiology in Stavropol Territory	State Research Center of Virology and Biotechnology (VECTOR)	na
EPI_ISL_13692621	Russian Federation	2022-Feb-01	A/turkey/Stavropol/211-4V/2022	Center of Hygiene and Epidemiology in Stavropol Territory	State Research Center of Virology and Biotechnology (VECTOR)	na
EPI_ISL_13692622	Russian Federation	2022-Feb-01	A/turkey/Stavropol/211-7V/2022	State Research Center of Virology and Biotechnology (VECTOR)	State Research Center of Virology and Biotechnology (VECTOR)	na
EPI_ISL_13692623	Russian Federation	2022-Feb-01	A/turkey/Stavropol/211-12V/2022	Center of Hygiene and Epidemiology in Stavropol Territory	State Research Center of Virology and Biotechnology (VECTOR)	na
EPI_ISL_13692624	Russian Federation	2022-Feb-01	A/turkey/Stavropol/211-12V/2022	State Research Center of Virology and Biotechnology (VECTOR)	State Research Center of Virology and Biotechnology (VECTOR)	na
EPI_ISL_13692625	Russian Federation	2022-Feb-01	A/turkey/Stavropol/211-15V/2022	State Research Center of Virology and Biotechnology (VECTOR)	State Research Center of Virology and Biotechnology (VECTOR)	na
EPI_ISL_13692626	Russian Federation	2022-Feb-01	A/turkey/Stavropol/211-18V/2022	Center of Hygiene and Epidemiology in Stavropol Territory	State Research Center of Virology and Biotechnology (VECTOR)	na
EPI_ISL_13692627	Russian Federation	2022-Feb-01	A/turkey/Stavropol/211-18V/2022	State Research Center of Virology and Biotechnology (VECTOR)	State Research Center of Virology and Biotechnology (VECTOR)	na
EPI_ISL_13692628	Russian Federation	2022-Feb-01	A/turkey/Stavropol/211-5V/2022	State Research Center of Virology and Biotechnology (VECTOR)	State Research Center of Virology and Biotechnology (VECTOR)	na
EPI_ISL_13692629	Russian Federation	2022-Feb-01	A/turkey/Stavropol/211-9V/2022	State Research Center of Virology and Biotechnology (VECTOR)	State Research Center of Virology and Biotechnology (VECTOR)	na
EPI_ISL_13692630	Russian Federation	2022-Apr-21	A/crow/Khabarovsk/216-11V/2022	Center of Hygiene and Epidemiology in Khabarovsk Territory	State Research Center of Virology and Biotechnology (VECTOR)	na

EPI_ISL_13692631	Russian Federation	2022-Apr-21	A/crow/Khabarovsk/216-11V/2022	State Research Center of Virology and Biotechnology (VECTOR)	State Research Center of Virology and Biotechnology (VECTOR)	na
EPI_ISL_13692632	Russian Federation	2022-Apr-21	A/crow/Khabarovsk/216-12V/2022	Center of Hygiene and Epidemiology in Khabarovsk Territory	State Research Center of Virology and Biotechnology (VECTOR)	na
EPI_ISL_13692633	Russian Federation	2022-Apr-21	A/crow/Khabarovsk/216-12V/2022	State Research Center of Virology and Biotechnology (VECTOR)	State Research Center of Virology and Biotechnology (VECTOR)	na
EPI_ISL_13692634	Russian Federation	2022-Apr-21	A/crow/Khabarovsk/216-13V/2022	Center of Hygiene and Epidemiology in Khabarovsk Territory	State Research Center of Virology and Biotechnology (VECTOR)	na
EPI_ISL_13692635	Russian Federation	2022-Apr-21	A/crow/Khabarovsk/216-13V/2022	State Research Center of Virology and Biotechnology (VECTOR)	State Research Center of Virology and Biotechnology (VECTOR)	na
EPI_ISL_13692637	Russian Federation	2022-Mar-14	A/dalmatian pelican/Astrakhan/213-1V/2022	State Research Center of Virology and Biotechnology (VECTOR)	State Research Center of Virology and Biotechnology (VECTOR)	na
EPI_ISL_13692638	Russian Federation	2022-Mar-14	A/dalmatian pelican/Astrakhan/213-2V/2022	State Research Center of Virology and Biotechnology (VECTOR)	State Research Center of Virology and Biotechnology (VECTOR)	na
EPI_ISL_13778462	Netherlands	2022-Jun-17	A/Lesser Black-backed Gull/Netherlands/2/2022	Erasmus Medical Center	Erasmus Medical Center	na
EPI_ISL_13778464	Netherlands	2022-Jun-19	A/European Herring Gull/Netherlands/4/2022	Erasmus Medical Center	Erasmus Medical Center	na
EPI_ISL_13778466	Netherlands	2022-Jun-19	A/Oystercatcher/Netherlands/2/2022	Erasmus Medical Center	Erasmus Medical Center	na
EPI_ISL_13778467	Netherlands	2022-Jun-19	A/Sandwich Tern/Netherlands/11/2022	Erasmus Medical Center	Erasmus Medical Center	na
EPI_ISL_13778468	Netherlands	2022-Jun-07	A/European Herring Gull/Netherlands/5/2022	Erasmus Medical Center	Erasmus Medical Center	na
EPI_ISL_13778469	Netherlands	2022-Jun-07	A/European Herring Gull/Netherlands/6/2022	Erasmus Medical Center	Erasmus Medical Center	na
EPI_ISL_13778474	Netherlands	2022-Jun-07	A/European Herring Gull/Netherlands/7/2022	Erasmus Medical Center	Erasmus Medical Center	na
EPI_ISL_13778475	Netherlands	2022-Jun-07	A/Sandwich Tern/Netherlands/12/2022	Erasmus Medical Center	Erasmus Medical Center	na
EPI_ISL_13778476	Netherlands	2022-Jun-07	A/Sandwich Tern/Netherlands/13/2022	Erasmus Medical Center	Erasmus Medical Center	na
EPI_ISL_13778477	Netherlands	2022-Jun-16	A/Common Tern/Netherlands/8/2022	Erasmus Medical Center	Erasmus Medical Center	na

EPI_ISL_13778478	Netherlands	2022-Jun-24	A/Sandwich Tern/Netherlands/14/2022	Erasmus Medical Center	Erasmus Medical Center	na
EPI_ISL_13778479	Netherlands	2022-Jun-24	A/Common Tern/Netherlands/9/2022	Erasmus Medical Center	Erasmus Medical Center	na
EPI_ISL_13778522	Netherlands	2022-Jun-24	A/Sandwich Tern/Netherlands/15/2022	Erasmus Medical Center	Erasmus Medical Center	na
EPI_ISL_13778523	Netherlands	2022-Jun-20	A/Common Tern/Netherlands/4/2022	Erasmus Medical Center	Erasmus Medical Center	na
EPI_ISL_13778524	Netherlands	2022-Jun-20	A/Common Tern/Netherlands/5/2022	Erasmus Medical Center	Erasmus Medical Center	na
EPI_ISL_13778525	Netherlands	2022-Jun-20	A/Common Tern/Netherlands/6/2022	Erasmus Medical Center	Erasmus Medical Center	na
EPI_ISL_13778526	Netherlands	2022-Jun-20	A/Common Tern/Netherlands/7/2022	Erasmus Medical Center	Erasmus Medical Center	na
EPI_ISL_13876272	Russian Federation	2022-May-28	A/chicken/Ryazan/224-1V/2022	Center of Hygiene and Epidemiology in Ryazan Oblast	State Research Center of Virology and Biotechnology (VECTOR)	na
EPI_ISL_13876273	Russian Federation	2022-May-28	A/chicken/Ryazan/224-1V/2022	State Research Center of Virology and Biotechnology (VECTOR)	State Research Center of Virology and Biotechnology (VECTOR)	na
EPI_ISL_13902858	Netherlands	2022-Jul-03	A/Eurasian Curlew/Netherlands/2/2022	Erasmus Medical Center	Erasmus Medical Center	na
EPI_ISL_13955171	Czech Republic	2022-Feb-04	A/chicken/Czech Republic/2968/2022	State Veterinary Institute Prague	State Veterinary Institute Prague	Alexander,Nagy;Lenka,Cernikova;Martina,Stara
EPI_ISL_13955204	Czech Republic	2022-Apr-13	A/chicken/Czech Republic/8028-1/2022	State Veterinary Institute Prague	State Veterinary Institute Prague	Alexander,Nagy;Lenka,Cernikova;Martina,Stara
EPI_ISL_13967740	Czech Republic	2022-Apr-13	A/chicken/Czech Republic/8028-2/2022	State Veterinary Institute Prague	State Veterinary Institute Prague	Alexander,Nagy; Lenka,Cernikova; Martina,Stara
EPI_ISL_13969422	United Kingdom	2022-Mar-28	A/Goose/Scotland/036879/2022	Animal and Plant Health Agency (APHA)	Animal and Plant Health Agency (APHA)	na
EPI_ISL_13969423	United Kingdom	2022-Apr-29	A/Gull/Scotland/060376/2022	Animal and Plant Health Agency (APHA)	Animal and Plant Health Agency (APHA)	na
EPI_ISL_13969424	United Kingdom	2022-Jun-06	A/Gannet/Scotland/084490/2022	Animal and Plant Health Agency (APHA)	Animal and Plant Health Agency (APHA)	na
EPI_ISL_13969426	United Kingdom	2022-Jun-18	A/Gannet/Scotland/090910/2022	Animal and Plant Health Agency (APHA)	Animal and Plant Health Agency (APHA)	na
EPI_ISL_13969428	United Kingdom	2022-Apr-14	A/Greylag_goose/England/247696/2022	Animal and Plant Health Agency (APHA)	Animal and Plant Health Agency (APHA)	na
EPI_ISL_13969430	United Kingdom	2022-Apr-10	A/Mute_swan/Wales/058560/2022	Animal and Plant Health Agency (APHA)	Animal and Plant Health Agency (APHA)	na
EPI_ISL_14064983	United Kingdom	2022-Jul-04	A/chicken/Scotland/093091/2022	Animal and Plant Health Agency (APHA)	Animal and Plant Health Agency (APHA)	na

EPI_ISL_14064984	United Kingdom	2022-Jul-06	A/chicken/England/093459/2022	Animal and Plant Health Agency (APHA)	Animal and Plant Health Agency (APHA)	na
EPI_ISL_14064985	United Kingdom	2022-Jul-06	A/Domestic_goose/England/093469/2022	Animal and Plant Health Agency (APHA)	Animal and Plant Health Agency (APHA)	na
EPI_ISL_14163711	Netherlands	2022-Jul-07	A/Common Tern/Netherlands/10/2022	Erasmus Medical Center	Erasmus Medical Center	na
EPI_ISL_14163712	Netherlands	2022-Jul-07	A/European Herring Gull/Netherlands/8/2022	Erasmus Medical Center	Erasmus Medical Center	na
EPI_ISL_14163713	Netherlands	2022-Jul-08	A/Caspian Gull/Netherlands/5/2022	Erasmus Medical Center	Erasmus Medical Center	na
EPI_ISL_14163714	Netherlands	2022-Jul-04	A/European Herring Gull/Netherlands/9/2022	Erasmus Medical Center	Erasmus Medical Center	na
EPI_ISL_14163715	Netherlands	2022-Jul-12	A/Greylag Goose/Netherlands/9/2022	Erasmus Medical Center	Erasmus Medical Center	na
EPI_ISL_14171729	Netherlands	2022-Jul-11	A/Eurasian Spoonbill/Netherlands/1A/2022	Erasmus Medical Center	Erasmus Medical Center	na
EPI_ISL_14171741	Netherlands	2022-Jul-11	A/Eurasian Spoonbill/Netherlands/1B/2022	Erasmus Medical Center	Erasmus Medical Center	na
EPI_ISL_14233919	Netherlands	2022-Jul-24	A/Eurasian Spoonbill/Netherlands/3/2022	Erasmus Medical Center	Erasmus Medical Center	na
EPI_ISL_14233920	Netherlands	2022-Jul-15	A/Common Tern/Netherlands/17/2022	Erasmus Medical Center	Erasmus Medical Center	na
EPI_ISL_14233921	Netherlands	2022-Jul-15	A/Common Tern/Netherlands/18/2022	Erasmus Medical Center	Erasmus Medical Center	na
EPI_ISL_14233922	Netherlands	2022-Jul-19	A/European Herring Gull/Netherlands/11/2022	Erasmus Medical Center	Erasmus Medical Center	na
EPI_ISL_14233923	Netherlands	2022-Jul-15	A/Caspian Gull/Netherlands/7/2022	Erasmus Medical Center	Erasmus Medical Center	na
EPI_ISL_14233924	Netherlands	2022-Jul-22	A/Common Tern/Netherlands/19/2022	Erasmus Medical Center	Erasmus Medical Center	na
EPI_ISL_14233925	Netherlands	2022-Jul-22	A/Common Tern/Netherlands/20/2022	Erasmus Medical Center	Erasmus Medical Center	na
EPI_ISL_14233926	Netherlands	2022-Jul-14	A/European Herring Gull/Netherlands/10/2022	Erasmus Medical Center	Erasmus Medical Center	na
EPI_ISL_14233927	Netherlands	2022-Jul-14	A/Common Tern/Netherlands/11/2022	Erasmus Medical Center	Erasmus Medical Center	na
EPI_ISL_14233928	Netherlands	2022-Jul-14	A/Common Tern/Netherlands/13/2022	Erasmus Medical Center	Erasmus Medical Center	na

EPI_ISL_14233929	Netherlands	2022-Jul-14	A/Common Tern/Netherlands/12/2022	Erasmus Medical Center	Erasmus Medical Center	na
EPI_ISL_14233930	Netherlands	2022-Jul-14	A/Common Tern/Netherlands/14/2022	Erasmus Medical Center	Erasmus Medical Center	na
EPI_ISL_14233931	Netherlands	2022-Jul-14	A/Common Tern/Netherlands/15/2022	Erasmus Medical Center	Erasmus Medical Center	na
EPI_ISL_14233932	Netherlands	2022-Jul-14	A/Common Tern/Netherlands/16/2022	Erasmus Medical Center	Erasmus Medical Center	na
EPI_ISL_14233944	Netherlands	2022-Jul-15	A/Caspian Gull/Netherlands/6/2022	Erasmus Medical Center	Erasmus Medical Center	na
EPI_ISL_14388346	Belgium	2021-Dec-23	A/Tyto_alba/Belgium/334_0012/2021	Sciensano - Animal Infectious Diseases	Sciensano, Department of Animal Infectious Diseases	Van Borm, Steven; Vandenbussche, Frank; Roupie, Virginie; Lambrecht, Benedicte; Steensels, Mieke
EPI_ISL_14388434	Belgium	2021-Dec-24	A/Buteo_buteo/belgium/334_0013/2021	Sciensano - Animal Infectious Diseases	Sciensano, Department of Animal Infectious Diseases	Van Borm, Steven; Vandenbussche, Frank; Roupie, Virginie; Lambrecht, Benedicte; Steensels, Mieke
EPI_ISL_14389133	Belgium	2022-Jan-06	A/Larus_argentatus/Belgium/595_0008/2022	Sciensano - Animal Infectious Diseases	Sciensano, Department of Animal Infectious Diseases	Van Borm, Steven; Vandenbussche, Frank; Roupie, Virginie; Lambrecht, Benedicte; Steensels, Mieke
EPI_ISL_14389148	Belgium	2022-Jan-24	A/Tachybaptus_ruficollis/Belgium/1234_0008/2022	Sciensano - Animal Infectious Diseases	Sciensano, Department of Animal Infectious Diseases	Van Borm, Steven; Vandenbussche, Frank; Roupie, Virginie; Lambrecht, Benedicte; Steensels, Mieke
EPI_ISL_14389524	Belgium	2022-Jan-26	A/Anser_anser_domesticus/Belgium/1668_0016/2022	Sciensano - Animal Infectious Diseases	Sciensano, Department of Animal Infectious Diseases	Van Borm, Steven; Vandenbussche, Frank; Roupie, Virginie; Lambrecht, Benedicte; Steensels, Mieke
EPI_ISL_14390100	Belgium	2022-Jan-21	A/Larus_canus/Belgium/1668_0019/2022	Sciensano - Animal Infectious Diseases	Sciensano, Department of Animal Infectious Diseases	Van Borm, Steven; Vandenbussche, Frank; Roupie, Virginie; Lambrecht, Benedicte; Steensels, Mieke
EPI_ISL_14390385	Belgium	2022-Feb-02	A/Phalacrocorax_carbo/Belgium/1734_0002/2022	Sciensano - Animal Infectious Diseases	Sciensano, Department of Animal Infectious Diseases	Van Borm, Steven; Vandenbussche, Frank; Roupie, Virginie; Lambrecht, Benedicte; Steensels, Mieke
EPI_ISL_14391865	Belgium	2022-Feb-20	A/Buteo_buteo/Belgium/2606_0006/2022	Sciensano - Animal Infectious Diseases	Sciensano, Department of Animal Infectious Diseases	Van Borm, Steven; Vandenbussche, Frank; Roupie, Virginie; Lambrecht, Benedicte; Steensels, Mieke
EPI_ISL_14392127	Belgium	2022-Feb-17	A/Branta_leucopsis/Belgium/2606_0009/2022	Sciensano - Animal Infectious Diseases	Sciensano, Department of Animal Infectious Diseases	Van Borm, Steven; Vandenbussche, Frank; Roupie, Virginie; Lambrecht, Benedicte; Steensels, Mieke
EPI_ISL_14393097	Netherlands	2022-Apr-02	A/Fox/Netherlands/EMC4/2022	Erasmus Medical Center	Erasmus Medical Center	na
EPI_ISL_14393115	Netherlands	2022-Feb-09	A/Fox/Netherlands/EMC5/2022	Erasmus Medical Center	Erasmus Medical Center	na
EPI_ISL_14393126	Netherlands	2022-Feb-15	A/Fox/Netherlands/EMC6/2022	Erasmus Medical Center	Erasmus Medical Center	na
EPI_ISL_14393465	Belgium	2022-Apr-07	A/Branta_canadensis/Belgium/4821_0001/2022	Sciensano - Animal Infectious Diseases	Sciensano, Department of Animal Infectious Diseases	Van Borm, Steven; Vandenbussche, Frank; Roupie, Virginie; Lambrecht, Benedicte; Steensels, Mieke
EPI_ISL_14393671	Belgium	2022-Apr-13	A/Branta_canadensis/Belgium/5177_0003/2022	Sciensano - Animal Infectious Diseases	Sciensano, Department of Animal Infectious Diseases	Van Borm, Steven; Vandenbussche, Frank; Roupie, Virginie; Lambrecht, Benedicte; Steensels, Mieke

EPI_ISL_14469689	Netherlands	2022-Jul-29	A/Common Tern/Netherlands/21/2022	Erasmus Medical Center	Erasmus Medical Center	na
EPI_ISL_14469690	Netherlands	2022-Jul-29	A/Common Tern/Netherlands/22/2022	Erasmus Medical Center	Erasmus Medical Center	na
EPI_ISL_14469691	Netherlands	2022-Jul-29	A/Common Tern/Netherlands/23/2022	Erasmus Medical Center	Erasmus Medical Center	na
EPI_ISL_14469692	Netherlands	2022-Jul-29	A/Common Tern/Netherlands/24/2022	Erasmus Medical Center	Erasmus Medical Center	na
EPI_ISL_14469693	Netherlands	2022-Jul-30	A/Common Tern/Netherlands/25/2022	Erasmus Medical Center	Erasmus Medical Center	na
EPI_ISL_14493899	Belgium	2022-Apr-16	A/Vulpes_vulpes/Belgium/8660_0016/2022	Sciensano - Animal Infectious Diseases	Sciensano, Department of Animal Infectious Diseases	Van Borm, Steven; Mathijs, Elisabeth; Roupie, Virginie; Vervaeke, Muriel; Lambrecht, Benedicte; Steensels, Mieke
EPI_ISL_14494351	Belgium	2022-Jul-08	A/Larus_argentatus/Belgium/9013_0001/2022	Sciensano - Animal Infectious Diseases	Sciensano, Department of Animal Infectious Diseases	Van Borm, Steven; Mathijs, Elisabeth; Roupie, Virginie; Lambrecht, Benedicte; Steensels, Mieke
EPI_ISL_14494796	Belgium	2022-Jun-16	A/Vulpes_vulpes/Belgium/9031_0008/2022	Sciensano - Animal Infectious Diseases	Sciensano, Department of Animal Infectious Diseases	Van Borm, Steven; Mathijs, Elisabeth; Roupie, Virginie; Vervaeke, Muriel; Lambrecht, Benedicte; Steensels, Mieke
EPI_ISL_14494933	Belgium	2022-Aug-03	A/Gallus_gallus/Belgium/9548_0001/2022	Sciensano - Animal Infectious Diseases	Sciensano, Department of Animal Infectious Diseases	Van Borm, Steven; Mathijs, Elisabeth; Roupie, Virginie; Lambrecht, Benedicte; Steensels, Mieke
EPI_ISL_14497321	Netherlands	2022-Aug-10	A/Eurasian Spoonbill/Netherlands/4/2022	Erasmus Medical Center	Erasmus Medical Center	na
EPI_ISL_14497837	United Kingdom	2022-Jul-26	A/domestic_duck/England/100990/2022	Animal and Plant Health Agency (APHA)	Animal and Plant Health Agency (APHA)	na
EPI_ISL_14497855	United Kingdom	2022-Aug-05	A/domestic_duck/England/104859/2022	Animal and Plant Health Agency (APHA)	Animal and Plant Health Agency (APHA)	na
EPI_ISL_14497870	United Kingdom	2022-Aug-07	A/domestic_duck/England/105414/2022	Animal and Plant Health Agency (APHA)	Animal and Plant Health Agency (APHA)	na
EPI_ISL_14497885	United Kingdom	2022-Aug-09	A/domestic_goose/England/105863/2022	Animal and Plant Health Agency (APHA)	Animal and Plant Health Agency (APHA)	na
EPI_ISL_14702899	France	2022-Jul-13	A/nothern_gannet/France/22P019331/2022	Anses (Ploufragan-Plouzané)	ANSES Agence Nationale De Securite Sanitaire De L'alimentation	na
EPI_ISL_14722960	France	2022-May-09	A/vulture/France/22P018210/2022	Anses (Ploufragan-Plouzané)	ANSES Agence Nationale De Securite Sanitaire De L'alimentation	na
EPI_ISL_14810369	Sweden	2022-Jun-29	A/porpoise/Sweden/SVA220712SZ0367/FB002184/O-2022	National Veterinary Institute, SVA	National Veterinary Institute	na

EPI_ISL_14811896	Sweden	2022-Jun-20	A/northern gannet/Sweden/SVA220623SZ0232/FB001982/O-2022	National Veterinary Institute, SVA	National Veterinary Institute	na
EPI_ISL_14811929	Sweden	2022-Jun-15	A/northern gannet/Sweden/SVA220623SZ0232/FB001984/N-2022	National Veterinary Institute, SVA	National Veterinary Institute	na
EPI_ISL_14841913	France	2022-Jul-06	A/mallard/22P019377/France/2022	Anses (Ploufragan-Plouzané)	ANSES Agence Nationale De Securite Sanitaire De L'alimentation	na
EPI_ISL_14857053	Russian Federation	2022-Jun-20	A/chicken/Kursk/230-2V/2022	Center of Hygiene and Epidemiology in Kursk Oblast	State Research Center of Virology and Biotechnology (VECTOR)	na
EPI_ISL_14857054	Russian Federation	2022-Jun-20	A/chicken/Kursk/230-4V/2022	Center of Hygiene and Epidemiology in Kursk Oblast	State Research Center of Virology and Biotechnology (VECTOR)	na
EPI_ISL_14857055	Russian Federation	2022-Jun-20	A/chicken/Kursk/230-5V/2022	Center of Hygiene and Epidemiology in Kursk Oblast	State Research Center of Virology and Biotechnology (VECTOR)	na
EPI_ISL_14857056	Russian Federation	2022-Jun-20	A/chicken/Kursk/230-7V/2022	Center of Hygiene and Epidemiology in Kursk Oblast	State Research Center of Virology and Biotechnology (VECTOR)	na
EPI_ISL_14857057	Russian Federation	2022-Jun-20	A/chicken/Kursk/230-8V/2022	Center of Hygiene and Epidemiology in Kursk Oblast	State Research Center of Virology and Biotechnology (VECTOR)	na
EPI_ISL_14857058	Russian Federation	2022-Jun-20	A/chicken/Kursk/230-10V/2022	Center of Hygiene and Epidemiology in Kursk Oblast	State Research Center of Virology and Biotechnology (VECTOR)	na
EPI_ISL_14857059	Russian Federation	2022-Jun-16	A/chicken/Kursk/230-15V/2022	Center of Hygiene and Epidemiology in Kursk Oblast	State Research Center of Virology and Biotechnology (VECTOR)	na
EPI_ISL_14857060	Russian Federation	2022-Jul-11	A/chicken/Kursk/234-19V/2022	Center of Hygiene and Epidemiology in Kursk Oblast	State Research Center of Virology and Biotechnology (VECTOR)	na
EPI_ISL_14857061	Russian Federation	2022-Jul-11	A/quail/Kursk/234-20V/2022	Center of Hygiene and Epidemiology in Kursk Oblast	State Research Center of Virology and Biotechnology (VECTOR)	na
EPI_ISL_14857062	Russian Federation	2022-Jul-28	A/chicken/Magadan/235-57V/2022	FBUZ Center of hygiene and epidemiology	State Research Center of Virology and Biotechnology (VECTOR)	na
EPI_ISL_14857063	Russian Federation	2022-Jul-28	A/chicken/Magadan/235-58V/2022	FBUZ Center of hygiene and epidemiology	State Research Center of Virology and Biotechnology (VECTOR)	na

EPI_ISL_14857064	Russian Federation	2022-Jul-28	A/chicken/Magadan/235-59V/2022	FBUZ Center of hygiene and epidemiology	State Research Center of Virology and Biotechnology (VECTOR)	na
EPI_ISL_14857065	Russian Federation	2022-Jul-28	A/chicken/Magadan/235-60V/2022	FBUZ Center of hygiene and epidemiology	State Research Center of Virology and Biotechnology (VECTOR)	na
EPI_ISL_14864637	United Kingdom	2022-Jun-16	A/goose/England/317610/2022	Animal and Plant Health Agency (APHA)	Animal and Plant Health Agency (APHA)	na
EPI_ISL_14864646	United Kingdom	2022-Jul-26	A/Canada_goose/England/320660/2022	Animal and Plant Health Agency (APHA)	Animal and Plant Health Agency (APHA)	na
EPI_ISL_14864650	United Kingdom	2022-Aug-26	A/turkey/England/111923/2022	Animal and Plant Health Agency (APHA)	Animal and Plant Health Agency (APHA)	na
EPI_ISL_14917968	Poland	2022-Jul-13	A/common_murre/Poland/MB151/2022	National Veterinary Research Institut Poland, PIWet-PIB	National Veterinary Research Institut Poland, PIWet-PIB	Swieton E., Smietanka K.
EPI_ISL_14917979	Poland	2022-May-30	A/herring_gull/Poland/MB138/2022	National Veterinary Research Institut Poland, PIWet-PIB	National Veterinary Research Institut Poland, PIWet-PIB	Swieton E., Smietanka K.
EPI_ISL_14917999	Poland	2022-May-30	A/black-headed_gull/Poland/MB139/2022	National Veterinary Research Institut Poland, PIWet-PIB	National Veterinary Research Institut Poland, PIWet-PIB	Swieton E., Smietanka K.
EPI_ISL_14933724	France	2022-Aug-24	A/duck/France/22P020165/2022	Anses (Ploufragan-Plouzané)	ANSES Agence Nationale De Securite Sanitaire De L'alimentation	na
EPI_ISL_15038822	United Kingdom	2022-Sep-02	A/chicken/England/114595/2022	Animal and Plant Health Agency (APHA)	Animal and Plant Health Agency (APHA)	na
EPI_ISL_15069398	Netherlands	2022-Aug-10	A/European Herring Gull/Netherlands/12/2022	Erasmus Medical Center	Erasmus Medical Center	na
EPI_ISL_15069399	Netherlands	2022-Aug-10	A/Black-headed gull/Netherlands/10/2022	Erasmus Medical Center	Erasmus Medical Center	na
EPI_ISL_15069400	Netherlands	2022-Aug-10	A/Mute Swan/Netherlands/1/2022	Erasmus Medical Center	Erasmus Medical Center	na
EPI_ISL_15069401	Netherlands	2022-Aug-12	A/Common Tern/Netherlands/26/2022	Erasmus Medical Center	Erasmus Medical Center	na
EPI_ISL_15088295	Netherlands	2022-Aug-17	A/Common Buzzard/Netherlands/1/2022	Erasmus Medical Center	Erasmus Medical Center	na
EPI_ISL_15088296	Netherlands	2022-Aug-19	A/European Herring Gull/Netherlands/13/2022	Erasmus Medical Center	Erasmus Medical Center	na
EPI_ISL_15088297	Netherlands	2022-Aug-23	A/European Herring Gull/Netherlands/14/2022	Erasmus Medical Center	Erasmus Medical Center	na
EPI_ISL_15088298	Netherlands	2022-Aug-23	A/Northern Gannet/Netherlands/1/2022	Erasmus Medical Center	Erasmus Medical Center	na

EPI_ISL_15088299	Netherlands	2022-Aug-26	A/Greylag Goose/Netherlands/10/2022	Erasmus Medical Center	Erasmus Medical Center	na
EPI_ISL_15088300	Netherlands	2022-Aug-26	A/Greylag Goose/Netherlands/11/2022	Erasmus Medical Center	Erasmus Medical Center	na
EPI_ISL_15088301	Netherlands	2022-Aug-26	A/Greylag Goose/Netherlands/12/2022	Erasmus Medical Center	Erasmus Medical Center	na
EPI_ISL_15088302	Netherlands	2022-Aug-27	A/Greylag Goose/Netherlands/13/2022	Erasmus Medical Center	Erasmus Medical Center	na
EPI_ISL_15088303	Netherlands	2022-Aug-29	A/Lesser Black-backed Gull/Netherlands/3/2022	Erasmus Medical Center	Erasmus Medical Center	na
EPI_ISL_15088304	Netherlands	2022-Aug-29	A/European Herring Gull/Netherlands/15/2022	Erasmus Medical Center	Erasmus Medical Center	na
EPI_ISL_15088305	Netherlands	2022-Aug-29	A/Common Teal/Netherlands/1/2022	Erasmus Medical Center	Erasmus Medical Center	na
EPI_ISL_15088306	Netherlands	2022-Aug-18	A/Mallard/Netherlands/3/2022	Erasmus Medical Center	Erasmus Medical Center	na
EPI_ISL_15088307	Netherlands	2022-Aug-20	A/Mallard/Netherlands/4/2022	Erasmus Medical Center	Erasmus Medical Center	na
EPI_ISL_15088308	Netherlands	2022-Aug-21	A/Mallard/Netherlands/5/2022	Erasmus Medical Center	Erasmus Medical Center	na
EPI_ISL_15088309	Netherlands	2022-Aug-24	A/Mallard/Netherlands/6/2022	Erasmus Medical Center	Erasmus Medical Center	na
EPI_ISL_15115556	United Kingdom	2022-Aug-26	A/turkey/England/112259/2022	Animal and Plant Health Agency (APHA)	Animal and Plant Health Agency (APHA)	na
EPI_ISL_15267012	Netherlands	2022-Sep-04	A/Lesser Black-backed Gull/Netherlands/4/2022	Erasmus Medical Center	Erasmus Medical Center	na
EPI_ISL_15267013	Netherlands	2022-Jun-16	A/Eurasian Spoonbill/Netherlands/5/2022	Erasmus Medical Center	Erasmus Medical Center	na
EPI_ISL_15267014	Netherlands	2022-Jun-16	A/Eurasian Spoonbill/Netherlands/6/2022	Erasmus Medical Center	Erasmus Medical Center	na
EPI_ISL_15267016	Netherlands	2022-Jun-16	A/Eurasian Spoonbill/Netherlands/7/2022	Erasmus Medical Center	Erasmus Medical Center	na
EPI_ISL_15267017	Netherlands	2022-Aug-31	A/Eurasian Spoonbill/Netherlands/8/2022	Erasmus Medical Center	Erasmus Medical Center	na
EPI_ISL_15267018	Netherlands	2022-Aug-31	A/Greylag Goose/Netherlands/14/2022	Erasmus Medical Center	Erasmus Medical Center	na
EPI_ISL_15267019	Netherlands	2022-Sep-02	A/Greylag Goose/Netherlands/15/2022	Erasmus Medical Center	Erasmus Medical Center	na

EPI_ISL_15267020	Netherlands	2022-Sep-02	A/Greylag Goose/Netherlands/16/2022	Erasmus Medical Center	Erasmus Medical Center	na
EPI_ISL_15267021	Netherlands	2022-Sep-02	A/Common Tern/Netherlands/27/2022	Erasmus Medical Center	Erasmus Medical Center	na
EPI_ISL_15267022	Netherlands	2022-Sep-03	A/European Herring Gull/Netherlands/16/2022	Erasmus Medical Center	Erasmus Medical Center	na
EPI_ISL_15267023	Netherlands	2022-Jul-13	A/Northern Gannet/Netherlands/2/2022	Erasmus Medical Center	Erasmus Medical Center	na
EPI_ISL_15267024	Netherlands	2022-Jul-10	A/Northern Gannet/Netherlands/3/2022	Erasmus Medical Center	Erasmus Medical Center	na
EPI_ISL_15267025	Netherlands	2022-Jul-09	A/Northern Gannet/Netherlands/4/2022	Erasmus Medical Center	Erasmus Medical Center	na
EPI_ISL_15267026	Netherlands	2022-Jun-26	A/Northern Gannet/Netherlands/5/2022	Erasmus Medical Center	Erasmus Medical Center	na
EPI_ISL_15267027	Netherlands	2022-Jun-21	A/Northern Gannet/Netherlands/6/2022	Erasmus Medical Center	Erasmus Medical Center	na
EPI_ISL_15364788	Netherlands	2022-Sep-10	A/European Herring Gull/Netherlands/17/2022	Erasmus Medical Center	Erasmus Medical Center	na
EPI_ISL_15364789	Netherlands	2022-Sep-10	A/European Herring Gull/Netherlands/18/2022	Erasmus Medical Center	Erasmus Medical Center	na
EPI_ISL_15364790	Netherlands	2022-Sep-16	A/Greylag Goose/Netherlands/17/2022	Erasmus Medical Center	Erasmus Medical Center	na
EPI_ISL_15364792	Netherlands	2022-Sep-14	A/Mallard/Netherlands/11/2022	Erasmus Medical Center	Erasmus Medical Center	na
EPI_ISL_15364793	Netherlands	2022-Sep-19	A/Mallard/Netherlands/12/2022	Erasmus Medical Center	Erasmus Medical Center	na
EPI_ISL_15364794	Netherlands	2022-Sep-26	A/Mallard/Netherlands/13/2022	Erasmus Medical Center	Erasmus Medical Center	na
EPI_ISL_15364795	Netherlands	2022-Sep-08	A/Mallard/Netherlands/8/2022	Erasmus Medical Center	Erasmus Medical Center	na
EPI_ISL_15364796	Netherlands	2022-Sep-11	A/Mallard/Netherlands/9/2022	Erasmus Medical Center	Erasmus Medical Center	na
EPI_ISL_15364797	Netherlands	2022-Sep-19	A/Mute Swan/Netherlands/2/2022	Erasmus Medical Center	Erasmus Medical Center	na
EPI_ISL_15535272	Poland	2022-Sep-20	A/domestic_goose/Poland/H397-N/2022	National Veterinary Research Institut Poland, PIWet-PIB	National Veterinary Research Institut Poland, PIWet-PIB	Świętoń E., Śmietanka K.
EPI_ISL_15542438	Spain	2022-Sep-23	A/CastillaLaMancha/223739/2022	Instituto de Salud Carlos III	Instituto de Salud Carlos III	Pozo,F; Iglesias-Caballero, M.; Molinero,M.; Camarero,S.; Mart?n,V.; Reyes,N.; V?zquez-Mor?n,S.; Casas,I.
EPI_ISL_15579535	Netherlands	2022-Sep-21	A/European Herring Gull/Netherlands/19/2022	Erasmus Medical Center	Erasmus Medical Center	na

EPI_ISL_15579536	Netherlands	2022-Sep-25	A/Greylag Goose/Netherlands/18/2022	Erasmus Medical Center	Erasmus Medical Center	na
EPI_ISL_15579537	Netherlands	2022-Sep-26	A/Barnacle Goose/Netherlands/19/2022	Erasmus Medical Center	Erasmus Medical Center	na
EPI_ISL_15579538	Netherlands	2022-Sep-28	A/Eurasian Curlew/Netherlands/4/2022	Erasmus Medical Center	Erasmus Medical Center	na
EPI_ISL_15579539	Netherlands	2022-Oct-02	A/European Herring Gull/Netherlands/20/2022	Erasmus Medical Center	Erasmus Medical Center	na
EPI_ISL_15579540	Netherlands	2022-Sep-21	A/Mallard/Netherlands/15/2022	Erasmus Medical Center	Erasmus Medical Center	na
EPI_ISL_15579543	Netherlands	2022-Oct-01	A/Mallard/Netherlands/18/2022	Erasmus Medical Center	Erasmus Medical Center	na
